# Bone dysplasia in Hutchinson‐Gilford progeria syndrome is associated with dysregulated differentiation and function of bone cell populations

**DOI:** 10.1111/acel.13903

**Published:** 2023-06-26

**Authors:** Wayne A. Cabral, Chris Stephan, Masahiko Terajima, Abhirami A. Thaivalappil, Owen Blanchard, Urraca L. Tavarez, Narisu Narisu, Tingfen Yan, Stephen M. Wincovitch, Yuki Taga, Mitsuo Yamauchi, Kenneth M. Kozloff, Michael R. Erdos, Francis S. Collins

**Affiliations:** ^1^ Molecular Genetics Section, Center for Precision Health Research National Human Genome Research Institute, NIH Bethesda Maryland USA; ^2^ Departments of Orthopedic Surgery and Biomedical Engineering University of Michigan Ann Arbor Michigan USA; ^3^ Division of Oral and Craniofacial Health Sciences, Adams School of Dentistry University of North Carolina Chapel Hill North Carolina USA; ^4^ Cytogenetics and Microscopy Core National Human Genome Research Institute, NIH Bethesda Maryland USA; ^5^ Nippi Research Institute of Biomatrix Ibaraki Japan

**Keywords:** bone dysplasia, Hutchinson‐Gilford progeria syndrome, lamin A/C, osteoblasts, osteoclasts

## Abstract

Hutchinson‐Gilford progeria syndrome (HGPS) is a premature aging disorder affecting tissues of mesenchymal origin. Most individuals with HGPS harbor a de novo c.1824C > T (p.G608G) mutation in the gene encoding lamin A (*LMNA*), which activates a cryptic splice donor site resulting in production of the toxic “progerin” protein. Clinical manifestations include growth deficiency, lipodystrophy, sclerotic dermis, cardiovascular defects, and bone dysplasia. Here we utilized the *Lmna* ^G609G^ knock‐in (KI) mouse model of HGPS to further define mechanisms of bone loss associated with normal and premature aging disorders. Newborn skeletal staining of KI mice revealed altered rib cage shape and spinal curvature, and delayed calvarial mineralization with increased craniofacial and mandibular cartilage content. MicroCT analysis and mechanical testing of adult femurs indicated increased fragility associated with reduced bone mass, recapitulating the progressive bone deterioration that occurs in HGPS patients. We investigated mechanisms of bone loss in KI mice at the cellular level in bone cell populations. Formation of wild‐type and KI osteoclasts from marrow‐derived precursors was inhibited by KI osteoblast‐conditioned media in vitro, suggesting a secreted factor(s) responsible for decreased osteoclasts on KI trabecular surfaces in vivo. Cultured KI osteoblasts exhibited abnormal differentiation characterized by reduced deposition and mineralization of extracellular matrix with increased lipid accumulation compared to wild‐type, providing a mechanism for altered bone formation. Furthermore, quantitative analyses of KI transcripts confirmed upregulation of adipogenic genes both in vitro and in vivo. Thus, osteoblast phenotypic plasticity, inflammation and altered cellular cross‐talk contribute to abnormal bone formation in HGPS mice.

## Graphical abstract

Hutchinson‐Gilford progeria syndrome is commonly referred to as a premature aging disorder characterized by development of atherosclerosis, lipodystrophy, and osteoporosis. Here we demonstrate that the bone phenotype results from abnormal osteoblast differentiation and altered interactions between bone cell populations leading to a low bone formation and turnover condition, distinct from senile osteoporosis, and more accurately described as an osteochondrodysplasia.

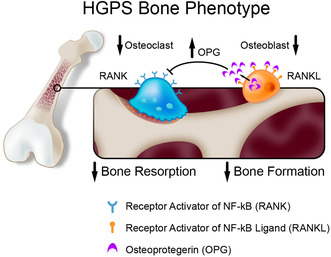

**Abbreviations**
BMD: bone mineral density
DAPI: 4′,6‐diamidino‐2‐phenylindole
ELISA: enzyme‐linked immunosorbent assay
H&E: hematoxylin and eosin
HGPS: Hutchinson–Gilford progeria syndrome
Hyl: hydroxylysine
KEGG: Kyoto Encyclopedia of Genes and Genomes
LMNA: Lamin A
LMNC: Lamin C
LOX: lysyl oxidase
MCSF: macrophage colony‐stimulating factor
Micro‐CT: micro computed tomography
MS/BS: mineralizing surface per bone surface
PPAR: peroxisome proliferator‐activated receptor
RANKL: receptor activator of nuclear factor kappa‐Β ligand
RNA: ribonucleic acid
RT‐PCR: reverse transcription‐polymerase chain reaction
Tb BV: trabecular bone volume
TRAcP: tartrate‐resistant acid phosphatase
TUNEL: terminal deoxynucleotidyl transferase dUTP nick end labeling
WNT: Wingless‐related integration site
ZMPSTE24: zinc metallopeptidase STE24

## 1 INTRODUCTION

Bone is a dynamic tissue that provides structural support and also serves a variety of essential systemic metabolic roles that regulate mineral homoeostasis, energy expenditure, and hematopoiesis through paracrine and endocrine signaling (Han et al., 2018). Under normal physiologic conditions bone homeostasis is maintained at equilibrium by the dual processes of formation, mediated by mesenchyme‐derived osteoblasts, and resorption, mediated by hematopoietic osteoclasts. Both cell types are derived from precursors residing in the bone marrow compartment and are recruited in response to local and systemic factors, a process dysregulated by genetic alterations, hormonal changes, pathological changes, or physiological aging. In addition to a decline in bone mass and load‐bearing capacity of the skeleton, an imbalance in bone deposition and resorption may be associated with altered responses to systemic humoral factors, reductions in mineral content and increased marrow adiposity. Factors most closely associated with various forms of primary or secondary osteoporosis, as especially occurs in aging related bone loss, are estimated to affect nearly two‐thirds of individuals over the age of 70 (Corrado et al., 2020).

Hutchinson‐Gilford progeria syndrome (HGPS) is a fatal autosomal dominant premature aging disorder that occurs in approximately 1 in 8 million births, and without treatment results in an average lifespan of 14.6 years (Hennekam, 2006). Although patients appear normal at birth, phenotypic features such as growth deficiency, alopecia, loss of subdermal fat, and sclerodermatous skin appear within the first year of life. The most serious aspects of the disease involve cardio‐ and cerebro‐vascular complications leading to myocardial infarction, heart failure or stroke (Gerhard‐Herman et al., 2012). HGPS also exhibits skeletal features that include relative mandibular and clavicular hypoplasia, coxa valga, joint contractures, and osteoporosis with an irregular pattern of long bone and cranial mineralization. Therefore, HGPS represents a potential model for accelerated aging‐related bone loss (Gordon et al., 2007).

Classic HGPS is caused by a de novo mutation (c.1824C → T, p.G608G) in the *LMNA* gene, encoding the A‐type lamins A, C, and AΔ10 (de Sandre‐Giovannoli et al., 2003; Eriksson et al., 2003). Lamins are type V intermediate filaments comprising a meshwork of proteins underlying the inner nuclear membrane, where they provide structural support and organization to the nuclear envelope. Lamins are also found throughout the nucleoplasm consistent with a role in DNA replication, transcription, cell division, and chromatin organization (Kubben & Misteli, 2017). Prelamin A is subject to post‐translational processing at its C‐terminus, including farnesylation of a CAAX motif, proteolysis of the terminal AAX sequence, carboxymethylation, and cleavage of the terminal 15 residues by the metalloprotease ZMPSTE24. In HGPS the final step of post‐translational processing of LMNA is inhibited due to activation of a cryptic splice donor site resulting in deletion of the ZMPSTE24 cleavage site, and subsequent accumulation of a permanently farnesylated internally deleted LMNA variant, termed progerin, within the nuclear lamina (Kubben & Misteli, 2017).

In contrast to constitutively expressed B‐type lamins, A‐type lamins are synthesized predominantly in differentiated cells, suggesting a role in the induction and maintenance of the differentiated state (Rober et al., 1989). Lamin A/C levels may also be involved in normal aging processes such as senile osteoporosis, as its expression in bone marrow cells declines with age in mice (Duque & Rivas, 2006). Furthermore, disease‐causing mutations in *LMNA* are believed to disrupt the balance between proliferation and differentiation of bone marrow mesenchymal stem cells (MSCs), leading to stem cell exhaustion and loss of tissue regeneration (Gotzmann & Foisner, 2006). Consistent with this proposal, *Lmna* ^−/−^ mice develop severe bone loss due to reduced osteogenic differentiation with increased adipogenic potential of MSCs, a phenotype associated with normal physiologic aging (Akter et al., 2009; Li et al., 2011). Severe loss of bone mass has also been reported in mouse models of HGPS, including those with LMNA processing defects and mice expressing the *Lmna* ^G609G^ knock‐in equivalent to the human *LMNA* G608G mutation (Bergo et al., 2002; Osorio et al., 2011; Zaghini et al., 2020). However, the relationship between bone loss in the absence of *Lmna* expression, and bone loss in the context of dominant negative progerin activity, remains to be clarified. In this study, we have utilized the *Lmna* ^G609G^ mouse model to further investigate pathomechanisms of bone dysplasia in HGPS. Our results suggest that osteoblast phenotypic plasticity, inflammation and altered cellular crosstalk result in a low bone turnover condition that is distinct from aging‐related osteoporosis.

## 2 RESULTS

We studied both heterozygous (*Lmna* ^G609G/+^) and homozygous (*Lmna* ^G609G/G609G^) mouse models of HGPS in this project. Matings between wild‐type (*Lmna* ^+/+^) and *Lmna* ^G609G/+^ mice generated offspring with normal Mendelian genetic distribution (*n* = 700). In contrast, we noted that matings between heterozygous mice generated fewer homozygous offspring than expected, resulting in an 11.4% rate of embryonic or perinatal lethality (*n* = 300). As previously reported, surviving heterozygous and homozygous pups exhibited an apparently normal phenotype at birth (Osorio et al., 2011; Zaghini et al., 2020). Whole skeleton staining of newborn mice, however, revealed subtle skeletal features that included narrower, bell‐shaped rib cages with delayed maxillary, mandibular and calvarial mineralization in both *Lmna* ^G609G/+^ and *Lmna* ^G609G/G609G^ compared to wild‐type (*Lmna* ^+/+^) littermates (Figure S1). Reduced mineral staining of the hyoid bone and increased ossification of tail vertebrae was also noted in heterozygous and homozygous pups, while calcification of the ribs and long bones appeared normal.

**FIGURE 1:**
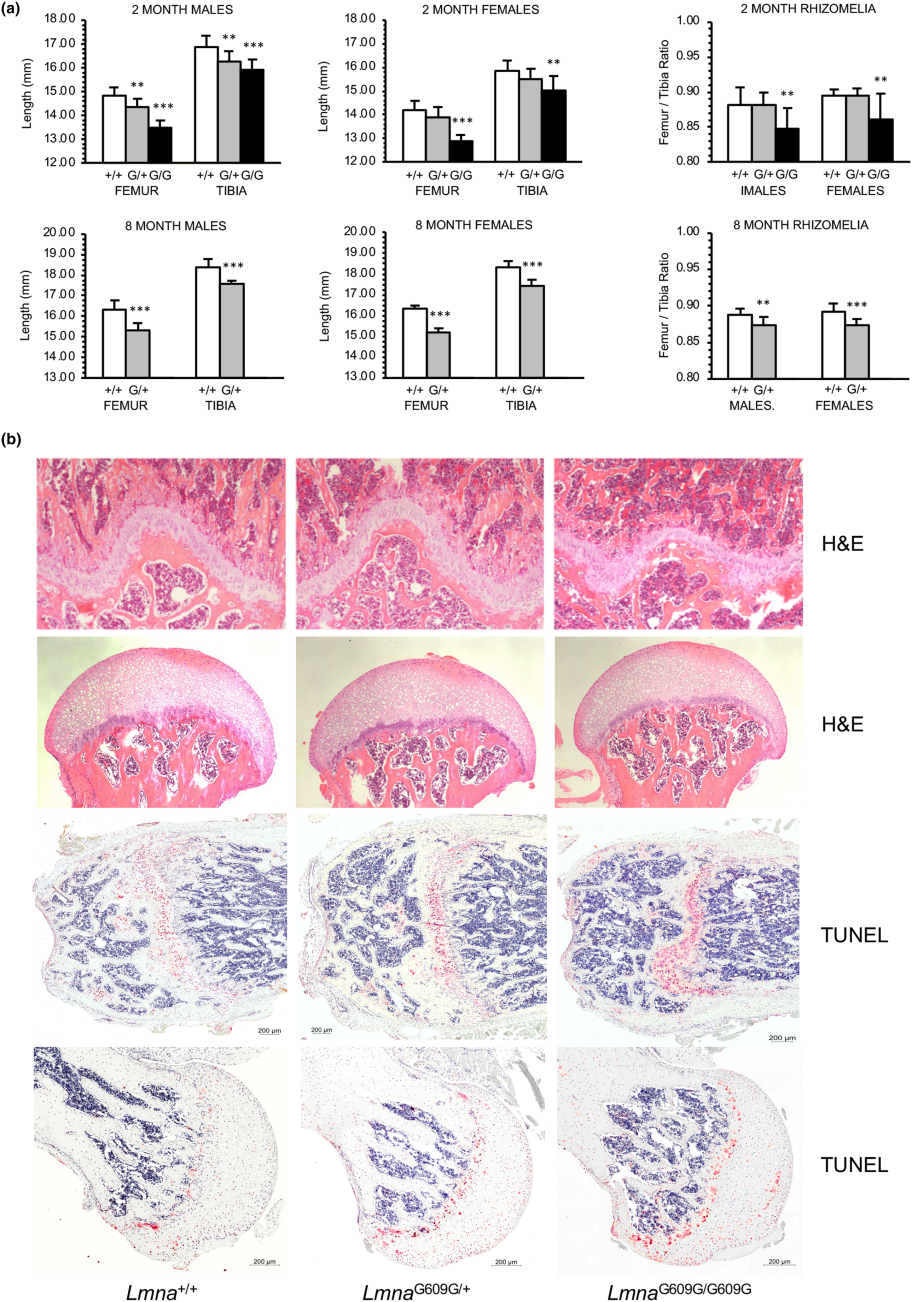
Abnormal bone structural parameters in *Lmna* ^G609G^ mice. (a) Eight‐week old homozygous (G/G) mice have significantly shorter femora and tibiae compared to wild‐type (+/+) and heterozygous (G/+) littermates. Shortening of femora in homozygous (G/G) mice is relatively greater than in tibiae resulting in rhizomelia. *N* = 10 for each gender and genotype; *p < 0.05; **p < 0.01; ***p < 0.001 (b) H&E staining reveals disorganization of distal growth plates and femoral head, especially in homozygous (*Lmna* ^G609G/G609G^) mice at 2 months of age. TUNEL staining of whole femora detects increased DNA damage in distal growth plate and femoral head chondrocytes in heterozygous (*Lmna* ^G609G/+^) and homozygous (*Lmna* ^G609G/G609G^) mice.

By 2 months of age quantitative changes in long bone structural parameters were observed in both heterozygous and homozygous mice. Morphometric measurements of *Lmna* ^G609G/+^ and *Lmna* ^G609G/G609G^ male mice, as well as *Lmna* ^G609G/G609G^ female mice, demonstrated that average femoral and tibial lengths were significantly decreased compared to wild‐type littermates (Figure 1a). In both male and female homozygotes, average tibial and femoral lengths were reduced 5% and 10%, respectively, versus wild‐type. The greater relative decrease in the ratio of femoral to tibial lengths in male and female homozygous mice, termed rhizomelia, has also been observed in some types of chondro‐ and osteo‐chondrodysplasias, which is suggestive of growth plate abnormalities. Accordingly, histologic analysis of 2 month‐old mouse femora revealed thinner, misshapen growth plates of the distal femur and femoral head of *Lmna* ^G609G/+^ and *Lmna* ^G609G/G609G^ mice, and was associated with increased DNA damage and/or apoptosis as demonstrated by increased TUNEL staining of growth plate chondrocytes (Figure 1b). The alterations found in long bone structural parameters of 2‐month homozygous mice were also observed in male and female heterozygous mice by 8 months of age, including decreased femoral and tibial lengths, and the development of rhizomelia (Figure 1a). These data suggest that dysregulated functioning of growth plate chondrocytes contributes to reduced longitudinal bone growth in mice expressing progerin. To assess appositional growth in progeroid mice dynamic histomorphometric analysis of cortical bone formation was performed (Figure S2). Dual labeling at 2 months of age failed to identify any significant differences in the extent of mineralizing surfaces (MS/BS), as well as mineral apposition (MAR) and bone formation (BFR) rates on periosteal surfaces, while endosteal MAR and BFR were minimally reduced in *Lmna* ^G609G/G609G^ mice versus wild‐type littermates by 27% (*p* < 0.09) and 33% (*p* < 0.08), respectively.

**FIGURE 2:**
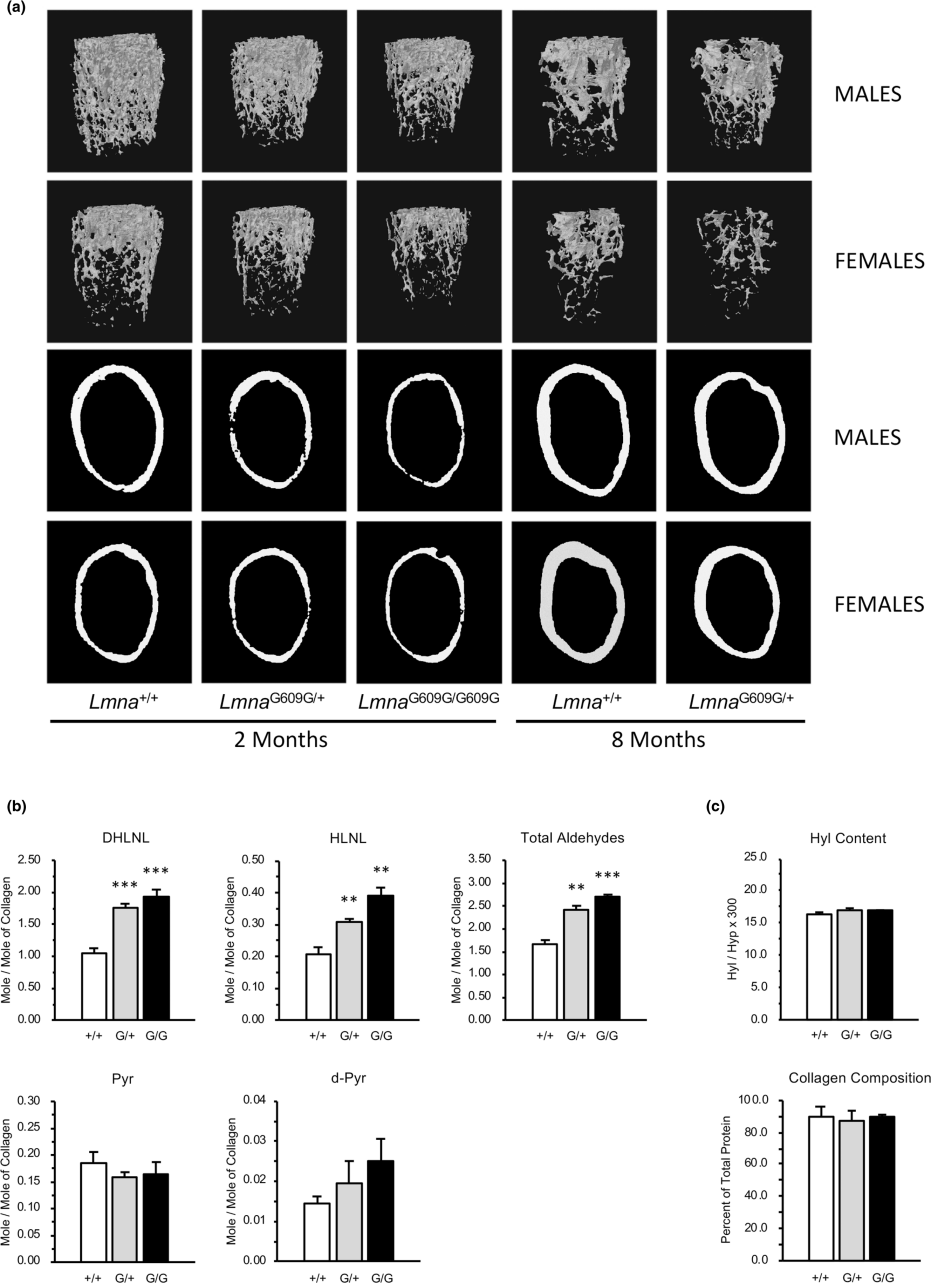
Expression of progerin alters femoral bone structural and material properties. (a) Reconstructed images from microCT analysis illustrate reduced trabecular and cortical bone volume in heterozygous (*Lmna* ^G609G/+^) and homozygous (*Lmna* ^G609G/G609G^) compared to wild‐type femora at 2 and 8 months of age. (b) Abnormal collagen cross‐link profiles are observed in 2 month‐old heterozygous (G/+) and homozygous (G/G) mice. Immature, divalent lysine‐derived (DHLNL: reduced dehydro‐dihydroxylysinonorleucine/it's ketoamine) and hydroxylysine‐derived (HLNL: reduced dehydro‐hydroxylysinonorleucine/it's ketoamine) crosslinks are increased relative to wild‐type mice. Total collagen aldehydes, catalyzed by LOX activity, are increased in progeroid mice. *p < 0.05, **p < 0.01, ***p < 0.001 versus *Lmna* ^+/+^ littermates (c) Total collagen content and extent of lysine hydroxylation (Hyl) are normal in *Lmna* ^G609G/+^ (G/+) and *Lmna* ^G609G/G609G^ (G/G) versus wild‐type (+/+) bone. *N* = 3/genotype.

Micro‐CT analyses of femora of 2‐month homozygous mice revealed additional structural alterations in cortical and trabecular bone tissues (Figure 2a, Table S1). Trabecular bone volume was decreased 21% (*p* < 0.05) and 35% (*p* < 0.001) due to reduced trabecular number and thickness, and trabecular mineral density was decreased 17% (*p* < 0.05) and 27% (*p* < 0.001) in females and males, respectively, compared to wild‐type mice. Femoral cortical bone mineral density was minimally reduced, while bone volume was decreased due to an 11%–12% decrease in cortical thickness (*p* < 0.001 for both genders). Similar structural alterations were found in femora of 8 month‐old *Lmna* ^G609G/+^ mice but were more severe in females than males. In contrast to the 19% decrease in the moment of inertia (MOI) calculated for 2‐month male (*p* < 0.05) and female (*p* < 0.001) homozygous femurs, the structural alterations in 8‐month heterozygous femora were insufficient to predict weaker physical parameters.

To assess the physical effects of structural changes in *Lmna* ^G609G/+^ and *Lmna* ^G609G/G609G^ femora, mechanical testing was performed by four‐point bending. Although stiffness was normal in 2 month‐old femora, both yield load and maximum load were reduced in male and female homozygous mice, whereas femurs from heterozygous mice exhibited normal mechanical parameters (Table S2). Notably, we identified gender‐specific reductions in some elastic and plastic properties of bone, including reductions in yield displacement, work to yield, post‐yield work, and total work required to break femora from male, but not female mice. These gender‐specific differences were also found in heterozygous mice at 8 months of age, including reduced yield displacement, post‐yield work, and total work required to break male femora. However, in contrast to 2‐month homozygous mice, we found that femora from 8‐month female heterozygous mice were less stiff and required lower load to fracture compared to wild‐type. Taken together, these changes in physical parameters demonstrate that the bone phenotype in *Lmna* ^G609G/G609G^ mice is more severe in males than females, and while homozygous mice do not develop brittle bones, they are more susceptible to breaking when force is applied.

Given the observation in reduced post‐yield work in 2‐month homozygous (*p* < 0.01) and 8‐month heterozygous (*p* < 0.05) femora, we next sought to determine if changes in matrix‐specific properties, in addition to the reduced structural parameters, contributed to decreased physical properties of *Lmna* ^G609G/+^ and *Lmna* ^G609G/G609G^ femora. Collagen cross‐link analysis of femora from 2 month‐old mice revealed significant alterations in *Lmna* ^G609G/+^ and *Lmna* ^G609G/G609G^ (Figure 2b). The concentrations of immature divalent cross‐links (DHLNL, dihydroxylysinonorleucine and HLNL, hydroxylysinonorleucine) were increased 64% (*p* < 0.001) and 85% (*p* < 0.001) in heterozygous and homozygous mice, respectively, and contributed to the greater number of total aldehydes involved in cross‐linking in bone collagen compared to wild‐type mice. The mature, trivalent cross‐links, Pyr (pyridinoline) and d‐Pyr (deoxypyridinoline), were not significantly different among these three groups. As a result, bone collagen from *Lmna* ^G609G/+^ and *Lmna* ^G609G/G609G^ mice contains more total cross‐links with a preponderance toward immature, divalent cross‐links. Mass spectrometric analyses of lysine post‐translational modifications (i.e., hydroxylation and glycosylation) and prolyl 3‐hydroxylation, however, showed no difference compared to wild‐type mice (Figure 2c, Table S3). Collagen composition was also similar among the groups.

**FIGURE 3:**
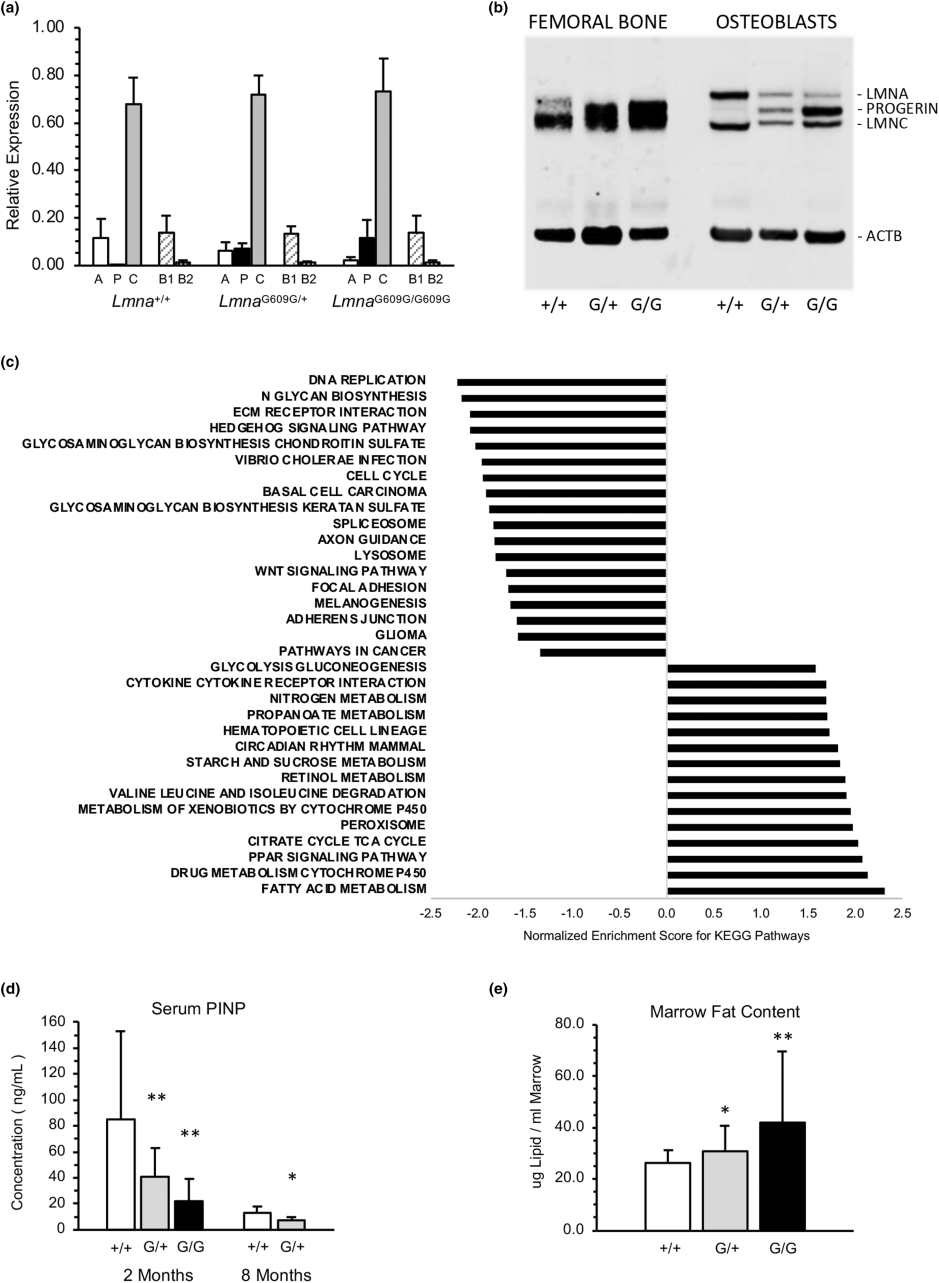
Expression of progerin alters signaling pathways required for osteogenesis. (a) Quantitation of A‐ and B‐type lamin transcripts in femoral tissue of 2‐month mice. Expression of B‐type lamins, *Lmnb1* (B1) and *Lmnb2* (B2) remains stable regardless of genotype. (b) Western analysis of A‐type lamins extracted from bone tissue and osteoblast cultures derived from wild‐type (*Lmna* ^+/+^, +/+), heterozygous (*Lmna* ^G609G/+^, G/+), and homozygous (*Lmna* ^G609G/G609G^, G/G) mice. (c) Gene set enrichment analysis of KEGG Pathways reveals positive enrichment for metabolic and cytokine activity and negative enrichment for cell–cell and cell‐matrix interactions. (d) Type I procollagen N‐propeptide (PINP), a marker of bone‐forming activity of osteoblasts, is decreased in serum of heterozygous (G/+) and homozygous (G/G) mice at 2 months of age and in heterozygous (G/+) mice at 8 months of age compared to wild‐type mice. *N* = 12 per genotype (six males, six females). (e) Marrow lipid content of heterozygous (G/+) and homozygous (G/G) mice at 2 months of age is increased versus wild‐type. *N* = 8 per genotype (four males, four females). *p < 0.05; **p < 0.01; ***p < 0.001.

Having characterized the bone phenotype in *Lmna* ^G609G^ mice, we investigated the mechanism(s) underlying the bone loss by analyzing gene expression in femoral bone tissue. Two‐month femurs from heterozygous and homozygous mice were dissected, with the epiphyses removed and marrow flushed prior to RNA and protein extraction, resulting in cortical bone with a gene expression profile enriched for osteocytes (~90%–95%), osteoblasts (~4%–6%), and osteoclasts (~1%–2%) (Schaffler & Kennedy, 2012). We first confirmed expression of A‐ and B‐type lamins by quantitative RT‐PCR and noted reduced lamin A and increased progerin transcripts between heterozygous and homozygous mice in a gene dosage‐dependent manner (Figure 3a). No differences in expression of lamins C, B1, and B2 were observed. Western analysis of protein extracted from pulverized bone tissue or from calvarial‐derived osteoblasts also revealed a correlation between *Lmna* ^G609G^ allele copy number and progerin accumulation (Figure 3b).

Differential expression analysis of RNA sequencing data revealed 254 genes (*p*adj < 0.05) with transcript levels that were altered by at least 1.5‐fold in cortical bone of both male and female 2‐month homozygous mice, compared to wild‐type. Subsequent gene set enrichment analysis (GSEA) revealed 33 distinct KEGG Pathways that were significantly up‐ or down‐regulated in *Lmna* ^G609G/G609G^ versus *Lmna* ^+/+^ (Figure 3c, Data File S1). Notable downregulated pathways included DNA replication and cell cycle control, synthesis of glycosaminoglycans, spliceosome function, extracellular matrix‐receptor interactions with their associated focal adhesion formation, and the WNT signaling pathway, which has been previously identified as dysregulated in HGPS (Choi et al., 2018; Hernandez et al., 2010; Sola‐Carvajal et al., 2019). Upregulated pathways that were identified involved fatty acid metabolism and PPAR signaling with the highest enrichment scores, cytokine‐receptor interactions that suggested the involvement of an inflammatory response in bone pathology, and activation of hematopoietic cell lineages. The altered pathways in *Lmna* ^G609G/G609G^ bone tissue were associated with reduced expression of several WNT signaling components, and genes required for osteogenic development including early (*Col1a1*, *Sp7*, *Alpl*), middle (*Bglap2*, *Sparc*), and late (*Phex*, *Dmp1*, *Sost*) markers of osteoblast differentiation (Figure S3a,b). In contrast we identified increased expression of genes involved in adipogenic differentiation and lipid homeostasis including *Pparg*, *Fabp4*, *Lpl*, and *Leptin* (Figure S3c). These tissue‐level gene expression patterns were consistent with reduced serum procollagen I N‐propeptide levels, a marker of osteoblast activity, and increased marrow fat content in heterozygous and homozygous mice (Figure 3d,e). Hence in 2‐month *Lmna* ^G609G/G609G^ bone tissue, the gene expression profile suggested activation of an adipogenic transcriptional program at the expense of osteogenesis.

Given the association between aging‐related inflammatory responses and alterations in bone remodeling leading to osteoporosis (Ginaldi et al., 2005), we examined whether pro‐inflammatory cytokine production was increased in *Lmna* ^G609G/G609G^ mice. Serum was collected from 2 month‐old mice and analyzed using an array‐based multiplex ELISA system. We found significantly increased levels of cytokines known to inhibit osteoblastogenesis, including IGFBP‐5, G‐CSF, and decorin, while the osteogenic cytokine leptin was decreased 80% in homozygous versus wild‐type mice (Figure S3d). Cytokines associated with increased bone resorption, such as IL6, MMP9, and the osteoclast precursor surface marker CD27 were also increased. However, several factors that either promote osteoblast development (PTX3, CXCL13, CXCL16) or inhibit bone resorption (CST3, OPG) were also increased and reflect the occurrence of a chronic stress‐induced inflammatory state in vivo, which is known to contribute to phenotypic plasticity in mesenchymal‐derived osteoprogenitors (Ambrosi et al., 2021).

**FIGURE 4:**
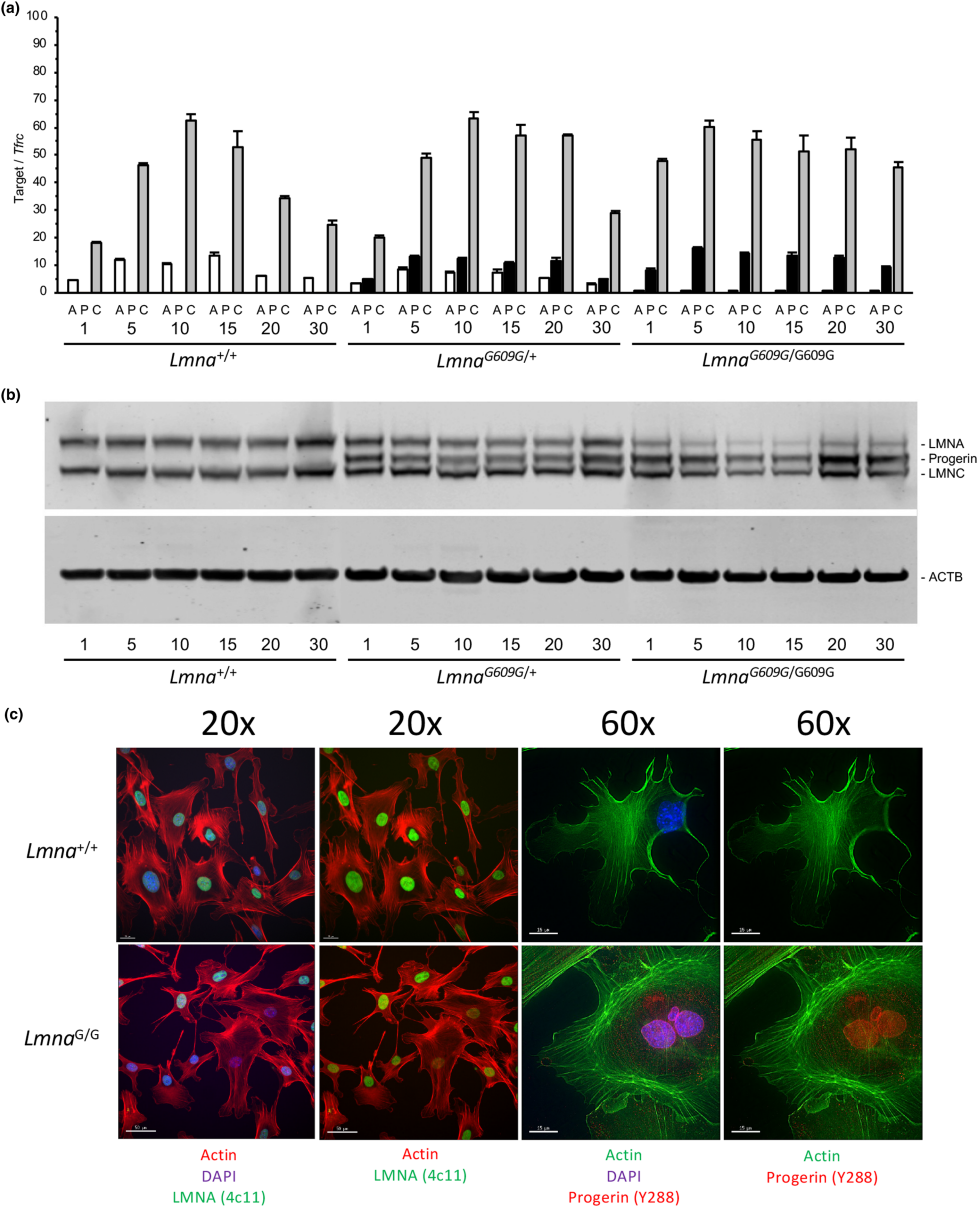
Expression of A‐type lamins in cultured murine osteoblasts. (a) Quantitative RT‐PCR of lamin A (A), progerin (P), and lamin C (C) transcripts from cultured osteoblasts at days 1, 5, 10, 15, 20, and 30 of osteogenic differentiation. (b) Immunoblots of cellular protein show increased accumulation of A‐type lamins at late stages of differentiation. (c) Left, Immunostaining demonstrates localization of A‐type lamins within the nucleus of cultured osteoblasts. Cells were stained to visualize nuclei (DAPI, blue), Actin (red), and A‐type lamins (green, antibody 4c11). Right, progerin‐specific antibody (Y288) reacts with the nucleoplasm, nuclear lamina, and cytoplasmic elements in cultured *Lmna* ^G609G/G609G^ (Lmna^G/G^) osteoblasts. Cells were stained to visualize nuclei (DAPI, blue), Actin (green), and progerin (red). Scale bars, 50 μm at 20× magnification; 15 μm at 60× magnification.

To determine if intrinsic factors contribute to altered differentiation in bone cell populations of *Lmna* ^G609G/G609G^ mice, pre‐osteoblasts were collected from calvaria of newborn mice for in vitro analyses. Quantitation of gene expression in cultured cells was consistent with a role for A‐type lamins in cellular differentiation. Prior to the addition of osteogenic media, we found relatively low levels of *Lmna* and *Lmnc* transcripts (Figure 4a) in wild‐type cultures. As differentiation proceeded, transcripts increased reaching maximum levels midway through the time course, then receding back to levels initially observed at the start of differentiation. We also found that for both *Lmna* ^G609G/+^ and *Lmna* ^G609G/G609G^ cultures expression of progerin resulted in relatively lower *Lmna* and higher *Lmnc* compared to wild‐type cells. At the protein level, we noted increased levels of A‐type lamins at later stages of differentiation when cultures were less proliferative and had acquired a matrix‐producing, fully differentiated phenotype (Figure 4b). Additional confirmation of progerin accumulation in these cells was provided by immunofluorescent microscopy, which revealed punctate cytoplasmic aggregates and nuclear localization of progerin as well as the presence of multiple nuclei and micronuclei in homozygous osteoblasts (Figure 4c). Such nuclear abnormalities are a hallmark of HGPS cells expressing progerin.

**FIGURE 5:**
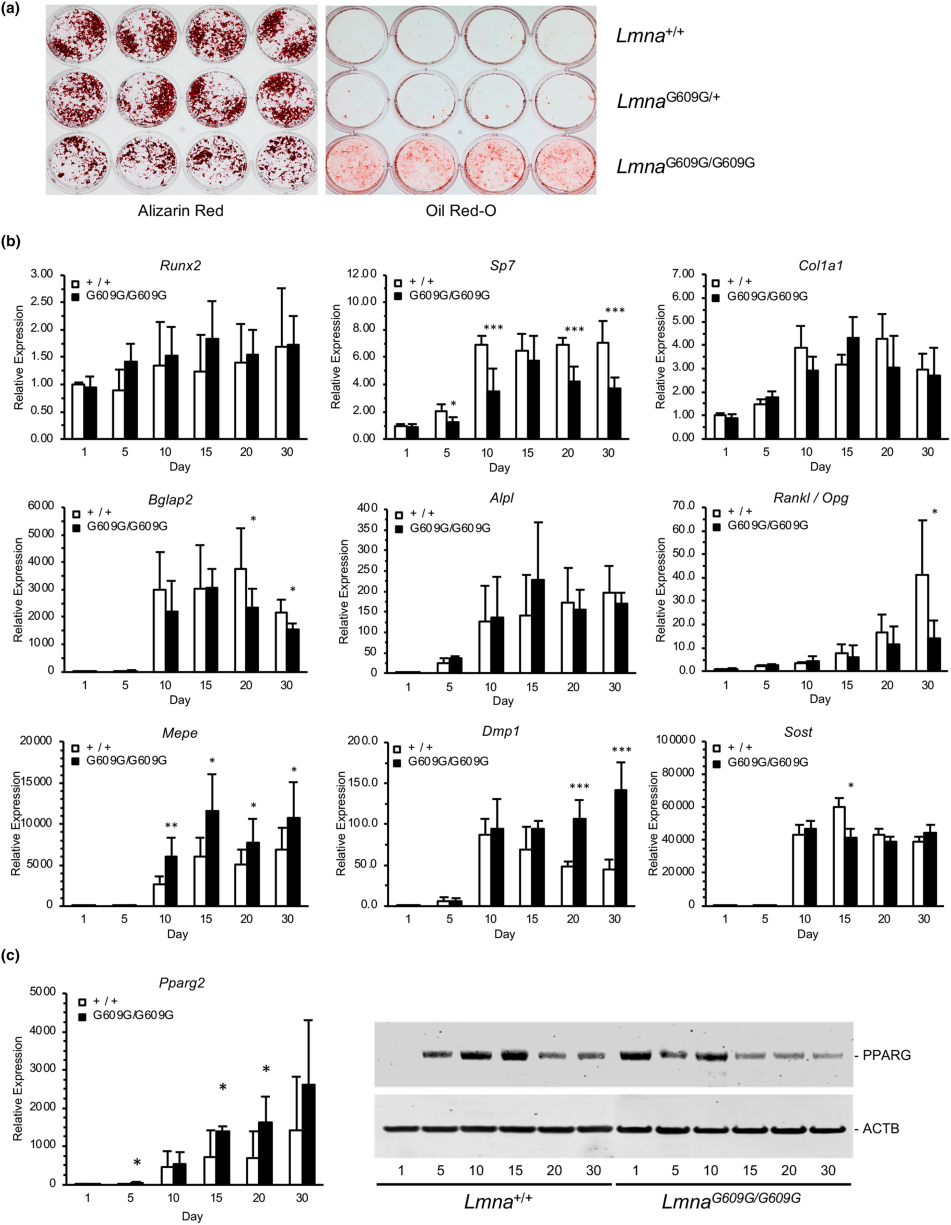
Increased phenotypic plasticity in *Lmna* ^G609G/G609G^ osteoblasts. (a) Left, newborn calvarial pre‐osteoblasts isolated from homozygous (*Lmna* ^G609G/G609G^) mice exhibit reduced capacity to deposit and mineralize matrix compared to wild‐type (*Lmna* ^+/+^) and heterozygous (*Lmna* ^G609G/+^) cultures. Cells were maintained in osteogenic media for 3 weeks followed by staining with alizarin red. Right, homozygous cultures accumulate lipids, detected by oil red‐O staining, when cultured in adipogenic media for 2 weeks. (b) Quantitative RT‐PCR of transcripts from differentiating *Lmna* ^G609G/G609G^ osteoblasts shows dysregulated expression of osteogenic markers required for differentiation, cellular crosstalk, and matrix mineralization. Results are the averages of three independent experiments. (c) *Lmna* ^G609G/G609G^ osteoblasts express higher levels of *Pparg2* transcripts, left, and PPARG protein at earlier stages of differentiation compared to wild‐type (*Lmna* ^+/+^) cells, right. *p < 0.05; **p < 0.01; ***p < 0.001.

Analysis of *Lmna* ^G609G/G609G^ osteoblasts in vitro demonstrated their reduced capacity for osteogenic potential relative to wild‐type cells. Following 30 days of culture in osteogenic media, homozygous cells deposited 30 ± 2% less hydroxyapatite mineral compared to wild‐type cells (*p* < 0.001) (Figure 5a). No significant difference was detected for heterozygous cell cultures. Quantitation of osteogenic transcripts revealed no differences in expression levels of *Runx2*, *Col1a1*, or *Alpl*, but significantly lower levels of *Sp7/Osx* (*p* < 0.001) and osteocalcin (*p* < 0.05), and upregulation of the late markers of osteogenic differentiation, *Mepe* (*p* < 0.05) and *Dmp1* (*p* < 0.001), in homozygous relative to wild‐type cells after 30 days of culture in differentiation media (Figure 5b). Notably, the ratio of *Rankl/Opg* transcripts was significantly reduced due to an increase in *Opg* expression (*p* < 0.05). The reduced osteogenic gene expression in *Lmna* ^G609G/G609G^ osteoblasts cultured in osteogenic media was further exemplified by lower levels of total intracellular active beta catenin compared to wild‐type cells (Figure S4), recapitulating the lower expression of WNT signaling pathway genes at the tissue level (Figure S3b). When cells were cultured in adipogenic media for 3 weeks, however, we observed a 14 ± 3% (*p* < 0.01) and 47 ± 6% (*p* < 0.001) increase in the accumulation of lipid in *Lmna* ^G609G/+^ and *Lmna* ^G609G/G609G^, respectively, compared to wild‐type (Figure 5a). Corresponding to the increased capacity to accumulate lipid upon adipogenic stimulation we found that, even when cultured in osteogenic medium, *Pparg2* transcription was upregulated and PPARG protein appeared earlier in *Lmna* ^G609G/G609G^ versus wild‐type osteoblasts (Figure 5c). These in vitro data support our findings that suggest, in vivo mesenchyme‐derived progerin‐expressing bone cell populations are diverted from an osteogenic to adipogenic phenotype.

**FIGURE 6:**
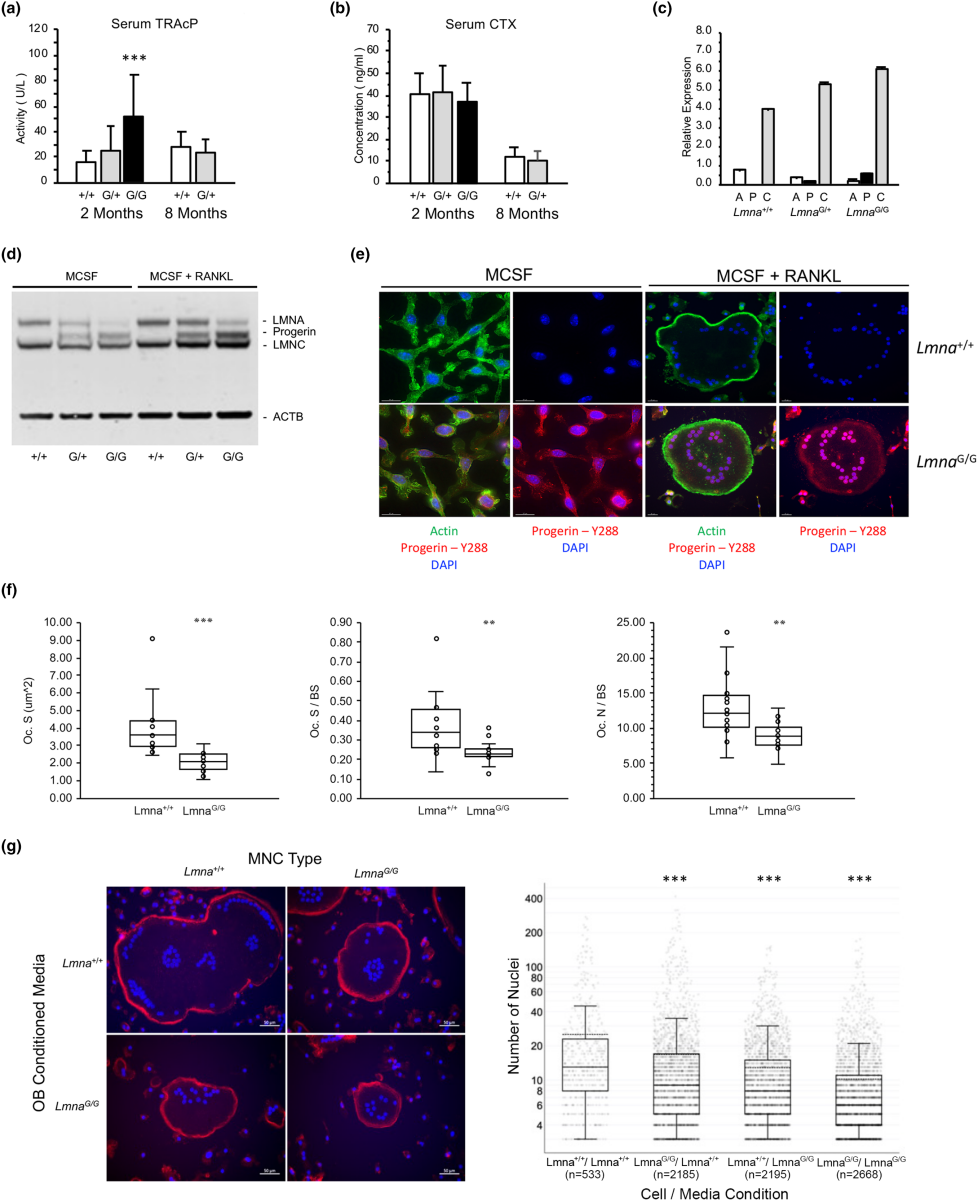
Osteoclastogenesis is affected by *Lmna* ^G609G/G609G^ osteoblast secreted factors. (a) Serum levels of TRAcP are increased in 2 month‐old homozygotes (G/G) compared to wild‐type (+/+) and heterozygous (G/+) mice. *N* = 12/genotype; 6 males, 6 females. *p < 0.05; **p < 0.01; ***p < 0.001 (b) Serum Type I collagen C‐telopeptide levels (CTX) are equivalent in 2 and 8 month‐old mice. *N* = 12/genotype; 6 males, 6 females. (c) Quantitation of *Lmna*‐derived transcripts in cultured marrow‐derived osteoclasts following treatment with MCSF and RANKL to induce fusion, including *Lmna* (A), *Progerin* (P), and *Lmnc* (C) transcripts. (d) Immunoblot of cultured mononuclear precursors (treated with MCSF) and mature, multinucleated osteoclast (treated with MCSF+RANKL) lysates demonstrates the presence of LMNA, Progerin, and LMNC. A‐type lamins are observed at greater levels in multinucleated osteoclasts compared to mononuclear precursors. (e) Wide‐field imaging of marrow‐derived mononuclear precursors (MCSF) and multinucleated osteoclasts (MCSF+RANKL) at 20× magnification. Cells were stained to visualize nuclei (DAPI, blue), Actin (green), and progerin (red). (f) Static histomorphometric analysis of osteoclasts on femoral trabecular surfaces of 8 week‐old wild‐type (Lmna^+/+^) and homozygous (Lmna^G/G^) mice demonstrates reduced osteoclast surface (Oc. S), osteoclast surface per bone surface (Oc. S/BS) and osteoclast number per bone surface (Oc. N/BS). *N* = 12 per genotype (six males, six females). (g) Immunofluorescent staining of cultured mature osteoclasts derived from wild‐type (*Lmna* ^+/+^) and homozygous (*Lmna* ^G/G^) 8‐week marrow progenitors in the presence of femoral osteoblast‐conditioned media. The number of nuclei present in osteoclasts cultured under each condition was obtained from two independent cultures. *N*, number of cells analyzed.

Since normal aging‐related bone loss is associated with dysregulation of remodeling due to decreased osteoblast bone deposition and increased osteoclast resorption, we next sought to characterize the role of hematopoietic cell lineages in progeroid mouse bone. Although serum TRAcP levels, indicative of osteoclast numbers were increased type I collagen C‐telopeptide levels (CTX), a marker of resorptive activity were normal in *Lmna* ^G609G^ mice (Figure 6a,b). To determine if intrinsic factors altered osteoclastogenesis, we next analyzed the in vitro differentiation of mononuclear precursors isolated from marrow of 2 month‐old mice. Quantitative RT‐PCR and western analyses confirmed the expression of A‐type lamins in marrow‐derived cultured mononuclear precursors and mature, multinucleated osteoclasts (Figure 6c,d). Interestingly, immunofluorescent staining identified the presence of progerin throughout the nuclear lamina and cytoplasm in *Lmna* ^G609G/G609G^ marrow‐derived mononuclear precursors. Following treatment with RANKL to induce fusion, the diffuse cytoplasmic staining pattern altered to overlap with the F‐actin ring underlying the plasma membrane and within the nuclear lamina (Figures 6e and S5a). We found, however, that induction of fusion of marrow precursors did not result in any difference in osteoclast formation, since cell surface area and average number of nuclei was equivalent among each genotype (Figure S5b).

To determine the status of bone resorption in HGPS mice static histomorphometric analysis of femoral trabecular surfaces in 8‐week mice was performed. Several indices of osteoclast activity were decreased including a 49 ± 13% reduction in total osteoclast surface (Oc. S, *p* = 0.0002) associated with a 32 ± 15% decrease in the number of osteoclasts residing on trabecular surfaces (Oc. N/BS, *p* = 0.005), as well as a 35 ± 14% decrease in osteoclast surface per bone surface (Oc. S/BS, *p* = 0.009) (Figure 6f). The inconsistency between our in vitro and in vivo osteoclast data prompted us to repeat the osteoclast formation assays under more physiologic conditions. When cultured in conditioned media generated by *Lmna* ^G609G/G609G^ femoral osteoblasts, both wild‐type and HGPS marrow‐derived mononuclear precursors developed into osteoclasts containing an average 49% (*p* = 1.14 × 10^13^) and 40% (*p* = 3.25 × 10^−14^) fewer nuclei, respectively, than when they were cultured in wild‐type femoral osteoblast‐conditioned media (Figure 6g). Furthermore, we observed a 33% reduction in the number of *Lmna* ^G609G/G609G^ osteoclast nuclei versus wild‐type cells when cultured in wild‐type derived osteoblast media (*p* = 1.00 × 10^−6^). Subsequent cytokine profiling of conditioned media revealed 53 inflammation‐associated factors that were significantly altered in *Lmna* ^G609G/G609G^‐derived media compared to wild‐type cultures including osteoprotegerin (OPG/TNFRSF11B), a well‐characterized decoy receptor that prevents the RANK‐RANKL interaction required for the formation of osteoclasts from their precursors (Table S4). Similar to *Lmna* ^G609G/G609G^ plasma, increased levels of CXCL16, PTX3, DCN, CST3, IGFBP5, and MMP9 were identified in *Lmna* ^G609G/G609G^ conditioned media, in addition to other metalloproteases, CCL and CXCL chemokines, interleukins and IGF/FGF signaling components. We therefore conclude that reduced recruitment and activity of osteoclasts in *Lmna* ^G609G/G609G^ bone is a response to unidentified intrinsic defects, as well as extrinsic local factors presumably secreted by osteoblast lineage cells.

## 3 DISCUSSION

Although mortality in HGPS is generally due to myocardial infarction or stroke as a result of rapidly progressive atherosclerosis, these patients also exhibit alopecia, bone and joint abnormalities, and subcutaneous fat loss. Thus, cells and tissues derived from common mesenchymal progenitors are particularly susceptible to pathology resulting from the accumulation of progerin. In this study we have expanded on previous characterizations of the *Lmna* ^G609G^ murine bone phenotype involving development of low bone mineral density (BMD), kyphosis, micrognathia, dental, and other craniofacial abnormalities that worsen in an age‐ and genotype‐dependent manner (Osorio et al., 2011; Zaghini et al., 2020). To further define pathomechanisms underlying HGPS‐associated bone loss at the tissue level, we investigated the molecular and cellular alterations in *Lmna* ^G609G^ bone cell populations, which lead to defective tissue modeling and progressive bone dysplasia. The alterations in cellular differentiation and extracellular matrix production result in a pattern of decreased, poorly mineralized, extracellular matrix. In addition, we have identified several inflammatory cytokines that may act locally or systemically to modulate the severity of the HGPS phenotype. The inflammation and cellular phenotypic plasticity that occurs in HGPS bone tissue in this knock‐in mouse model might also elucidate the mechanisms of vascular and adipose pathology in patients, potentially providing additional therapeutic approaches to treatment of HGPS.

In humans, aging‐associated trabecular bone loss begins in young adults, with decreases in BMD being greater in women than men. Gender‐specific differences in bone structural alterations were also identified in *Lmna* ^G609G^ mice. First, in contrast to *Lmna* ^G609G/G609G^ mice, *Lmna* ^G609G/+^ mice demonstrated relatively few reductions in bone structural parameters at 2 months of age. Trabecular and cortical BMD was decreased in male and female 2‐month *Lmna* ^G609G/G609G^ mice, but in females only among the 8‐month *Lmna* ^G609G/+^ mice. In 2‐month homozygotes trabecular bone loss was most severe in males as demonstrated by the more significant differences in bone volume (Tb BV) and mineral density (Tb BMD). For both genotypes the trend toward increased structural model indices (SMI) represented a shift from a plate to rod‐like geometry as occurs in osteoporosis (Akhter et al., 2007). By 8 months trabecular bone loss in heterozygous mice was greatest in females, while reduced cortical parameters that were absent at 2 months had progressed in both genders.

Aging‐related cortical bone loss in humans begins following midlife, is accompanied by greater increases in endocortical versus periosteal area and can be explained by an imbalance in bone resorption and apposition (Corrado et al., 2020). Conversely, structural analyses of *Lmna* ^G609G/+^ femoral diaphyses revealed that the reduced cross‐sectional thickness of cortical bone occurred without a concurrent increase in marrow area implying normal endocortical resorption with decreased periosteal bone deposition. In contrast to heterozygous mice, where cortical thinning was progressive from 2 to 8 months of age, homozygous femoral cortical thickness was already significantly reduced compared to wild‐type in a chronologic and gene dosage‐specific manner despite the lack of significantly reduced bone formation rates at this age. A possible explanation for this discrepancy is that rapid bone loss in *Lmna* ^G609G/G609G^ mice occurs prior to early adulthood (i.e., 2 months of age) when dynamic histomorphologic analyses were performed, in contrast to normal aging associated bone loss. Considering the subtle skeletal alterations in newborn *Lmna* ^G609G/+^ and *Lmna* ^G609G/G609G^ mice, these findings indicate that both heterozygous and homozygous mice develop progressively reduced BMD and bone structural parameters with some features that are distinct from aging‐related osteoporosis.

The altered structural parameters predicted increased fragility of 2 month‐old *Lmna* ^G609G/G609G^, but not 8 month‐old *Lmna* ^G609G/+^, femora as demonstrated by the significantly reduced MOI. Subsequent mechanical testing demonstrated reductions in both elastic and plastic properties of cortical bone that reflect the smaller size. The reduced load and work required to yield implied alterations in the inorganic component of bone tissue, consistent with the reduced BMD in these mice. Although the fracture load of *Lmna* ^G609G/G609G^ femora was equivalent to wild‐type littermates, the reduced post‐yield work required to fracture suggested additional alterations to the organic contents of the tissue. This altered plastic property of *Lmna* ^G609G^ femora may result from the abnormal collagen cross‐link profile but may also reflect changes in the proteoglycan composition of the extracellular matrix that were suggested by the negative enrichment scores found among the KEGG proteoglycan synthesis pathways. Importantly, reduced MOI with normal stiffness may indicate alterations in bone material properties in homozygous mice that cannot be accounted for by differences in geometry or mineral content.

During normal aging vascular stiffening, particularly of the aorta, is in part associated with an increase in the extent of collagen cross‐linking due to a greater abundance of pentosidine, one of the well‐characterized advanced glycation end‐products (AGEs) (Hoshino et al., 1995). In HGPS arterial stiffening may proceed by alternative mechanisms. As demonstrated in the *Lmna* ^G609G^ mouse model this is initiated by the upregulation of lysyl oxidase (LOX) (von Kleeck et al., 2021), the enzyme that catalyzes oxidative deamination of telopeptidyl lysine (Lys) and hydroxylysine (Hyl) residues to initiate formation of covalent cross‐links between collagen molecules. Consistent with these observations we have found that even with normal composition in vivo, bone collagen contained significantly increased numbers of aldehydes involved in cross‐linking in both heterozygous and homozygous mice implying a global mechanism for increased LOX activity in HGPS. Importantly, the increase in reducible cross‐links and decreased BMD of *Lmna* ^G609G/G609G^ bone tissue is strikingly similar to the alterations in bone material properties that occur in immobilization‐associated osteoporosis which may reflect dysregulation of mechanotransduction in bone cell populations (Yamauchi et al., 1988).

Neither the role of A‐type lamins in osteoclast differentiation nor the contribution of pathologic bone resorption to the HGPS skeletal phenotype has been fully clarified. In an inducible bone‐specific transgenic mouse model that expresses the most common HGPS mutation, femoral expression levels of the osteoclast markers *Mmp9* and *Ctsk* were similar to wild‐type mice (Schmidt et al., 2012). Contrary to these findings, accumulation of unprocessed prelamin A in *Zmpste24* ^−/−^ mice is associated with fewer osteoclasts and decreased bone resorption in vivo, as determined by histologic analysis of TRAcP activity and serum Ctx levels (Rivas et al., 2009). In vitro, however, chemical induction of prelamin A accumulation in differentiating peripheral blood mononuclear cells leads to accelerated formation of mature osteoclasts containing greater numbers of nuclei, but with reduced resorptive activity (Zini et al., 2008). Among LMNA deficiency models, *Lmna* ^−/−^ mice show a marked reduction in osteoclast numbers with a corresponding decrease in serum markers of bone turnover, yet in co‐culture studies knockdown of *LMNA* in bone marrow stromal cells enhanced the fusion of mononuclear precursors in a RANKL‐dependent manner (Li et al., 2011; Rauner et al., 2009). Interestingly tissue‐specific knockout studies have suggested that loss of *Lmna* expression in skeletal muscle, but not bone, drives increased resorption that can be rescued by genetic ablation of the pro‐osteoclastogenic cytokine IL6 (Xiong et al., 2020). Furthermore, antibody‐based inhibition of IL6 improved some bone structural parameters in *Lmna* ^G609G/G609G^ mice, implying an inflammatory aspect to bone loss in these mice (Squarzoni et al., 2021). Given our histologic, biochemical, and gene expression data we hypothesize a role of increased hematopoietic precursor recruitment driven by elevated systemic levels of inflammatory cytokines in *Lmna* ^G609G/G609G^ mice, but that osteoclastogenesis is inhibited by local factors including osteoblast secreted OPG. Furthermore, the normal CTX levels observed in serum and the normal levels of osteoclast marker gene expression found in femoral bone tissue, despite fewer TRAcP‐positive cells on bone surfaces, suggests increased resorptive activity by progerin‐expressing osteoclasts per se. Thus, one can speculate that progressive osteolysis, such as occurs in the phalanges and clavicles of HGPS patients may develop due to increased resorption that is not countered by the competing activity of osteoclast‐suppressing factors in specific bone compartments.

Loss of adipose tissue is among the principal pathologies in HGPS patients and mouse models (Cabral et al., 2021; Gordon et al., 2007; Osorio et al., 2011; Pendás et al., 2002; Yang et al., 2006). At the tissue level, it is thought that the lipoatrophy that occurs in HGPS is associated with chronic inflammation and concurrent development of the senescence‐associated secretory phenotype (SASP) in adipocyte progenitors that accumulate progerin (Najdi et al., 2021). At the molecular level, loss of principal fat deposits has been attributed to sequestration of SREBP1 by progerin resulting in the inhibition of late‐stage gene induction (PPARγ2 and C/EBPα) in differentiating mesenchymal stem cells (MSCs) (Maraldi et al., 2008; Xiong et al., 2013). We were therefore surprised to find a transcriptional signature in femoral bone tissue from *Lmna* ^G609G/G609G^ mice that reflects enrichment of pathways associated with adipogenic differentiation (Zhang et al., 2018). While we cannot rule out the possibility that upregulation of adipogenic gene expression in femoral tissue represents infiltration of adipocytes from the bone marrow compartment, accumulation of lipid and elevated expression levels of *Pparg2* in vitro support a role of intrinsic factors, such as epigenetic alterations, and extrinsic factors, such as inflammatory cytokines, involved in phenotypic plasticity of *Lmna* ^G609G/G609G^ osteoblasts.

Although gene expression patterns in femoral tissue represent a mixed cell population of osteoblasts, osteocytes and osteoclasts, GSEA revealed several additional pathways critical for bone homeostasis that serve as potential targets for treatment strategies. Specifically, our data point to altered activity at the plasma membrane of *Lmna* ^G609G/G609G^ bone cell populations as a contributing factor in osteoblast dysfunction. For example, ours is not the first study to demonstrate reduced WNT signaling in cells or tissues expressing progerin, characterized by decreased intracellular nuclear levels of active beta catenin and lower expression of downstream beta catenin target genes (Hernandez et al., 2010; Schmidt et al., 2012; Sola‐Carvajal et al., 2019). In *Zmpste24* ^−/−^ osteoblasts the reduced WNT signaling is associated with cytoplasmic accumulation of beta catenin due to defective nuclear translocation (Choi et al., 2018). In *Lmna* ^G609G/G609G^ osteoblasts, however, we found reduced expression of multiple ligands, receptors and cytoplasmic components that mediate WNT signaling, but no evidence of cytoplasmic accumulation of beta catenin, suggesting that WNT signaling is inhibited at either the receptor level or among cytoplasmic components of the pathway. In addition, the finding that extracellular matrix‐receptor interactions, focal adhesions and adherens junctions signaling pathways were downregulated point to dysregulation of cell–cell and cell‐matrix interactions required for WNT crosstalk, mechanosensing, differentiation and function of cells of the osteoblast and osteoclast lineages and, therefore, skeletal development and bone remodeling (Marie et al., 2014).

Our goals in the current study were to determine the extent to which HGPS bone pathology resembles senile osteoporosis and to further characterize pathomechanisms underlying the bone phenotype. We found that progressive bone loss in *Lmna* ^G609G/G609G^ mice occurs in the context of increased marrow adiposity associated with systemic inflammation and decreased bone formation that represents a low bone turnover phenotype, which does not recapitulate the altered bone remodeling that occurs with normal aging. Although progerin expression has been found in aged tissues of apparently healthy individuals, the potential for its role in the onset of senile primary osteoporosis remains to be determined (McClintock et al., 2007; Scaffidi & Misteli, 2006). In the context of HGPS, targeting the specific pathways identified here may ameliorate, but will not prevent, the pathology caused by the dominant negative function of progerin. Indeed, more effective therapies will require complex approaches that target and eliminate progerin at the protein, RNA and DNA levels, strategies that are currently in development (Cabral et al., 2021; Erdos et al., 2021; Koblan et al., 2021).

## 4 EXPERIMENTAL PROCEDURES

Experimental procedures and materials used in this study can be found in the Supplemental files accompanying this article.

## AUTHOR CONTRIBUTIONS

WAC, SMW, MY, KMK, MRE and FSC designed experiments, which were conducted by WAC, CS, AAT, OB, MT, and ULT. Data was analyzed by WAC, CS, AAT, OB, MT, NN, TY and MRE. Manuscript was written by WAC, MRE, and FSC, with significant input from MY and KMK.

## CONFLICT OF INTEREST STATEMENT

None declared.

## Supporting information


Data File S1.

Data File S2.

Appendix S1.

## Data Availability

RNA sequence reads and gene quantification data are available at GEO with accession number GSE231305. All other data that support the findings of this study are available from the authors upon request.
