# Supplementary material for: Bone dysplasia in Hutchinson‐Gilford progeria syndrome is associated with dysregulated differentiation and function of bone cell populations
Source: Aging Cell. 2023 Jun 26;22(9):e13903. doi: 10.1111/acel.13903 (PMC10497813; doi:10.1111/acel.13903)
Supplement: Supplementary file 1 — Data File S1. [file ACEL-22-e13903-s002.pdf]

Supplemental Data File 1. KEGG pathway analysis of femoral tissue gene expression.

pathway	pval	padj	log2err	ES	NES
KEGG_FATTY_ACID_METABOLISM	2.63E-07	4.84E-05	0.67496286	0.6595835	2.32031257
KEGG_DRUG_METABOLISM_CYTOCHROME_P450	9.40E-06	0.00034604	0.59332548	0.58583174	2.13531027
KEGG_CELL_CYCLE	7.11E-06	0.00034604	0.61052688	-0.4044785	-1.951767
KEGG_ECM_RECEPTOR_INTERACTION	3.83E-06	0.00034604	0.61052688	-0.4657382	-2.0918664
KEGG_N_GLYCAN_BIOSYNTHESIS	7.58E-06	0.00034604	0.61052688	-0.5527208	-2.1834635
KEGG_PPAR_SIGNALING_PATHWAY	2.21E-05	0.00065749	0.57561026	0.54580806	2.08372259
KEGG_LYSOSOME	3.22E-05	0.00065749	0.55733224	-0.3811441	-1.8266412
KEGG_HEDGEHOG_SIGNALING_PATHWAY	2.95E-05	0.00065749	0.57561026	-0.526912	-2.0884944
KEGG_DNA_REPLICATION	3.08E-05	0.00065749	0.55733224	-0.5871246	-2.2329943
KEGG_SPLICEOSOME	3.59E-05	0.00066146	0.55733224	-0.3871367	-1.8498553
KEGG_AXON_GUIDANCE	4.78E-05	0.00079913	0.55733224	-0.379236	-1.8321388
KEGG_PEROXISOME	6.17E-05	0.00091632	0.5384341	0.48486632	1.98262825
KEGG_FOCAL_ADHESION	6.47E-05	0.00091632	0.5384341	-0.3249408	-1.6819278
KEGG_CYTOKINE_CYTOKINE_RECEPTOR_INTERACTION	9.10E-05	0.001116	0.5384341	0.35555822	1.69991528
KEGG_VIBRIO_CHOLERAE_INFECTION	9.00E-05	0.001116	0.5384341	-0.4890888	-1.9643278
KEGG_CITRATE_CYCLE_TCA_CYCLE	0.00012575	0.00144608	0.51884808	0.60563716	2.03531666
KEGG_METABOLISM_OF_XENOBIOTICS_BY_CYTOCHROME_P450	0.00014933	0.00161626	0.51884808	0.54775108	1.96602798
KEGG_BASAL_CELL_CARCINOMA	0.0002882	0.00294607	0.49849311	-0.4848361	-1.9217203
KEGG_WNT_SIGNALING_PATHWAY	0.00031329	0.00303397	0.49849311	-0.3458466	-1.7145654
KEGG_VALINE_LEUCINE_AND_ISOLEUCINE_DEGRADATION	0.00044514	0.00409531	0.49849311	0.5242593	1.91088359
KEGG_RETINOL_METABOLISM	0.0005368	0.00470339	0.47727082	0.55717419	1.90348818
KEGG_STARCH_AND_SUCROSE_METABOLISM	0.00081166	0.00678845	0.47727082	0.53901306	1.85321048
KEGG_HEMATOPOIETIC_CELL_LINEAGE	0.00101165	0.00775596	0.45505987	0.42293259	1.7331933
KEGG_GLYCOSAMINOGLYCAN_BIOSYNTHESIS_CHONDROITIN_SU	0.00101113	0.00775596	0.45505987	-0.6043942	-2.0329264
KEGG_MELANOGENESIS	0.00213399	0.01570618	0.4317077	-0.3610646	-1.6631907
KEGG_CIRCADIAN_RHYTHM_MAMMAL	0.00298903	0.02071304	0.4317077	0.68293872	1.82464938
KEGG_GLYCOSAMINOGLYCAN_BIOSYNTHESIS_KERATAN_SULFAT	0.00303941	0.02071304	0.4317077	-0.6435083	-1.887953
KEGG_ADHERENS_JUNCTION	0.00390514	0.02566238	0.4317077	-0.364705	-1.5941233
KEGG_PATHWAYS_IN_CANCER	0.00490579	0.03112636	0.40701792	-0.2471515	-1.3529389
KEGG_GLIOMA	0.00507784	0.03114408	0.40701792	-0.3705179	-1.5812069
KEGG_GLYCOLYSIS_GLUCONEOGENESIS	0.00617011	0.03662258	0.40701792	0.41146489	1.5920583
KEGG_PROPANOATE_METABOLISM	0.00811046	0.0454282	0.3807304	0.50469919	1.71298213
KEGG_NITROGEN_METABOLISM	0.00814745	0.0454282	0.3807304	0.54879993	1.7019507
KEGG_SULFUR_METABOLISM	0.01055657	0.05712969	0.3807304	0.66201721	1.72426077
KEGG_T_CELL_RECEPTOR_SIGNALING_PATHWAY	0.01143224	0.06010092	0.3807304	0.34810519	1.50195553
KEGG_UBIQUITIN_MEDIATED_PROTEOLYSIS	0.01220133	0.06236237	0.3807304	-0.2981427	-1.4495483
KEGG_ADIPOCYTOKINE_SIGNALING_PATHWAY	0.01302157	0.06475592	0.3807304	0.38991598	1.5357968
KEGG_FRUCTOSE_AND_MANNOSE_METABOLISM	0.0140297	0.06619141	0.3807304	0.45324234	1.56531339
KEGG_PROSTATE_CANCER	0.01400373	0.06619141	0.3807304	-0.3279679	-1.486898
KEGG_ALANINE_ASPARTATE_AND_GLUTAMATE_METABOLISM	0.01576417	0.07167069	0.35248786	0.49893378	1.66884794
KEGG_VIRAL_MYOCARDITIS	0.0159701	0.07167069	0.35248786	0.386711	1.51762318
KEGG_PATHOGENIC_ESCHERICHIA_COLI_INFECTION	0.01794278	0.07860647	0.35248786	-0.3771378	-1.514699
KEGG_BASE_EXCISION_REPAIR	0.01900193	0.0813106	0.35248786	-0.4158166	-1.5487195
KEGG_VASOPRESSIN_REGULATED_WATER_REABSORPTION	0.02003781	0.08379449	0.35248786	-0.410664	-1.5991384
KEGG_MELANOMA	0.0223255	0.08930201	0.35248786	-0.3342643	-1.417136
KEGG_PROTEIN_EXPORT	0.02207861	0.08930201	0.35248786	-0.4645887	-1.5909589
KEGG_TRYPTOPHAN_METABOLISM	0.02516779	0.0985292	0.37603931	0.43823767	1.50672908
KEGG_FC_GAMMA_R_MEDIATED_PHAGOCYTOSIS	0.02636775	0.09942574	0.35248786	-0.3065853	-1.4130145
KEGG_HOMOLOGOUS_RECOMBINATION	0.02647751	0.09942574	0.35248786	-0.4380698	-1.5560113
KEGG_GAP_JUNCTION	0.02892686	0.10645086	0.35248786	-0.3156914	-1.4069986
KEGG_MISMATCH_REPAIR	0.03002723	0.10833354	0.35248786	-0.4737524	-1.613887
KEGG_ARGININE_AND_PROLINE_METABOLISM	0.03074851	0.10880243	0.35248786	-0.3625208	-1.4477295
KEGG_NON_HOMOLOGOUS_END_JOINING	0.03546099	0.12310986	0.37603931	-0.5365214	-1.5487458
KEGG_HYPERTROPHIC_CARDIOMYOPATHY_HCM	0.03738318	0.12737972	0.29406667	0.34383699	1.41870337

KEGG_TYROSINE_METABOLISM	0.04026846	0.13471629	0.29406667	0.42191173	1.45059795
KEGG_GLYCOSPHINGOLIPID_BIOSYNTHESIS_GANGLIO_SERIES	0.04411765	0.14495798	0.34179338	-0.5067811	-1.5240771
KEGG_NOD_LIKE_RECEPTOR_SIGNALING_PATHWAY	0.0544	0.17560702	0.24504179	0.35700671	1.39272161
KEGG_CHRONIC_MYELOID_LEUKEMIA	0.05617978	0.17822549	0.32347051	-0.30167	-1.3185978
KEGG_B_CELL_RECEPTOR_SIGNALING_PATHWAY	0.05915493	0.18448317	0.31532483	-0.3005614	-1.3145245
KEGG_LEUKOCYTE_TRANSENDOTHELIAL_MIGRATION	0.06156156	0.18878879	0.22205605	0.30459969	1.30992821
KEGG_NON_SMALL_CELL_LUNG_CANCER	0.0656168	0.19792608	0.28785712	-0.3273578	-1.3364482
KEGG_CELL_ADHESION_MOLECULES_CAMS	0.07216495	0.21076747	0.20207171	0.29639952	1.29645228
KEGG_ENDOCYTOSIS	0.07158095	0.21076747	0.28780513	-0.245378	-1.2513357
KEGG_NATURAL_KILLER_CELL_MEDIATED_CYTOTOXICITY	0.0739645	0.21264793	0.19991523	0.29649839	1.28691126
KEGG_AMINO_SUGAR_AND_NUCLEOTIDE_SUGAR_METABOLISM	0.07591623	0.21490133	0.26635066	-0.35298	-1.379607
KEGG_GRAFT_VERSUS_HOST_DISEASE	0.08823529	0.23529412	0.19189224	0.41669416	1.37851088
KEGG_PROTEASOME	0.08709677	0.23529412	0.19189224	0.37045416	1.35027604
KEGG_INTESTINAL_IMMUNE_NETWORK_FOR_IGA_PRODUCTION	0.08482871	0.23529412	0.195789	0.37215318	1.34837885
KEGG_GLYCOSAMINOGLYCAN_DEGRADATION	0.08665105	0.23529412	0.23439265	-0.4296587	-1.4083428
KEGG_PENTOSE_PHOSPHATE_PATHWAY	0.09313725	0.24136979	0.18643256	0.41269201	1.36527097
KEGG_BUTANOATE_METABOLISM	0.09271523	0.24136979	0.18820415	0.39982297	1.34365325
KEGG_NEUROACTIVE_LIGAND_RECEPTOR_INTERACTION	0.09570042	0.24456773	0.16823817	0.26053291	1.22551061
KEGG_TIGHT_JUNCTION	0.1037037	0.26139016	0.16693385	0.28129042	1.24029774
KEGG_PROGESTERONE_MEDIATED_OOCYTE_MATURATION	0.11111111	0.27627628	0.22496609	-0.2790735	-1.2472795
KEGG_PRION_DISEASES	0.1175	0.28826667	0.20658792	-0.3395795	-1.2570099
KEGG_ABC_TRANSPORTERS	0.13175676	0.3068765	0.1574029	0.35507345	1.24909339
KEGG_PYRIMIDINE_METABOLISM	0.1300578	0.3068765	0.21140019	-0.2640138	-1.2100951
KEGG_O_GLYCAN_BIOSYNTHESIS	0.1308642	0.3068765	0.1938133	-0.37262	-1.2872244
KEGG_OTHER_GLYCAN_DEGRADATION	0.12990196	0.3068765	0.1938133	-0.4415413	-1.327877
KEGG_GLUTATHIONE_METABOLISM	0.13870968	0.31903226	0.14920754	0.34106614	1.24315905
KEGG_OOCYTE_MEIOSIS	0.14285714	0.32451499	0.20207171	-0.2509762	-1.1840793
KEGG_ANTIGEN_PROCESSING_AND_PRESENTATION	0.14975845	0.32561036	0.14290115	0.32613378	1.24507564
KEGG_TYPE_I_DIABETES_MELLITUS	0.14950166	0.32561036	0.14551615	0.3639827	1.24348323
KEGG_SMALL_CELL_LUNG_CANCER	0.15041783	0.32561036	0.19189224	-0.2646692	-1.1887638
KEGG_SNARE_INTERACTIONS_IN_VESICULAR_TRANSPORT	0.14563107	0.32561036	0.18138313	-0.327866	-1.2326378
KEGG_TGF_BETA_SIGNALING_PATHWAY	0.15277778	0.32687339	0.19002331	-0.2669102	-1.192917
KEGG_NUCLEOTIDE_EXCISION_REPAIR	0.16230366	0.34326292	0.17821987	-0.3066982	-1.1987165
KEGG_DILATED_CARDIOMYOPATHY	0.16434109	0.34362227	0.1328463	0.28458626	1.18382205
KEGG_REGULATION_OF_ACTIN_CYTOSKELETON	0.17164179	0.35485494	0.20895503	-0.2151314	-1.1164915
KEGG_TERPENOID_BACKBONE_BIOSYNTHESIS	0.17857143	0.36507937	0.13355495	0.46365718	1.26822066
KEGG_OLFACTORY_TRANSDUCTION	0.19261637	0.38946607	0.12384217	0.32322343	1.18490002
KEGG_VALINE_LEUCINE_AND_ISOLEUCINE_BIOSYNTHESIS	0.1982906	0.3965812	0.12625399	0.51877656	1.2843732
KEGG_LEISHMANIA_INFECTION	0.20450886	0.39930157	0.11988785	0.29229337	1.16342246
KEGG_COLORECTAL_CANCER	0.20540541	0.39930157	0.1596467	-0.2784972	-1.1606064
KEGG_DORSO_VENTRAL_AXIS_FORMATION	0.20616114	0.39930157	0.1482615	-0.3546613	-1.1929305
KEGG_ENDOMETRIAL_CANCER	0.2109375	0.40040258	0.15419097	-0.2843814	-1.153533
KEGG_TYPE_II_DIABETES_MELLITUS	0.21108179	0.40040258	0.15524197	-0.2957526	-1.1577316
KEGG_PENTOSE_AND_GLUCURONATE_INTERCONVERSIONS	0.22053872	0.4140727	0.11776579	0.43829032	1.22276564
KEGG_RIBOSOME	0.23565891	0.43799233	0.10797236	0.27116927	1.12801002
KEGG_NICOTINATE_AND_NICOTINAMIDE_METABOLISM	0.24449878	0.44987775	0.13725078	-0.3347777	-1.1464283
KEGG_TASTE_TRANSDUCTION	0.25679012	0.46781567	0.13427345	-0.3305218	-1.1417952
KEGG_VASCULAR_SMOOTH_MUSCLE_CONTRACTION	0.25947522	0.46807294	0.14641624	-0.2334585	-1.1014327
KEGG_AUTOIMMUNE_THYROID_DISEASE	0.26633987	0.47579161	0.10357633	0.34913205	1.15500139
KEGG_ERBB_SIGNALING_PATHWAY	0.27808989	0.49200519	0.13802224	-0.2438702	-1.0903842
KEGG_GNRH_SIGNALING_PATHWAY	0.28205128	0.49426129	0.13802224	-0.2365597	-1.0718184
KEGG_GLYCEROLIPID_METABOLISM	0.29032258	0.50395618	0.09754492	0.30237146	1.10212002
KEGG_BETA_ALANINE_METABOLISM	0.30155979	0.50796767	0.09957912	0.37908542	1.14540555
KEGG_STEROID_HORMONE_BIOSYNTHESIS	0.31195841	0.50796767	0.09754492	0.37593208	1.13587775
KEGG_CARDIAC_MUSCLE_CONTRACTION	0.30173776	0.50796767	0.09405035	0.2737976	1.098996
KEGG_PARKINSONS_DISEASE	0.29749632	0.50796767	0.09082414	0.25120145	1.09582717

KEGG_SYSTEMIC_LUPUS_ERYTHEMATOSUS	0.31007752	0.50796767	0.09139243	0.25975424	1.0805258
KEGG_ETHER_LIPID_METABOLISM	0.30848329	0.50796767	0.12384217	-0.3084649	-1.0956586
KEGG_GLYCOSAMINOGLYCAN_BIOSYNTHESIS_HEPARAN_SULFAT	0.3064133	0.50796767	0.11881504	-0.3242232	-1.1045002
KEGG_EPITHELIAL_CELL_SIGNALING_IN_HELICOBACTER_PYLORI_I	0.33245383	0.53659214	0.12043337	-0.2465024	-1.0519636
KEGG_RENAL_CELL_CARCINOMA	0.33604336	0.53766938	0.12154328	-0.2456522	-1.0556169
KEGG_GALACTOSE_METABOLISM	0.35452323	0.56234719	0.11101149	-0.3091558	-1.0586872
KEGG_HISTIDINE_METABOLISM	0.3692053	0.58063055	0.08528847	0.31864455	1.0708434
KEGG_SPHINGOLIPID_METABOLISM	0.37878788	0.59065229	0.08479851	0.30221111	1.05727178
KEGG_PANCREATIC_CANCER	0.39393939	0.60882353	0.11191832	-0.2360041	-1.0207226
KEGG_PANTOTHENATE_AND_COA_BIOSYNTHESIS	0.39705882	0.60882353	0.10395847	-0.3469162	-1.0433048
KEGG_PYRUVATE_METABOLISM	0.40703518	0.61896258	0.08063885	0.29006404	1.02970955
KEGG_LYSINE_DEGRADATION	0.4120603	0.621468	0.10319747	-0.2623408	-1.0180051
KEGG_CHEMOKINE_SIGNALING_PATHWAY	0.42977528	0.63042261	0.06879431	0.21762119	1.01652758
KEGG_COMPLEMENT_AND_COAGULATION_CASCADES	0.43870968	0.63042261	0.07473852	0.26166981	1.00312408
KEGG_ALLOGRAFT_REJECTION	0.44535073	0.63042261	0.07455008	0.29951736	1.00183421
KEGG_BIOSYNTHESIS_OF_UNSATURATED_FATTY_ACIDS	0.44540728	0.63042261	0.07767986	0.33143223	1.00142156
KEGG_PHOSPHATIDYLINOSITOL_SIGNALING_SYSTEM	0.44257703	0.63042261	0.10552094	-0.228273	-1.0021759
KEGG_LONG_TERM_POTENTIATION	0.42744063	0.63042261	0.10395847	-0.2368269	-1.0040434
KEGG_P53_SIGNALING_PATHWAY	0.43307087	0.63042261	0.10282184	-0.2353057	-1.006729
KEGG_ONE_CARBON_POOL_BY_FOLATE	0.43095238	0.63042261	0.09721508	-0.3299919	-1.022498
KEGG_FC_EPSILON_RI_SIGNALING_PATHWAY	0.47107438	0.65664914	0.10063339	-0.2260267	-0.9775702
KEGG_ALDOSTERONE_REGULATED_SODIUM_REABSORPTION	0.46813725	0.65664914	0.09405035	-0.2605992	-0.9925604
KEGG_DRUG_METABOLISM_OTHER_ENZYMES	0.485	0.66637401	0.09314546	-0.266668	-0.9871159
KEGG_LINOLEIC_ACID_METABOLISM	0.48529412	0.66637401	0.09196861	-0.3306536	-0.9943969
KEGG_LIMONENE_AND_PINENE_DEGRADATION	0.49059829	0.6686673	0.07200331	0.39894496	0.98769731
KEGG_INSULIN_SIGNALING_PATHWAY	0.49428571	0.6687395	0.0628004	0.21843452	0.9803606
KEGG_PRIMARY_IMMUNODEFICIENCY	0.50086059	0.67268867	0.0713053	0.28320402	0.98219669
KEGG_MTOR_SIGNALING_PATHWAY	0.5136	0.67987338	0.06643641	0.25597555	0.96623034
KEGG_PROXIMAL_TUBULE_BICARBONATE_RECLAMATION	0.51034483	0.67987338	0.07045009	0.31209381	0.95089069
KEGG_CYSTEINE_AND_METHIONINE_METABOLISM	0.52483444	0.68489033	0.06705126	0.28329304	0.95204038
KEGG_MATURITY_ONSET_DIABETES_OF_THE_YOUNG	0.5215311	0.68489033	0.08653997	-0.3516848	-0.9559355
KEGG_RIBOFLAVIN_METABOLISM	0.54081633	0.70077609	0.06689663	0.34484013	0.94322571
KEGG_VEGF_SIGNALING_PATHWAY	0.56097561	0.72181477	0.08943668	-0.2200064	-0.9454117
KEGG_ARRHYTHMOGENIC_RIGHT_VENTRICULAR_CARDIOMYOPA	0.56651017	0.72387411	0.0606404	0.23096992	0.93533025
KEGG_ALPHA_LINOLENIC_ACID_METABOLISM	0.57446809	0.72898019	0.08063885	-0.3094851	-0.893373
KEGG_PRIMARY_BILE_ACID_BIOSYNTHESIS	0.60245184	0.75925437	0.06307904	0.34457756	0.89747149
KEGG_AMYOTROPHIC_LATERAL_SCLEROSIS_ALS	0.60674157	0.75945884	0.05859376	0.24980304	0.92246367
KEGG_RNA_DEGRADATION	0.62303665	0.7745861	0.08175156	-0.2228187	-0.9177736
KEGG_AMINOACYL_TRNA_BIOSYNTHESIS	0.63426689	0.78325575	0.05773085	0.24861446	0.89234507
KEGG_OXIDATIVE_PHOSPHORYLATION	0.64801178	0.78963025	0.05153091	0.20738344	0.90709569
KEGG_RENIN_ANGIOTENSIN_SYSTEM	0.6440678	0.78963025	0.07588869	-0.2752184	-0.8716075
KEGG_NEUROTROPHIN_SIGNALING_PATHWAY	0.65359477	0.79119367	0.09139243	-0.1947495	-0.9338446
KEGG_MAPK_SIGNALING_PATHWAY	0.66015625	0.7939134	0.10135074	-0.1790124	-0.9462895
KEGG_BLADDER_CANCER	0.66582278	0.79552852	0.07647671	-0.2320964	-0.8954172
KEGG_ARACHIDONIC_ACID_METABOLISM	0.67839196	0.80531691	0.07511816	-0.2289734	-0.8885239
KEGG_GLYOXYLATE_AND_DICARBOXYLATE_METABOLISM	0.68518519	0.80816714	0.05513518	0.29789748	0.83109022
KEGG_PHENYLALANINE_METABOLISM	0.69117647	0.81004121	0.07289386	-0.2779091	-0.8357749
KEGG_GLYCEROPHOSPHOLIPID_METABOLISM	0.70109546	0.8164656	0.05070279	0.21670435	0.87756075
KEGG_GLYCOSPHINGOLIPID_BIOSYNTHESIS_LACTO_AND_NEOLA(	0.72173913	0.83522013	0.05423159	0.27266196	0.81953024
KEGG_ASCORBATE_AND_ALDARATE_METABOLISM	0.75831874	0.87206655	0.05226933	0.29618069	0.77141913
KEGG_GLYCINE_SERINE_AND_THREONINE_METABOLISM	0.76859504	0.87506173	0.04899541	0.24465748	0.79620738
KEGG_ASTHMA	0.77043478	0.87506173	0.05121843	0.25920581	0.77908558
KEGG_GLYCOSPHINGOLIPID_BIOSYNTHESIS_GLOBO_SERIES	0.80510441	0.90882952	0.06266182	-0.2538447	-0.7163988
KEGG_BASAL_TRANSCRIPTION_FACTORS	0.81280788	0.91193079	0.06508776	-0.2164482	-0.8061668
KEGG_CALCIUM_SIGNALING_PATHWAY	0.82295082	0.91771485	0.07892096	-0.1723413	-0.8693718
KEGG_STEROID_BIOSYNTHESIS	0.82857143	0.91841652	0.06252374	-0.2352174	-0.7288339

KEGG_THYROID_CANCER	0.87660668	0.96230682	0.0635008	-0.2051067	-0.728533
KEGG_NOTCH_SIGNALING_PATHWAY	0.87862797	0.96230682	0.06464846	-0.1955119	-0.7653367
KEGG_ALZHEIMERS_DISEASE	0.89146165	0.97058546	0.036073	0.1731302	0.79166198
KEGG_JAK_STAT_SIGNALING_PATHWAY	0.92732558	0.978097	0.03441748	0.17022447	0.75696774
KEGG_ACUTE_MYELOID_LEUKEMIA	0.90645161	0.978097	0.04032954	0.19319598	0.74465682
KEGG_TOLL_LIKE_RECEPTOR_SIGNALING_PATHWAY	0.94162826	0.978097	0.03630185	0.17515869	0.73049059
KEGG_APOPTOSIS	0.94620253	0.978097	0.03745842	0.17384997	0.7186753
KEGG_LONG_TERM_DEPRESSION	0.94051447	0.978097	0.03847869	0.18554059	0.71790191
KEGG_CYTOSOLIC_DNA_SENSING_PATHWAY	0.91708126	0.978097	0.04107133	0.19324871	0.69148287
KEGG_RNA_POLYMERASE	0.91390728	0.978097	0.0411544	0.20569539	0.69126413
KEGG_PORPHYRIN_AND_CHLOROPHYLL_METABOLISM	0.93637847	0.978097	0.0393562	0.19723755	0.65972578
KEGG_RIG_I_LIKE_RECEPTOR_SIGNALING_PATHWAY	0.921875	0.978097	0.06170541	-0.1859919	-0.754437
KEGG_SELENOAMINO_ACID_METABOLISM	0.95278246	0.97892368	0.04008456	0.19739622	0.62532341
KEGG_GLYCOSYLPHOSPHATIDYLINOSITOL_GPI_ANCHOR_BIOSYNT	0.96296296	0.97892368	0.05712585	-0.1821563	-0.6292631
KEGG_INOSITOL_PHOSPHATE_METABOLISM	0.95800525	0.97892368	0.06024841	-0.1735286	-0.708436
KEGG_PURINE_METABOLISM	0.97749196	0.98823363	0.06911985	-0.150372	-0.7530836
KEGG_HUNTINGTONS_DISEASE	1	1	0.0292811	0.13035983	0.60353616
KEGG_REGULATION_OF_AUTOPHAGY	0.9965338	1	0.03927586	0.13711847	0.41430307
