# Supplementary material for: Bone dysplasia in Hutchinson‐Gilford progeria syndrome is associated with dysregulated differentiation and function of bone cell populations
Source: Aging Cell. 2023 Jun 26;22(9):e13903. doi: 10.1111/acel.13903 (PMC10497813; doi:10.1111/acel.13903)
Supplement: Supplementary file 2 — Data File S2. [file ACEL-22-e13903-s003.pdf]

Supplemental Data File 2. RNA-seq quality metrics from QoRTs

All columns were generated by QoRTs. "TOTAL_READ_PAIRS" was generated by QoRTs and represents the number of reads in the BAM files provided to QoRTs after read alignment. Please refer to the QoRTs documentation for descriptions of other columns.

FIELD	READ_PAIR_OK	TOTAL_READ_PAIRS	DROPPED_IGNORED_CHROMOSOME	DROPPED_NOT_UNIQUE_ALIGNMENT	minObservedReadLength	maxObservedReadLength	ReadPairs_AmbigGene	ReadPairs_UniqueGene	ReadPairs_UniqueGene_CDS	ReadPairs_UniqueGene_UTR	ReadPairs_NoGene	ReadPairs_NoGene_Intron	ReadPairs_NoGene_OneKbFromGene	ReadPairs_NoGene_TenKbFromGene	ReadPairs_NoGene_MiddleOfNowhere
WC42_RG1	111,551,665	130,836,665	5,114,919	14,170,081	151	151	3,044,516	95,700,816	64,712,322	30,988,494	12,806,333	9,473,041	606,508	1,340,245	1,386,539
WC43_RG1	101,497,393	116,967,773	3,593,500	11,876,880	151	151	2,735,014	87,517,465	59,828,799	27,688,666	11,244,914	8,376,652	533,394	1,164,748	1,170,120
WC44_RG1	97,563,546	115,433,776	4,056,761	13,813,469	151	151	2,990,662	81,830,266	54,780,479	27,049,787	12,742,618	9,558,553	558,367	1,319,329	1,306,369
WC45_RG1	78,999,099	92,815,754	4,151,671	9,664,984	151	151	1,980,456	70,345,962	46,644,267	23,701,695	6,672,681	4,515,505	417,447	876,333	863,396
WC46_RG1	106,632,419	121,636,997	3,958,410	11,046,168	151	151	2,569,827	92,525,157	62,759,915	29,765,242	11,537,435	8,446,880	562,133	1,260,106	1,268,316
WC47_RG1	94,971,802	108,831,704	3,489,965	10,369,937	151	151	2,421,524	81,172,346	54,561,416	26,610,930	11,377,932	8,447,346	537,936	1,208,414	1,184,236
WC48_RG1	91,282,573	106,550,630	3,602,133	11,665,924	151	151	2,721,153	77,925,163	52,573,329	25,351,834	10,636,257	7,775,877	504,798	1,159,837	1,195,745
WC49_RG1	87,996,105	102,667,612	3,527,577	11,143,930	151	151	2,547,159	74,752,175	49,546,252	25,205,923	10,696,771	7,884,662	513,287	1,141,372	1,157,450
WC50_RG1	93,446,105	108,106,387	3,548,906	11,111,376	151	151	2,445,159	78,688,021	52,487,227	26,200,794	12,312,925	9,286,193	530,173	1,231,326	1,265,233
WC51_RG1	73,661,087	87,025,263	3,873,619	9,490,557	151	151	1,879,793	63,417,174	41,883,516	21,533,658	8,364,120	6,103,227	405,866	917,557	937,470
WC52_RG1	86,958,442	100,949,508	3,476,071	10,514,995	151	151	2,289,908	73,850,263	48,950,467	24,899,796	10,818,271	7,975,141	514,837	1,199,464	1,128,829
WC53_RG1	87,501,326	100,803,865	3,279,479	10,023,060	151	151	2,410,331	73,276,809	49,222,268	24,054,541	11,814,186	8,845,035	515,178	1,211,777	1,242,196
WC54_RG1	186,370,075	213,441,707	6,216,341	20,855,291	151	151	5,191,820	159,264,957	107,755,886	51,509,071	21,913,298	16,419,524	1,038,456	2,269,644	2,185,674
WC55_RG1	199,437,133	225,892,403	5,293,114	21,162,156	151	151	6,084,566	169,454,698	115,231,183	54,223,515	23,897,869	17,742,554	1,222,723	2,457,556	2,475,036
WC56_RG1	150,569,068	175,930,099	5,526,159	19,834,872	151	151	5,023,367	128,779,397	83,741,468	45,037,929	16,766,304	12,336,273	886,459	1,835,579	1,707,993
WC57_RG1	204,263,329	233,926,467	6,515,594	23,147,544	151	151	5,629,888	173,761,343	116,966,428	56,794,915	24,872,098	19,139,510	1,138,339	2,391,304	2,202,945
WC58_RG1	201,748,394	232,471,216	6,878,932	23,843,890	151	151	5,818,486	173,448,283	114,225,508	59,222,775	22,481,625	16,871,624	1,116,063	2,330,560	2,163,378
WC59_RG1	181,611,981	211,005,480	5,925,808	23,467,691	151	151	5,789,723	158,011,208	103,963,029	54,048,179	17,811,050	12,390,528	1,125,406	2,273,805	2,021,311
WC60_RG1	175,360,655	203,693,674	5,399,231	22,933,788	151	151	5,812,548	150,599,556	102,693,871	47,905,685	18,948,551	14,018,855	969,726	2,014,574	1,945,396
WC61_RG1	165,931,520	192,006,843	6,435,775	19,639,548	151	151	4,776,254	141,932,293	91,964,465	49,967,828	19,222,973	14,113,614	1,002,902	2,118,455	1,988,002
WC62_RG1	159,299,788	196,861,270	14,440,050	23,121,432	151	151	4,289,552	142,320,653	94,763,212	47,557,441	12,689,583	8,571,588	911,376	1,767,940	1,438,679
WC63_RG1	227,748,943	262,610,942	5,727,809	29,134,190	151	151	7,381,785	193,206,121	132,266,318	60,939,803	27,161,037	20,641,832	1,205,122	2,675,876	2,638,207
WC64_RG1	174,107,013	201,289,271	5,698,643	21,483,615	151	151	5,105,695	148,704,926	97,158,750	51,546,176	20,296,392	15,025,799	994,687	2,226,647	2,049,259
WC65_RG1	173,674,449	205,201,086	6,965,045	24,561,592	151	151	5,934,068	150,039,183	98,939,342	51,099,841	17,701,198	12,267,335	1,106,659	2,292,994	2,034,210
WC13_RG4	18,527,227	20,074,360	378,430	1,168,703	76	76	431,987	17,078,384	12,475,411	4,602,973	1,016,856	801,706	50,854	92,997	71,299
WC13_RG5	18,645,975	20,206,663	384,251	1,176,437	76	76	432,709	17,182,774	12,538,573	4,644,201	1,030,492	813,207	51,012	94,071	72,202
WC13_RG6	18,639,824	20,209,505	385,418	1,184,263	76	76	433,565	17,179,224	12,527,951	4,651,273	1,027,035	810,032	51,581	93,020	72,402
WC11_RG2	19,330,448	20,955,804	522,079	1,103,277	76	76	374,735	17,781,941	13,108,519	4,673,422	1,173,772	895,022	61,048	118,877	98,825
WC11_RG3	19,356,576	20,992,248	526,027	1,109,645	76	76	376,338	17,804,275	13,112,460	4,691,815	1,175,963	895,839	62,013	118,991	99,120
WC11_RG4	19,291,320	20,900,225	516,436	1,092,469	76	76	375,398	17,741,676	13,107,858	4,633,818	1,174,246	893,713	60,180	119,530	100,823
WC11_RG5	19,482,415	21,112,813	526,497	1,103,901	76	76	379,638	17,910,826	13,220,413	4,690,413	1,191,951	910,113	60,946	120,333	100,559
WC11_RG6	19,506,621	21,144,490	528,773	1,109,096	76	76	379,450	17,934,290	13,231,865	4,702,425	1,192,881	909,219	61,649	121,178	100,835
WC12_RG2	19,347,649	20,952,122	526,325	1,078,148	76	76	373,048	17,860,932	13,151,807	4,709,125	1,113,669	837,503	60,673	118,145	97,348
WC12_RG3	19,335,653	20,947,175	525,720	1,085,802	76	76	371,345	17,848,451	13,132,634	4,715,817	1,115,857	838,052	60,909	119,305	97,591
WC12_RG4	19,401,606	20,995,161	523,638	1,069,917	76	76	376,130	17,906,623	13,226,159	4,680,464	1,118,853	841,378	60,131	119,770	97,574
WC12_RG5	19,585,139	21,203,120	533,142	1,084,839	76	76	379,740	18,069,430	13,330,519	4,738,911	1,135,969	855,410	60,429	121,366	98,764
WC12_RG6	19,581,519	21,200,684	530,819	1,088,346	76	76	378,687	18,068,315	13,327,157	4,741,158	1,134,517	854,284	61,521	120,194	98,518
WC8_RG2	16,876,664	18,284,198	406,874	1,000,660	76	76	408,959	15,506,911	11,013,006	4,493,905	960,794	682,228	81,064	109,640	87,862
WC8_RG3	16,875,405	18,288,940	407,265	1,006,270	76	76	407,456	15,503,032	10,992,615	4,510,417	964,917	684,649	81,782	110,060	88,426
WC8_RG4	17,001,264	18,406,130	406,438	998,428	76	76	412,676	15,618,923	11,121,824	4,497,099	969,665	690,409	80,161	111,078	88,017
WC8_RG5	17,146,498	18,567,415	413,423	1,007,494	76	76	415,371	15,752,965	11,215,291	4,537,674	978,162	698,081	78,766	111,854	89,461
WC8_RG6	17,139,431	18,567,101	413,726	1,013,944	76	76	414,820	15,748,296	11,199,866	4,548,430	976,315	695,532	79,500	111,906	89,377
WC1_RG2	19,532,541	21,141,058	397,017	1,211,500	76	76	467,650	17,957,152	12,996,361	4,960,791	1,107,739	872,524	55,401	103,462	76,352
WC1_RG3	19,493,027	21,108,600	395,904	1,219,669	76	76	466,356	17,915,475	12,950,457	4,965,018	1,111,196	875,162	55,408	104,363	76,263
WC1_RG4	19,703,588	21,309,621	396,789	1,209,244	76	76	472,054	18,110,471	13,147,640	4,962,831	1,121,063	883,082	55,380	105,496	77,105
WC1_RG5	19,794,314	21,416,195	400,340	1,221,541	76	76	472,156	18,190,591	13,200,643	4,989,948	1,131,567	891,929	55,452	106,439	77,747
WC1_RG6	19,783,287	21,411,814	401,002	1,227,525	76	76	472,817	18,182,073	13,181,529	5,000,544	1,128,397	890,740	55,031	105,061	77,565
WC10_RG2	22,921,795	24,887,729	622,823	1,343,111	76	76	429,394	21,215,948	15,578,591	5,637,357	1,276,453	955,022	73,910	139,843	107,678
WC10_RG3	22,884,941	24,861,365	621,580	1,354,844	76	76	429,635	21,182,686	15,535,069	5,647,617	1,272,620	951,389	73,355	139,479	108,397
WC10_RG4	22,913,396	24,862,958	616,238	1,333,324	76	76	432,162	21,205,532	15,608,785	5,596,747	1,275,702	954,362	72,336	140,484	108,520
WC10_RG5	23,185,049	25,162,900	629,322	1,348,529	76	76	438,456	21,450,664	15,769,986	5,680,678	1,295,929	970,150	71,883	143,893	110,003
WC18_RG6	19,880,523	21,512,308	562,501	1,069,284	76	76	423,139	18,143,000	13,206,186	4,936,814	1,314,384	987,538	66,814	145,519	114,513
WC15_RG2	19,598,834	21,332,592	626,885	1,106,873	76	76	439,043	17,729,413	12,569,579	5,159,834	1,430,378	1,102,229	72,509	147,652	107,988
WC15_RG3	19,587,537	21,325,472	627,273	1,110,662	76	76	438,501	17,716,494	12,542,560	5,173,934	1,432,542	1,104,216	72,447	148,125	107,754
WC15_RG4	19,735,084	21,461,995	625,122	1,101,789	76	76	443,941	17,848,276	12,688,698	5,159,578	1,442,867	1,111,488	72,163	150,393	108,823
WC15_RG5	19,903,161	21,653,138	635,383	1,114,594	76	76	445,240	17,994,629	12,782,979	5,211,650	1,463,292	1,127,228	72,601	152,500	110,963
WC15_RG6	19,875,701	21,631,450	635,913	1,119,836	76	76	444,136	17,975,301	12,758,310	5,216,991	1,456,264	1,121,907	72,339	151,881	110,137
WC10_RG6	23,154,821	25,138,566	628,976	1,354,769	76	76	436,736	21,426,905	15,751,078	5,675,827	1,291,180	966,582	73,022	141,626	109,950
WC2_RG2	21,681,985	23,551,690	560,593	1,309,112	76	76	531,467	19,978,342	14,327,662	5,650,680	1,172,176	879,874	69,653	125,559	97,090
WC7_RG2	19,822,094	21,433,304	393,314	1,217,896	76	76	475,612	18,206,148	13,219,685	4,986,463	1,140,334	904,054	57,817	101,919	76,544
WC2_RG3	21,705,125	23,584,236	560,540	1,318,571	76	76	532,104	19,997,699	14,318,352	5,679,347	1,175,322	882,142	70,647	125,178	97,355
WC7_RG3	19,792,465	21,411,089	393,958	1,224,666	76	76	475,011	18,176,630	13,183,137	4,993,493	1,140,824	903,596	57,343	102,166	77,719
WC2_RG4	21,610,834	23,453,468	553,039	1,289,595	76	76	533,182	19,914,167	14,327,851	5,586,316	1,163,485	874,514	68,080	124,674	96,217
WC7_RG4	20,018,742	21,634,262	394,592	1,220,928	76	76	480,801	18,385,446	13,395,024	4,990,422	1,152,495	913,312	56,717	103,867	78,599
WC7_RG5	20,212,700	21,845,051	401,323	1,231,028	76	76	483,862	18,559,296	13,508,880	5,050,416	1,169,542	927,598	57,521	104,541	79,882
WC7_RG6	20,168,689	21,806,223	400,528	1,237,006	76	76	484,353	18,520,506	13,474,859	5,045,647	1,163,830	922,660	57,881	104,298	78,991
WC5_RG2	17,442,618	18,901,280	457,214	1,001,448	76	76	346,703	16,089,640	11,757,654	4,331,986	1,006,275	756,075	56,216	106,808	87,176
WC5_RG3	17,470,594	18,941,290	460,902	1,009,794	76	76	346,399	16,113,439	11,765,223	4,348,216	1,010,756	758,961	56,430	107,437	87,928
WC5_RG4	17,426,875	18,871,319	453,533	990,911	76	76	348,866	16,071,045	11,777,095	4,293,950	1,006,964	757,003	55,566	107,292	87,103
WC5_RG5	17,529,592	18,992,214	461,858	1,000,764	76	76	349,813	16,165,054	11,834,772	4,330,282	1,014,725	762,453	55,370	108,370	88,532
WC5_RG6	17,599,869	19,071,508	463,296	1,008,343	76	76	350,823	16,232,063	11,876,450	4,355,613	1,016,983	764,662	55,843	108,731	87,747
WC18_RG2	19,581,125	21,186,596	550,832	1,054,639	76	76	416,203	17,877,055	13,001,680	4,875,375	1,287,867	967,036	66,724	142,330	111,777
WC18_RG3	19,569,318	21,182,265	552,023	1,060,924	76	76	415,067	17,864,013	12,974,608	4,889,405	1,290,238	967,883	67,246	142,760	112,349
WC18_RG4	19,750,110	21,356,229	553,916	1,052,203	76	76	421,334	18,024,419	13,141,058	4,883,361	1,304,357	979,326	66,874	145,092	113,065
WC18_RG5	19,890,213	21,518,430	562,698	1,065,519	76	76	423,553	18,148,305	13,219,957	4,928,348	1,318,355	991,532	66,662	146,124	114,037
WC2_RG5	21,855,044	23,726,298	562,940	1,308,314	76	76	537,097	20,133,819	14,469,266	5,664,553	1,184,128	890,226	68,833	127,353	97,716
WC2_RG6	21,857,581	23,734,259	564,492	1,312,186	76	76	535,849	20,138,322	14,458,180	5,680,142	1,183,410	889,298	69,383	126,497	98,232
WC16_RG2	19,032,214	20,642,992	564,531	1,046,247	76	76	400,367	17,465,566	12,651,860	4,813,706	1,166,281	872,896	64,200	131,219	97,966
WC16_RG3	19,023,942	20,643,334	564,843	1,054,549	76	76	400,469	17,455,629	12,634,740	4,820,889	1,167,844	873,716	65,240	131,206	97,682
WC16_RG4	19,210,670	20,822,354	566,564	1,045,120	76	76	406,362	17,624,009	12,800,199	4,823,810	1,180,299	883,448	64,882	132,918	99,051
WC16_RG5	19,359,477	20,992,414	576,418	1,056,519	76	76	407,898	17,757,443	12,888,955	4,868,488	1,194,136	893,753	64,579	135,393	100,411
WC16_RG6	19,322,418	20,956,000	573,697	1,059,885	76	76	408,291	17,724,909	12,864,577	4,860,332	1,189,218	890,719	65,167	133,998	99,334
WC13_RG2	18,384,745	19,931,826	378,224	1,168,857	76	76	428,182	16,945,719	12,340,104	4,605,615	1,010,844	796,772	51,263	91,741	71,068
WC13_RG3	18,404,244	19,964,203	382,016	1,177,943	76	76	427,614	16,964,501	12,335,695	4,628,806	1,012,129	797,894	51,702	91,910	70,623
WC6_RG3	19,577,241	21,254,887	563,905	1,113,741	76	76	372,690	18,021,467	13,142,428	4,879,039	1,183,084	855,960	82,293	130,719	114,112
WC6_RG4	19,557,472	21,208,608	556,733	1,094,403	76	76	375,425	18,004,804	13,182,928	4,821,876	1,177,243	853,555	79,970	130,619	113,099
WC6_RG5	19,725,540	21,395,554	565,644	1,104,370	76	76	377,709	18,156,679	13,275,300	4,881,379	1,191,152	866,883	78,817	131,588	113,864
WC6_RG6	19,733,013	21,412,098	567,932	1,111,153	76	76	378,291	18,164,631	13,280,094	4,884,537	1,190,091	864,759	80,342	131,511	113,479
WC9_RG2	17,546,072	19,042,804	466,722	1,030,010	76	76	351,166	15,797,148	11,240,209	4,556,939	1,397,758	879,470	171,456	165,146	181,686
WC9_RG3	17,600,314	19,108,279	468,690	1,039,275	76	76	352,348	15,843,242	11,261,385	4,581,857	1,404,724	880,079	172,078	168,180	184,387
WC9_RG4	17,655,711	19,149,324	468,302	1,025,311	76	76	356,211	15,907,003	11,356,928	4,550,075	1,392,497	880,932	165,725	165,999	179,841
WC9_RG5	17,829,137	19,344,523	475,790	1,039,596	76	76	358,011	16,068,732	11,472,091	4,596,641	1,402,394	894,897	160,618	166,349	180,530
WC9_RG6	17,840,327	19,361,273	477,073	1,043,873	76	76	359,006	16,074,954	11,468,450	4,606,504	1,406,367	894,035	164,571	166,749	181,012
WC17_RG2	21,824,425	23,616,741	608,462	1,183,854	76	76	468,645	19,936,480	14,473,979	5,462,501	1,419,300	1,072,214	73,753	153,599	119,734
WC17_RG3	21,738,815	23,527,999	605,057	1,184,127	76	76	467,667	19,853,711	14,393,683	5,460,028	1,417,437	1,070,218	73,910	153,987	119,322
WC17_RG4	22,161,789	23,970,489	616,182	1,192,518	76	76	480,562	20,235,245	14,726,231	5,509,014	1,445,982	1,092,025	74,250	157,702	122,005
WC17_RG5	22,357,244	24,189,681	625,355	1,207,082	76	76	483,408	20,407,952	14,838,232	5,569,720	1,465,884	1,108,219	74,198	159,614	123,853
WC17_RG6	22,228,866	24,057,154	623,340	1,204,948	76	76	478,647	20,298,401	14,749,585	5,548,816	1,451,818	1,097,082	74,488	157,337	122,911
WC4_RG2	16,707,840	18,123,474	438,497	977,137	76	76	322,698	15,554,134	11,438,375	4,115,759	831,008	602,045	53,986	98,404	76,573
WC4_RG3	16,729,171	18,156,560	441,282	986,107	76	76	320,854	15,575,876	11,433,170	4,142,706	832,441	602,352	53,474	99,123	77,492
WC4_RG4	16,718,195	18,122,241	435,685	968,361	76	76	321,870	15,566,154	11,475,780	4,090,374	830,171	602,098	52,721	99,007	76,345
WC4_RG5	16,843,114	18,262,182	442,892	976,176	76	76	325,533	15,678,553	11,547,837	4,130,716	839,028	608,585	52,752	100,130	77,561
WC4_RG6	16,878,768	18,303,158	442,527	981,863	76	76	324,435	15,713,359	11,566,263	4,147,096	840,974	610,329	53,059	100,087	77,499
WC14_RG2	21,688,621	23,501,791	566,174	1,246,996	76	76	549,774	19,867,120	14,099,212	5,767,908	1,271,727	954,949	74,704	133,738	108,336
WC14_RG3	21,668,098	23,490,101	567,678	1,254,325	76	76	549,195	19,845,568	14,063,719	5,781,849	1,273,335	956,575	74,842	133,817	108,101
WC14_RG4	21,682,835	23,479,439	560,987	1,235,617	76	76	552,176	19,859,263	14,141,255	5,718,008	1,271,396	956,414	73,892	133,359	107,731
WC14_RG5	21,915,887	23,742,813	572,509	1,254,417	76	76	556,025	20,065,280	14,274,748	5,790,532	1,294,582	974,786	73,588	136,447	109,761
WC14_RG6	21,878,488	23,710,653	572,728	1,259,437	76	76	555,311	20,036,093	14,239,914	5,796,179	1,287,084	968,004	74,015	136,037	109,028
WC3_RG1	15,472,274	16,759,264	410,639	876,351	76	76	324,661	14,241,021	10,391,555	3,849,466	906,592	691,896	49,516	93,172	72,008
WC6_RG1	18,952,909	20,575,568	544,243	1,078,416	76	76	358,580	17,443,741	12,701,901	4,741,840	1,150,588	829,500	81,524	126,838	112,726
WC9_RG1	16,714,980	18,150,049	442,706	992,363	76	76	332,160	15,026,966	10,654,099	4,372,867	1,355,854	835,954	172,697	163,912	183,291
WC17_RG1	21,025,753	22,755,357	582,631	1,146,973	76	76	452,295	19,206,402	13,906,086	5,300,316	1,367,056	1,031,504	72,023	147,730	115,799
WC4_RG1	16,145,126	17,520,892	424,004	951,762	76	76	309,835	15,031,465	11,022,623	4,008,842	803,826	581,104	52,157	94,868	75,697
WC14_RG1	20,946,966	22,708,584	545,349	1,216,269	76	76	529,163	19,182,611	13,570,471	5,612,140	1,235,192	925,287	74,407	129,822	105,676
WC1_RG1	18,848,087	20,412,332	382,123	1,182,122	76	76	450,038	17,322,272	12,497,087	4,825,185	1,075,777	845,512	55,063	100,120	75,082
WC10_RG1	22,190,617	24,100,087	598,739	1,310,731	76	76	414,417	20,542,528	15,052,317	5,490,211	1,233,672	921,367	72,051	134,330	105,924
WC7_RG1	18,982,562	20,539,618	377,830	1,179,226	76	76	453,232	17,434,007	12,622,594	4,811,413	1,095,323	864,447	56,262	98,470	76,144
WC5_RG1	16,841,301	18,256,974	441,062	974,611	76	76	333,617	15,533,630	11,327,001	4,206,629	974,054	729,243	55,843	103,134	85,834
WC18_RG1	18,788,904	20,337,582	530,173	1,018,505	76	76	396,281	17,154,768	12,442,111	4,712,657	1,237,855	926,953	65,580	136,820	108,502
WC15_RG1	18,881,955	20,555,751	600,868	1,072,928	76	76	419,713	17,082,017	12,075,703	5,006,314	1,380,225	1,061,648	71,002	142,986	104,589
WC2_RG1	20,914,331	22,726,082	539,547	1,272,204	76	76	509,560	19,272,175	13,775,767	5,496,408	1,132,596	847,853	69,084	120,970	94,689
WC16_RG1	18,304,885	19,863,180	544,036	1,014,259	76	76	383,300	16,796,802	12,137,432	4,659,370	1,124,783	839,385	63,254	127,160	94,984
WC13_RG1	17,735,697	19,241,027	365,958	1,139,372	76	76	411,958	16,346,811	11,863,402	4,483,409	976,928	767,856	50,773	88,904	69,395
WC11_RG1	18,635,331	20,209,744	502,671	1,071,742	76	76	358,089	17,144,110	12,608,281	4,535,829	1,133,132	859,866	60,921	115,122	97,223
WC12_RG1	18,668,605	20,223,116	507,235	1,047,276	76	76	357,482	17,234,094	12,667,853	4,566,241	1,077,029	807,726	60,104	113,990	95,209
WC8_RG1	16,174,245	17,529,989	390,276	965,468	76	76	390,413	14,855,171	10,514,267	4,340,904	928,661	654,614	81,222	105,901	86,924
WC3_RG2	16,158,799	17,497,994	428,456	910,739	76	76	341,425	14,867,568	10,872,400	3,995,168	949,806	726,011	50,983	97,216	75,596
WC3_RG3	16,210,389	17,559,473	429,366	919,718	76	76	341,807	14,916,784	10,899,238	4,017,546	951,798	727,831	51,132	97,334	75,501
WC3_RG4	16,229,189	17,561,737	426,565	905,983	76	76	344,741	14,930,437	10,947,431	3,983,006	954,011	729,406	50,305	99,068	75,232
WC3_RG5	16,357,447	17,707,698	433,977	916,274	76	76	346,084	15,044,950	11,019,673	4,025,277	966,413	740,114	50,488	99,751	76,060
WC3_RG6	16,401,490	17,758,093	435,871	920,732	76	76	346,726	15,088,582	11,045,165	4,043,417	966,182	740,115	50,856	99,058	76,153
WC6_RG2	19,585,533	21,253,451	561,287	1,106,631	76	76	374,501	18,028,907	13,163,627	4,865,280	1,182,125	856,070	81,828	131,324	112,903

FIELD	Genes_Total	Genes_WithZeroCounts	Genes_WithNonzeroCounts	AVG_GC	AggregateGenes_WithReads	SpliceLoci	SpliceLoci_Known	SpliceLoci_Novel	InsertSize_Mean	InsertSize_Median	GC_byPair_Mean	GC_byPair_Median	GC_byRead_Mean	GC_byRead_Median	GeneBodyCoverage_Overall_Mean	GeneBodyCoverage_Overall_Median	GeneBodyCoverage_LowExpress_Mean	GeneBodyCoverage_LowExpress_Median
WC42_RG1	56,246	26,423	29,823	0.49	29,133	627,657	288,578	339,079	660.94	269	147.02	149	73.51	75	0.49	0.49	0.49	0.49
WC43_RG1	56,246	27,479	28,767	0.49	28,103	602,087	288,578	313,509	652.85	282	147.13	149	73.56	75	0.48	0.49	0.49	0.49
WC44_RG1	56,246	26,622	29,624	0.48	28,953	607,918	288,578	319,340	713.75	275	146.42	149	73.21	75	0.49	0.49	0.49	0.49
WC45_RG1	56,246	27,656	28,590	0.49	27,956	549,178	288,578	260,600	609.5	261	147.84	150	73.92	75	0.5	0.51	0.49	0.49
WC46_RG1	56,246	26,812	29,434	0.49	28,774	609,846	288,578	321,268	622.3	277	147.84	150	73.92	75	0.49	0.49	0.49	0.49
WC47_RG1	56,246	27,072	29,174	0.49	28,511	588,123	288,578	299,545	656.57	280	146.87	149	73.44	75	0.49	0.49	0.49	0.49
WC48_RG1	56,246	27,033	29,213	0.49	28,544	594,528	288,578	305,950	683.54	273	147.93	150	73.97	75	0.49	0.49	0.49	0.49
WC49_RG1	56,246	27,095	29,151	0.49	28,487	577,099	288,578	288,521	684.15	269	146.84	149	73.42	75	0.5	0.51	0.49	0.49
WC50_RG1	56,246	27,159	29,087	0.48	28,426	574,057	288,578	285,479	650.29	277	145.74	148	72.87	74	0.49	0.49	0.49	0.49
WC51_RG1	56,246	27,130	29,116	0.49	28,458	547,581	288,578	259,003	627.78	272	147.04	149	73.52	75	0.5	0.51	0.49	0.49
WC52_RG1	56,246	27,068	29,178	0.48	28,500	591,505	288,578	302,927	636.82	273	145.92	148	72.96	74	0.49	0.49	0.49	0.49
WC53_RG1	56,246	26,945	29,301	0.49	28,617	586,870	288,578	298,292	662.9	272	146.81	149	73.41	75	0.49	0.49	0.49	0.49
WC54_RG1	56,246	25,430	30,816	0.49	30,114	717,884	288,578	429,306	690.37	269	147.13	150	73.57	75	0.48	0.49	0.49	0.49
WC55_RG1	56,246	25,122	31,124	0.49	30,404	772,601	288,578	484,023	731.16	270	148.48	151	74.24	75	0.48	0.49	0.49	0.49
WC56_RG1	56,246	26,229	30,017	0.49	29,328	643,047	288,578	354,469	760.12	261	147.17	150	73.58	75	0.51	0.51	0.5	0.51
WC57_RG1	56,246	25,486	30,760	0.48	30,054	725,297	288,578	436,719	676.1	262	145.46	148	72.73	74	0.48	0.49	0.5	0.51
WC58_RG1	56,246	25,361	30,885	0.48	30,173	717,429	288,578	428,851	702.15	259	146.43	149	73.21	75	0.5	0.51	0.5	0.49
WC59_RG1	56,246	25,869	30,377	0.49	29,676	686,194	288,578	397,616	729.94	262	146.72	149	73.36	75	0.49	0.49	0.5	0.51
WC60_RG1	56,246	25,850	30,396	0.49	29,700	686,509	288,578	397,931	767.19	256	148.12	151	74.06	75	0.48	0.49	0.49	0.49
WC61_RG1	56,246	25,679	30,567	0.49	29,873	658,215	288,578	369,637	681.98	251	147.66	150	73.83	75	0.51	0.51	0.5	0.51
WC62_RG1	56,246	26,159	30,087	0.49	29,398	648,044	288,578	359,466	629.25	262	147.66	151	73.83	75	0.51	0.54	0.51	0.51
WC63_RG1	56,246	24,948	31,298	0.49	30,574	812,068	288,578	523,490	775.36	281	147.54	150	73.77	75	0.48	0.49	0.49	0.49
WC64_RG1	56,246	27,230	29,016	0.48	28,351	565,316	288,578	276,738	704.16	273	145.42	148	72.71	74	0.5	0.51	0.5	0.51
WC65_RG1	56,246	26,369	29,877	0.48	29,189	650,237	288,578	361,659	789.07	266	144.86	147	72.43	74	0.49	0.49	0.5	0.51
WC13_RG4	56,246	35,604	20,642	0.51	20,182	330,760	288,578	42,182	444.06	267	77.72	79	38.86	40	0.46	0.46	0.46	0.46
WC13_RG5	56,246	35,596	20,650	0.51	20,197	329,644	288,578	41,066	447.84	269	77.61	79	38.81	39	0.47	0.46	0.47	0.46
WC13_RG6	56,246	35,537	20,709	0.51	20,233	328,951	288,578	40,373	443.25	266	77.67	79	38.83	40	0.46	0.46	0.46	0.46
WC11_RG2	56,246	35,290	20,956	0.52	20,496	328,018	288,578	39,440	431.36	257	78.44	80	39.22	40	0.47	0.46	0.46	0.46
WC11_RG3	56,246	35,340	20,906	0.52	20,452	327,218	288,578	38,640	431.56	254	78.42	80	39.21	40	0.47	0.46	0.46	0.46
WC11_RG4	56,246	35,224	21,022	0.52	20,545	329,356	288,578	40,778	441.49	264	78.42	80	39.21	40	0.47	0.46	0.46	0.46
WC11_RG5	56,246	35,253	20,993	0.52	20,538	328,547	288,578	39,969	446.63	267	78.32	80	39.16	40	0.47	0.46	0.46	0.46
WC11_RG6	56,246	35,331	20,915	0.52	20,456	328,068	288,578	39,490	442.89	264	78.36	80	39.18	40	0.47	0.46	0.46	0.46
WC12_RG2	56,246	35,320	20,926	0.52	20,463	329,004	288,578	40,426	437.53	261	78.47	80	39.24	40	0.48	0.46	0.46	0.46
WC12_RG3	56,246	35,356	20,890	0.52	20,424	328,472	288,578	39,894	435.38	258	78.44	80	39.22	40	0.48	0.46	0.47	0.46
WC12_RG4	56,246	35,287	20,959	0.52	20,493	331,029	288,578	42,451	448.15	269	78.45	80	39.23	40	0.48	0.46	0.46	0.46
WC12_RG5	56,246	35,246	21,000	0.52	20,532	329,798	288,578	41,220	451.51	272	78.34	80	39.17	40	0.48	0.49	0.47	0.46
WC12_RG6	56,246	35,294	20,952	0.52	20,487	329,270	288,578	40,692	447.32	268	78.39	80	39.2	40	0.48	0.49	0.47	0.46
WC8_RG2	56,246	35,524	20,722	0.51	20,277	325,419	288,578	36,841	443.42	264	77.75	79	38.87	40	0.47	0.46	0.47	0.46
WC8_RG3	56,246	35,452	20,794	0.51	20,343	324,644	288,578	36,066	444.43	261	77.72	79	38.86	40	0.47	0.46	0.46	0.46
WC8_RG4	56,246	35,434	20,812	0.51	20,374	326,484	288,578	37,906	455.84	272	77.72	79	38.86	40	0.47	0.46	0.47	0.46
WC8_RG5	56,246	35,450	20,796	0.51	20,357	325,384	288,578	36,806	460.85	274	77.63	79	38.82	39	0.47	0.46	0.47	0.46
WC8_RG6	56,246	35,400	20,846	0.51	20,400	325,707	288,578	37,129	456.86	271	77.68	79	38.84	40	0.47	0.46	0.47	0.46
WC1_RG2	56,246	35,563	20,683	0.51	20,234	329,411	288,578	40,833	432.44	261	77.42	79	38.71	39	0.47	0.46	0.47	0.46
WC1_RG3	56,246	35,490	20,756	0.51	20,319	328,703	288,578	40,125	432.73	259	77.4	79	38.7	39	0.47	0.46	0.47	0.46
WC1_RG4	56,246	35,310	20,936	0.51	20,462	331,018	288,578	42,440	444.64	269	77.41	79	38.71	39	0.47	0.46	0.47	0.46
WC1_RG5	56,246	35,418	20,828	0.51	20,360	329,436	288,578	40,858	448.73	271	77.3	79	38.65	39	0.47	0.46	0.47	0.46
WC1_RG6	56,246	35,434	20,812	0.51	20,367	329,336	288,578	40,758	445.41	268	77.36	79	38.68	39	0.47	0.46	0.47	0.46
WC10_RG2	56,246	35,156	21,090	0.51	20,627	334,080	288,578	45,502	434.99	261	78.26	79	39.13	40	0.47	0.46	0.47	0.46
WC10_RG3	56,246	35,118	21,128	0.51	20,655	333,004	288,578	44,426	435.94	258	78.23	79	39.12	40	0.47	0.46	0.47	0.46
WC10_RG4	56,246	34,926	21,320	0.51	20,834	335,450	288,578	46,872	447.22	270	78.24	79	39.12	40	0.47	0.46	0.47	0.46
WC10_RG5	56,246	35,023	21,223	0.51	20,752	334,245	288,578	45,667	453.24	272	78.14	79	39.07	40	0.48	0.46	0.47	0.46
WC18_RG6	56,246	34,340	21,906	0.51	21,408	335,973	288,578	47,395	461.29	270	77.55	79	38.78	39	0.47	0.46	0.47	0.46
WC15_RG2	56,246	34,511	21,735	0.51	21,255	332,204	288,578	43,626	445.04	259	77.05	78	38.53	39	0.47	0.46	0.47	0.46
WC15_RG3	56,246	34,527	21,719	0.51	21,232	331,321	288,578	42,743	445.89	257	77.02	78	38.51	39	0.47	0.46	0.47	0.46
WC15_RG4	56,246	34,445	21,801	0.51	21,309	333,843	288,578	45,265	458.99	267	77.01	78	38.51	39	0.48	0.46	0.47	0.46
WC15_RG5	56,246	34,433	21,813	0.51	21,308	332,777	288,578	44,199	462.64	270	76.93	78	38.47	39	0.48	0.49	0.47	0.46
WC15_RG6	56,246	34,477	21,769	0.51	21,279	332,305	288,578	43,727	457.94	266	76.98	78	38.49	39	0.48	0.46	0.47	0.46
WC10_RG6	56,246	34,995	21,251	0.51	20,774	333,913	288,578	45,335	448.06	268	78.19	79	39.09	40	0.48	0.46	0.47	0.46
WC2_RG2	56,246	35,074	21,172	0.51	20,704	331,760	288,578	43,182	438.48	257	77.93	79	38.97	40	0.47	0.46	0.46	0.46
WC7_RG2	56,246	35,544	20,702	0.51	20,243	330,050	288,578	41,472	444.05	268	77.58	79	38.79	39	0.46	0.46	0.46	0.46
WC2_RG3	56,246	34,983	21,263	0.51	20,811	330,559	288,578	41,981	438.83	255	77.9	79	38.95	40	0.47	0.46	0.47	0.46
WC7_RG3	56,246	35,523	20,723	0.51	20,261	328,905	288,578	40,327	443.68	265	77.55	79	38.77	39	0.46	0.46	0.46	0.46
WC2_RG4	56,246	34,935	21,311	0.51	20,839	332,981	288,578	44,403	450.85	266	77.92	79	38.96	40	0.47	0.46	0.47	0.46
WC7_RG4	56,246	35,295	20,951	0.51	20,479	331,852	288,578	43,274	455.83	277	77.55	79	38.77	39	0.47	0.46	0.46	0.46
WC7_RG5	56,246	35,393	20,853	0.51	20,383	330,611	288,578	42,033	459.69	279	77.46	79	38.73	39	0.47	0.46	0.47	0.46
WC7_RG6	56,246	35,364	20,882	0.51	20,401	330,176	288,578	41,598	455.93	275	77.51	79	38.76	39	0.47	0.46	0.47	0.46
WC5_RG2	56,246	35,620	20,626	0.52	20,176	324,459	288,578	35,881	426.84	255	78.4	80	39.2	40	0.48	0.49	0.47	0.46
WC5_RG3	56,246	35,656	20,590	0.52	20,128	324,046	288,578	35,468	427.14	253	78.38	80	39.19	40	0.48	0.49	0.47	0.46
WC5_RG4	56,246	35,518	20,728	0.52	20,278	325,707	288,578	37,129	437.38	262	78.4	80	39.2	40	0.48	0.49	0.47	0.46
WC5_RG5	56,246	35,557	20,689	0.52	20,239	325,002	288,578	36,424	441.7	265	78.29	80	39.14	40	0.48	0.49	0.47	0.46
WC5_RG6	56,246	35,556	20,690	0.52	20,245	324,184	288,578	35,606	439.07	262	78.33	80	39.16	40	0.48	0.49	0.47	0.46
WC18_RG2	56,246	34,468	21,778	0.51	21,292	335,489	288,578	46,911	447.93	263	77.63	79	38.82	40	0.47	0.46	0.46	0.46
WC18_RG3	56,246	34,361	21,885	0.51	21,399	334,868	288,578	46,290	447.56	260	77.61	79	38.81	39	0.47	0.46	0.47	0.46
WC18_RG4	56,246	34,218	22,028	0.51	21,540	337,079	288,578	48,501	461.04	272	77.59	79	38.79	39	0.47	0.46	0.47	0.46
WC18_RG5	56,246	34,270	21,976	0.51	21,478	336,185	288,578	47,607	465.62	274	77.49	79	38.75	39	0.48	0.46	0.47	0.46
WC2_RG5	56,246	34,886	21,360	0.51	20,887	331,704	288,578	43,126	455.53	269	77.81	79	38.91	40	0.47	0.46	0.47	0.46
WC2_RG6	56,246	34,866	21,380	0.51	20,926	331,759	288,578	43,181	453	265	77.86	79	38.93	40	0.47	0.46	0.47	0.46
WC16_RG2	56,246	34,791	21,455	0.51	20,980	331,300	288,578	42,722	441.41	259	77.68	79	38.84	40	0.48	0.46	0.47	0.46
WC16_RG3	56,246	34,841	21,405	0.51	20,932	330,365	288,578	41,787	441.96	256	77.67	79	38.83	40	0.48	0.46	0.47	0.46
WC16_RG4	56,246	34,664	21,582	0.51	21,104	332,963	288,578	44,385	454.87	267	77.65	79	38.83	40	0.48	0.46	0.47	0.46
WC16_RG5	56,246	34,692	21,554	0.51	21,070	331,551	288,578	42,973	459.08	269	77.56	79	38.78	40	0.48	0.49	0.47	0.46
WC16_RG6	56,246	34,743	21,503	0.51	21,027	331,647	288,578	43,069	454.13	266	77.62	79	38.81	40	0.48	0.46	0.47	0.46
WC13_RG2	56,246	35,763	20,483	0.51	20,030	329,211	288,578	40,633	432.53	259	77.74	79	38.87	40	0.46	0.46	0.46	0.46
WC13_RG3	56,246	35,801	20,445	0.51	19,992	328,863	288,578	40,285	432.22	256	77.72	79	38.86	40	0.46	0.46	0.46	0.46
WC6_RG3	56,246	35,340	20,906	0.52	20,443	326,330	288,578	37,752	429.26	254	78.43	80	39.22	40	0.48	0.49	0.47	0.46
WC6_RG4	56,246	35,181	21,065	0.52	20,605	328,351	288,578	39,773	440.94	265	78.44	80	39.22	40	0.48	0.49	0.47	0.46
WC6_RG5	56,246	35,269	20,977	0.52	20,510	327,217	288,578	38,639	444.76	267	78.33	80	39.16	40	0.48	0.49	0.47	0.46
WC6_RG6	56,246	35,324	20,922	0.52	20,446	326,649	288,578	38,071	441.67	264	78.38	80	39.19	40	0.48	0.49	0.47	0.46
WC9_RG2	56,246	35,476	20,770	0.52	20,308	325,313	288,578	36,735	434.8	264	78.38	80	39.19	40	0.47	0.46	0.47	0.46
WC9_RG3	56,246	35,456	20,790	0.52	20,335	324,223	288,578	35,645	435.1	262	78.35	80	39.17	40	0.47	0.46	0.47	0.46
WC9_RG4	56,246	35,414	20,832	0.52	20,380	326,534	288,578	37,956	445.42	272	78.33	80	39.17	40	0.47	0.46	0.47	0.46
WC9_RG5	56,246	35,396	20,850	0.51	20,403	325,326	288,578	36,748	451.1	275	78.23	80	39.12	40	0.47	0.46	0.47	0.46
WC9_RG6	56,246	35,413	20,833	0.52	20,387	325,143	288,578	36,565	446.75	271	78.28	80	39.14	40	0.47	0.46	0.47	0.46
WC17_RG2	56,246	34,358	21,888	0.51	21,397	337,449	288,578	48,871	449.88	264	77.93	79	38.96	40	0.47	0.46	0.47	0.46
WC17_RG3	56,246	34,258	21,988	0.51	21,485	336,540	288,578	47,962	448.38	262	77.92	79	38.96	40	0.47	0.46	0.46	0.46
WC17_RG4	56,246	34,166	22,080	0.51	21,575	340,137	288,578	51,559	462.2	274	77.85	79	38.92	40	0.47	0.46	0.47	0.46
WC17_RG5	56,246	34,275	21,971	0.51	21,469	338,192	288,578	49,614	467.81	276	77.78	79	38.89	40	0.48	0.46	0.47	0.46
WC17_RG6	56,246	34,213	22,033	0.51	21,531	337,666	288,578	49,088	463.34	272	77.83	79	38.92	40	0.47	0.46	0.46	0.46
WC4_RG2	56,246	35,924	20,322	0.52	19,890	323,870	288,578	35,292	430.25	257	78.77	80	39.39	40	0.48	0.46	0.47	0.46
WC4_RG3	56,246	35,913	20,333	0.52	19,891	322,977	288,578	34,399	429.58	254	78.74	80	39.37	40	0.48	0.46	0.47	0.46
WC4_RG4	56,246	35,753	20,493	0.52	20,051	324,700	288,578	36,122	440.32	264	78.77	80	39.39	40	0.48	0.46	0.47	0.46
WC4_RG5	56,246	35,950	20,296	0.52	19,853	323,411	288,578	34,833	444.15	267	78.65	80	39.33	40	0.48	0.49	0.47	0.46
WC4_RG6	56,246	35,912	20,334	0.52	19,910	323,422	288,578	34,844	440.68	263	78.7	80	39.35	40	0.48	0.46	0.47	0.46
WC14_RG2	56,246	34,784	21,462	0.51	20,986	333,060	288,578	44,482	440.07	256	77.44	79	38.72	39	0.47	0.46	0.47	0.46
WC14_RG3	56,246	34,710	21,536	0.51	21,063	332,666	288,578	44,088	440.77	254	77.41	79	38.71	39	0.47	0.46	0.47	0.46
WC14_RG4	56,246	34,527	21,719	0.51	21,230	334,937	288,578	46,359	451.73	265	77.42	79	38.71	39	0.47	0.46	0.47	0.46
WC14_RG5	56,246	34,658	21,588	0.51	21,118	333,578	288,578	45,000	455.79	267	77.32	79	38.66	39	0.47	0.46	0.47	0.46
WC14_RG6	56,246	34,611	21,635	0.51	21,144	333,363	288,578	44,785	453.28	263	77.37	79	38.68	39	0.47	0.46	0.47	0.46
WC3_RG1	56,246	35,678	20,568	0.52	20,126	323,234	288,578	34,656	418.81	246	78.43	80	39.22	40	0.48	0.46	0.47	0.46
WC6_RG1	56,246	35,248	20,998	0.52	20,542	328,361	288,578	39,783	419.89	245	78.51	80	39.25	40	0.48	0.49	0.46	0.46
WC9_RG1	56,246	35,498	20,748	0.52	20,292	325,486	288,578	36,908	424.31	252	78.46	80	39.23	40	0.47	0.46	0.47	0.46
WC17_RG1	56,246	34,292	21,954	0.51	21,461	338,722	288,578	50,144	438.97	253	78	79	39	40	0.47	0.46	0.46	0.46
WC4_RG1	56,246	35,937	20,309	0.52	19,851	324,522	288,578	35,944	419.05	245	78.83	80	39.42	40	0.47	0.46	0.47	0.46
WC14_RG1	56,246	34,631	21,615	0.51	21,128	334,969	288,578	46,391	430.26	245	77.49	79	38.74	39	0.47	0.46	0.47	0.46
WC1_RG1	56,246	35,409	20,837	0.51	20,357	330,102	288,578	41,524	422.91	249	77.48	79	38.74	39	0.47	0.46	0.47	0.46
WC10_RG1	56,246	35,110	21,136	0.52	20,657	335,053	288,578	46,475	425.47	249	78.34	80	39.17	40	0.47	0.46	0.47	0.46
WC7_RG1	56,246	35,414	20,832	0.51	20,361	330,541	288,578	41,963	433.39	257	77.62	79	38.81	39	0.46	0.46	0.47	0.46
WC5_RG1	56,246	35,556	20,690	0.52	20,245	325,524	288,578	36,946	417.13	243	78.48	80	39.24	40	0.48	0.49	0.47	0.46
WC18_RG1	56,246	34,268	21,978	0.51	21,479	336,265	288,578	47,687	438.57	251	77.69	79	38.84	40	0.47	0.46	0.46	0.46
WC15_RG1	56,246	34,476	21,770	0.51	21,264	333,110	288,578	44,532	436.34	248	77.1	78	38.55	39	0.47	0.46	0.47	0.46
WC2_RG1	56,246	34,925	21,321	0.51	20,845	333,321	288,578	44,743	427.8	245	77.99	79	39	40	0.47	0.46	0.46	0.46
WC16_RG1	56,246	34,653	21,593	0.51	21,103	331,972	288,578	43,394	431.83	247	77.73	79	38.87	40	0.47	0.46	0.47	0.46
WC13_RG1	56,246	35,555	20,691	0.51	20,216	330,116	288,578	41,538	421.21	247	77.79	79	38.9	40	0.46	0.46	0.46	0.46
WC11_RG1	56,246	35,253	20,993	0.52	20,526	328,978	288,578	40,400	419.3	245	78.5	80	39.25	40	0.47	0.46	0.46	0.46
WC12_RG1	56,246	35,285	20,961	0.52	20,495	330,324	288,578	41,746	427.18	249	78.54	80	39.27	40	0.48	0.46	0.47	0.46
WC8_RG1	56,246	35,434	20,812	0.51	20,351	325,946	288,578	37,368	435.57	252	77.81	79	38.9	40	0.47	0.46	0.47	0.46
WC3_RG2	56,246	35,625	20,621	0.52	20,157	322,473	288,578	33,895	428.66	258	78.37	80	39.19	40	0.48	0.49	0.47	0.46
WC3_RG3	56,246	35,694	20,552	0.52	20,116	322,101	288,578	33,523	429.73	256	78.35	80	39.17	40	0.48	0.49	0.47	0.46
WC3_RG4	56,246	35,458	20,788	0.52	20,327	323,722	288,578	35,144	438.27	265	78.36	80	39.18	40	0.48	0.49	0.47	0.46
WC3_RG5	56,246	35,671	20,575	0.51	20,116	322,539	288,578	33,961	442.65	268	78.25	79	39.13	40	0.48	0.49	0.47	0.46
WC3_RG6	56,246	35,515	20,731	0.52	20,284	322,610	288,578	34,032	440.31	265	78.3	80	39.15	40	0.48	0.49	0.47	0.46
WC6_RG2	56,246	35,341	20,905	0.52	20,430	327,313	288,578	38,735	430.33	257	78.45	80	39.23	40	0.48	0.49	0.46	0.46

FIELD	GeneBodyCoverage_UMQuartile_Mean	GeneBodyCoverage_UMQuartile_Median	geneDiversityProfile_top1count	geneDiversityProfile_top10count	geneDiversityProfile_top100count	geneDiversityProfile_top1000count	geneDiversityProfile_top1pct	geneDiversityProfile_top10pct	geneDiversityProfile_top100pct	geneDiversityProfile_top1000pct	input.read.pair.count	total.aligned.rate	aligned.and.pf.rate	multi.mapped.read.pair.count	multi.mapped.rate
WC42_RG1	0.49	0.49	2,786,083	11,333,494	21,253,161	46,427,631	0.03	0.12	0.22	0.49	147,816,624	0.89	0.75	14,170,081	0.1
WC43_RG1	0.48	0.49	1,574,149	9,200,175	18,359,367	42,118,033	0.02	0.11	0.21	0.48	130,967,938	0.89	0.77	11,876,880	0.09
WC44_RG1	0.49	0.49	1,981,091	8,501,433	16,806,016	38,952,488	0.02	0.1	0.21	0.48	130,147,577	0.89	0.75	13,813,469	0.11
WC45_RG1	0.49	0.49	2,603,106	8,711,930	16,228,323	34,178,502	0.04	0.12	0.23	0.49	106,445,893	0.87	0.74	9,664,984	0.09
WC46_RG1	0.48	0.49	3,451,571	10,993,181	20,743,098	44,613,662	0.04	0.12	0.22	0.48	136,451,909	0.89	0.78	11,046,168	0.08
WC47_RG1	0.49	0.49	2,571,340	9,159,607	17,700,307	39,092,757	0.03	0.11	0.22	0.48	123,544,847	0.88	0.77	10,369,937	0.08
WC48_RG1	0.49	0.49	1,632,396	8,409,622	16,587,567	37,476,365	0.02	0.11	0.21	0.48	122,030,682	0.87	0.75	11,665,924	0.1
WC49_RG1	0.49	0.49	1,399,015	7,025,047	14,991,120	35,392,940	0.02	0.09	0.2	0.47	117,182,714	0.88	0.75	11,143,930	0.1
WC50_RG1	0.49	0.49	1,435,952	7,579,088	15,787,363	37,245,575	0.02	0.1	0.2	0.47	123,983,058	0.87	0.75	11,111,376	0.09
WC51_RG1	0.5	0.51	2,524,058	8,530,844	15,405,837	31,358,268	0.04	0.13	0.24	0.49	99,157,852	0.88	0.74	9,490,557	0.1
WC52_RG1	0.49	0.49	1,133,366	6,113,576	13,908,092	34,339,034	0.02	0.08	0.19	0.46	113,007,210	0.89	0.77	10,514,995	0.09
WC53_RG1	0.48	0.49	1,209,285	6,187,802	13,826,216	33,729,542	0.02	0.08	0.19	0.46	115,016,879	0.88	0.76	10,023,060	0.09
WC54_RG1	0.49	0.49	4,573,830	18,419,259	35,127,408	76,647,230	0.03	0.12	0.22	0.48	245,529,335	0.87	0.76	20,855,291	0.08
WC55_RG1	0.48	0.46	3,412,268	17,700,488	35,434,499	80,026,297	0.02	0.1	0.21	0.47	266,077,535	0.85	0.75	21,162,156	0.08
WC56_RG1	0.51	0.51	3,159,046	13,698,295	27,531,839	61,691,697	0.02	0.11	0.21	0.48	206,941,174	0.85	0.73	19,834,872	0.1
WC57_RG1	0.49	0.49	3,906,114	18,977,793	37,490,975	84,267,170	0.02	0.11	0.22	0.48	270,606,089	0.86	0.75	23,147,544	0.09
WC58_RG1	0.5	0.51	5,687,767	19,393,196	37,968,902	83,119,388	0.03	0.11	0.22	0.48	271,760,020	0.86	0.74	23,843,890	0.09
WC59_RG1	0.49	0.49	2,454,806	12,872,527	29,540,710	72,779,848	0.02	0.08	0.19	0.46	247,437,811	0.85	0.73	23,467,691	0.09
WC60_RG1	0.48	0.49	3,575,642	17,278,316	33,451,248	73,387,376	0.02	0.11	0.22	0.49	242,238,890	0.84	0.72	22,933,788	0.09
WC61_RG1	0.51	0.51	2,507,030	12,349,134	27,406,995	65,625,590	0.02	0.09	0.19	0.46	229,552,257	0.84	0.72	19,639,548	0.09
WC62_RG1	0.51	0.54	1,716,112	10,056,363	30,754,615	70,736,612	0.01	0.07	0.22	0.5	231,097,359	0.85	0.69	23,121,432	0.1
WC63_RG1	0.48	0.49	4,584,522	20,410,260	41,508,804	93,922,068	0.02	0.11	0.21	0.49	302,490,031	0.87	0.75	29,134,190	0.1
WC64_RG1	0.5	0.51	3,140,033	15,685,789	31,673,433	71,433,682	0.02	0.11	0.21	0.48	231,914,950	0.87	0.75	21,483,615	0.09
WC65_RG1	0.5	0.49	2,634,615	12,100,702	28,425,064	72,080,986	0.02	0.08	0.19	0.48	236,848,361	0.87	0.73	24,561,592	0.1
WC13_RG4	0.46	0.46	755,244	2,321,481	4,846,754	9,663,279	0.04	0.14	0.28	0.57	20,516,134	0.98	0.9	1,168,703	0.06
WC13_RG5	0.46	0.46	761,662	2,334,499	4,878,180	9,728,982	0.04	0.14	0.28	0.57	20,663,306	0.98	0.9	1,176,437	0.06
WC13_RG6	0.46	0.46	758,236	2,334,665	4,873,420	9,723,231	0.04	0.14	0.28	0.57	20,650,336	0.98	0.9	1,184,263	0.06
WC11_RG2	0.46	0.46	1,359,113	3,896,390	6,365,426	10,775,486	0.08	0.22	0.36	0.61	21,455,000	0.98	0.9	1,103,277	0.05
WC11_RG3	0.46	0.46	1,362,241	3,897,923	6,366,635	10,783,411	0.08	0.22	0.36	0.61	21,498,948	0.98	0.9	1,109,645	0.05
WC11_RG4	0.46	0.46	1,354,443	3,879,618	6,345,601	10,747,244	0.08	0.22	0.36	0.61	21,424,430	0.98	0.9	1,092,469	0.05
WC11_RG5	0.46	0.46	1,367,325	3,910,175	6,402,294	10,853,395	0.08	0.22	0.36	0.61	21,654,130	0.98	0.9	1,103,901	0.05
WC11_RG6	0.46	0.46	1,366,385	3,918,952	6,413,869	10,864,465	0.08	0.22	0.36	0.61	21,673,094	0.98	0.9	1,109,096	0.05
WC12_RG2	0.46	0.46	1,406,219	3,936,642	6,429,806	10,883,461	0.08	0.22	0.36	0.61	21,386,652	0.98	0.9	1,078,148	0.05
WC12_RG3	0.46	0.46	1,405,219	3,933,690	6,421,697	10,870,362	0.08	0.22	0.36	0.61	21,390,970	0.98	0.9	1,085,802	0.05
WC12_RG4	0.46	0.46	1,405,577	3,940,132	6,441,340	10,909,000	0.08	0.22	0.36	0.61	21,462,130	0.98	0.9	1,069,917	0.05
WC12_RG5	0.46	0.46	1,416,124	3,964,389	6,495,242	11,003,527	0.08	0.22	0.36	0.61	21,682,820	0.98	0.9	1,084,839	0.05
WC12_RG6	0.46	0.46	1,420,001	3,975,418	6,505,355	11,009,881	0.08	0.22	0.36	0.61	21,664,513	0.98	0.9	1,088,346	0.05
WC8_RG2	0.46	0.46	415,845	2,266,436	4,631,586	8,889,505	0.03	0.15	0.3	0.57	18,708,560	0.98	0.9	1,000,660	0.05
WC8_RG3	0.46	0.46	414,123	2,264,406	4,629,110	8,881,939	0.03	0.15	0.3	0.57	18,718,664	0.98	0.9	1,006,270	0.05
WC8_RG4	0.46	0.46	415,923	2,277,190	4,668,454	8,958,806	0.03	0.15	0.3	0.57	18,862,042	0.98	0.9	998,428	0.05
WC8_RG5	0.46	0.46	420,012	2,295,180	4,709,989	9,035,193	0.03	0.15	0.3	0.57	19,038,071	0.98	0.9	1,007,494	0.05
WC8_RG6	0.46	0.46	420,161	2,299,902	4,709,684	9,031,792	0.03	0.15	0.3	0.57	19,020,940	0.98	0.9	1,013,944	0.05
WC1_RG2	0.46	0.46	809,567	2,510,682	5,157,682	10,215,191	0.05	0.14	0.29	0.57	21,583,607	0.98	0.9	1,211,500	0.06
WC1_RG3	0.46	0.46	803,967	2,499,615	5,135,288	10,188,650	0.04	0.14	0.29	0.57	21,565,001	0.98	0.9	1,219,669	0.06
WC1_RG4	0.46	0.46	825,463	2,539,974	5,214,098	10,313,196	0.05	0.14	0.29	0.57	21,783,270	0.98	0.9	1,209,244	0.06
WC1_RG5	0.46	0.46	831,743	2,556,164	5,241,168	10,361,659	0.05	0.14	0.29	0.57	21,916,996	0.98	0.9	1,221,541	0.06
WC1_RG6	0.46	0.46	828,300	2,551,386	5,233,117	10,356,138	0.05	0.14	0.29	0.57	21,895,880	0.98	0.9	1,227,525	0.06
WC10_RG2	0.46	0.46	1,467,787	4,537,581	7,562,744	12,862,353	0.07	0.21	0.36	0.61	25,422,132	0.98	0.9	1,343,111	0.05
WC10_RG3	0.46	0.46	1,465,601	4,530,659	7,546,023	12,834,244	0.07	0.21	0.36	0.61	25,404,575	0.98	0.9	1,354,844	0.05
WC10_RG4	0.46	0.46	1,460,724	4,530,062	7,557,957	12,850,992	0.07	0.21	0.36	0.61	25,436,473	0.98	0.9	1,333,324	0.05
WC10_RG5	0.46	0.46	1,476,707	4,570,517	7,637,446	13,000,818	0.07	0.21	0.36	0.61	25,756,452	0.98	0.9	1,348,529	0.05
WC18_RG6	0.46	0.46	828,639	2,870,866	5,580,601	10,491,747	0.05	0.16	0.31	0.58	21,994,808	0.98	0.9	1,069,284	0.05
WC15_RG2	0.46	0.46	521,354	2,388,260	5,129,341	10,083,224	0.03	0.13	0.29	0.57	21,790,022	0.98	0.9	1,106,873	0.05
WC15_RG3	0.46	0.46	519,234	2,380,258	5,115,542	10,073,711	0.03	0.13	0.29	0.57	21,786,881	0.98	0.9	1,110,662	0.05
WC15_RG4	0.46	0.46	523,549	2,399,069	5,161,136	10,155,281	0.03	0.13	0.29	0.57	21,951,860	0.98	0.9	1,101,789	0.05
WC15_RG5	0.47	0.46	526,107	2,419,206	5,206,817	10,246,325	0.03	0.13	0.29	0.57	22,160,267	0.98	0.9	1,114,594	0.05
WC15_RG6	0.47	0.46	523,668	2,417,827	5,200,772	10,232,126	0.03	0.13	0.29	0.57	22,119,316	0.98	0.9	1,119,836	0.05
WC10_RG6	0.46	0.46	1,473,915	4,573,457	7,635,028	12,984,394	0.07	0.21	0.36	0.61	25,710,570	0.98	0.9	1,354,769	0.05
WC2_RG2	0.46	0.46	573,009	2,905,203	5,945,962	11,447,348	0.03	0.15	0.3	0.57	24,089,882	0.98	0.9	1,309,112	0.05
WC7_RG2	0.46	0.46	880,704	2,651,708	5,322,132	10,414,723	0.05	0.15	0.29	0.57	21,946,289	0.98	0.9	1,217,896	0.06
WC2_RG3	0.46	0.46	575,454	2,909,950	5,950,822	11,458,089	0.03	0.15	0.3	0.57	24,130,134	0.98	0.9	1,318,571	0.05
WC7_RG3	0.46	0.46	872,232	2,639,395	5,303,200	10,386,917	0.05	0.15	0.29	0.57	21,928,523	0.98	0.9	1,224,666	0.06
WC2_RG4	0.46	0.46	572,661	2,903,663	5,942,486	11,421,862	0.03	0.15	0.3	0.57	24,031,674	0.98	0.9	1,289,595	0.05
WC7_RG4	0.46	0.46	896,802	2,684,755	5,384,092	10,523,707	0.05	0.15	0.29	0.57	22,185,314	0.98	0.9	1,220,928	0.06
WC7_RG5	0.46	0.46	909,084	2,710,341	5,438,477	10,632,586	0.05	0.15	0.29	0.57	22,416,376	0.97	0.9	1,231,028	0.05
WC7_RG6	0.46	0.46	902,942	2,703,691	5,420,850	10,600,934	0.05	0.15	0.29	0.57	22,356,048	0.98	0.9	1,237,006	0.06
WC5_RG2	0.47	0.46	1,166,336	3,358,648	5,620,828	9,684,393	0.07	0.21	0.35	0.6	19,321,369	0.98	0.9	1,001,448	0.05
WC5_RG3	0.47	0.46	1,167,148	3,364,066	5,628,477	9,696,530	0.07	0.21	0.35	0.6	19,366,636	0.98	0.9	1,009,794	0.05
WC5_RG4	0.47	0.46	1,164,076	3,352,627	5,617,406	9,677,777	0.07	0.21	0.35	0.6	19,323,606	0.98	0.9	990,911	0.05
WC5_RG5	0.47	0.46	1,167,659	3,361,149	5,639,094	9,728,474	0.07	0.21	0.35	0.6	19,452,734	0.98	0.9	1,000,764	0.05
WC5_RG6	0.47	0.46	1,173,083	3,380,666	5,669,261	9,772,583	0.07	0.21	0.35	0.6	19,517,420	0.98	0.9	1,008,343	0.05
WC18_RG2	0.46	0.46	822,797	2,834,709	5,502,264	10,339,851	0.05	0.16	0.31	0.58	21,626,032	0.98	0.91	1,054,639	0.05
WC18_RG3	0.46	0.46	822,046	2,829,418	5,495,701	10,323,801	0.05	0.16	0.31	0.58	21,638,609	0.98	0.9	1,060,924	0.05
WC18_RG4	0.46	0.46	824,354	2,851,481	5,546,766	10,421,963	0.05	0.16	0.31	0.58	21,820,464	0.98	0.91	1,052,203	0.05
WC18_RG5	0.46	0.46	829,318	2,868,968	5,583,398	10,497,710	0.05	0.16	0.31	0.58	22,015,109	0.98	0.9	1,065,519	0.05
WC2_RG5	0.46	0.46	580,181	2,937,698	6,013,583	11,552,876	0.03	0.15	0.3	0.57	24,327,347	0.98	0.9	1,308,314	0.05
WC2_RG6	0.46	0.46	578,333	2,935,680	6,008,957	11,549,727	0.03	0.15	0.3	0.57	24,311,119	0.98	0.9	1,312,186	0.05
WC16_RG2	0.47	0.46	869,065	2,972,616	5,614,392	10,263,922	0.05	0.17	0.32	0.59	21,059,258	0.98	0.9	1,046,247	0.05
WC16_RG3	0.47	0.46	869,909	2,968,239	5,611,284	10,255,217	0.05	0.17	0.32	0.59	21,074,247	0.98	0.9	1,054,549	0.05
WC16_RG4	0.47	0.46	870,700	2,993,518	5,665,383	10,362,114	0.05	0.17	0.32	0.59	21,264,736	0.98	0.9	1,045,120	0.05
WC16_RG5	0.47	0.46	878,940	3,013,755	5,705,114	10,441,706	0.05	0.17	0.32	0.59	21,460,716	0.98	0.9	1,056,519	0.05
WC16_RG6	0.47	0.46	876,260	3,010,045	5,693,363	10,417,154	0.05	0.17	0.32	0.59	21,408,672	0.98	0.9	1,059,885	0.05
WC13_RG2	0.46	0.46	741,189	2,293,739	4,797,347	9,580,153	0.04	0.14	0.28	0.57	20,347,506	0.98	0.9	1,168,857	0.06
WC13_RG3	0.46	0.46	740,232	2,295,797	4,798,339	9,590,367	0.04	0.14	0.28	0.57	20,383,880	0.98	0.9	1,177,943	0.06
WC6_RG3	0.46	0.46	1,395,926	3,868,479	6,476,692	10,985,564	0.08	0.21	0.36	0.61	21,741,443	0.98	0.9	1,113,741	0.05
WC6_RG4	0.47	0.46	1,391,445	3,858,978	6,468,973	10,977,285	0.08	0.21	0.36	0.61	21,721,994	0.98	0.9	1,094,403	0.05
WC6_RG5	0.47	0.46	1,402,856	3,879,891	6,506,658	11,058,495	0.08	0.21	0.36	0.61	21,919,012	0.98	0.9	1,104,370	0.05
WC6_RG6	0.47	0.46	1,403,532	3,891,468	6,521,519	11,071,866	0.08	0.21	0.36	0.61	21,919,846	0.98	0.9	1,111,153	0.05
WC9_RG2	0.46	0.46	1,053,376	3,742,574	6,026,467	9,830,250	0.07	0.24	0.38	0.62	19,581,800	0.97	0.9	1,030,010	0.05
WC9_RG3	0.46	0.46	1,054,687	3,753,347	6,042,713	9,855,427	0.07	0.24	0.38	0.62	19,657,201	0.97	0.9	1,039,275	0.05
WC9_RG4	0.46	0.46	1,054,098	3,747,968	6,058,178	9,890,856	0.07	0.24	0.38	0.62	19,716,972	0.97	0.9	1,025,311	0.05
WC9_RG5	0.46	0.46	1,063,771	3,772,439	6,111,627	9,989,202	0.07	0.23	0.38	0.62	19,922,650	0.97	0.89	1,039,596	0.05
WC9_RG6	0.46	0.46	1,066,273	3,783,114	6,113,978	9,992,180	0.07	0.24	0.38	0.62	19,927,167	0.97	0.9	1,043,873	0.05
WC17_RG2	0.46	0.46	878,392	3,155,850	6,108,975	11,527,763	0.04	0.16	0.31	0.58	24,114,639	0.98	0.91	1,183,854	0.05
WC17_RG3	0.46	0.46	874,457	3,142,399	6,079,430	11,476,527	0.04	0.16	0.31	0.58	24,028,920	0.98	0.9	1,184,127	0.05
WC17_RG4	0.46	0.46	883,498	3,186,961	6,191,189	11,700,233	0.04	0.16	0.31	0.58	24,500,002	0.98	0.9	1,192,518	0.05
WC17_RG5	0.46	0.46	890,558	3,213,133	6,244,333	11,796,108	0.04	0.16	0.31	0.58	24,743,737	0.98	0.9	1,207,082	0.05
WC17_RG6	0.46	0.46	887,077	3,204,513	6,213,838	11,736,917	0.04	0.16	0.31	0.58	24,588,546	0.98	0.9	1,204,948	0.05
WC4_RG2	0.46	0.46	1,060,511	3,295,860	5,524,439	9,433,711	0.07	0.21	0.36	0.61	18,557,350	0.98	0.9	977,137	0.05
WC4_RG3	0.46	0.46	1,061,537	3,299,665	5,529,794	9,444,182	0.07	0.21	0.36	0.61	18,597,687	0.98	0.9	986,107	0.05
WC4_RG4	0.46	0.46	1,059,536	3,298,666	5,535,071	9,446,521	0.07	0.21	0.36	0.61	18,599,937	0.97	0.9	968,361	0.05
WC4_RG5	0.47	0.46	1,071,051	3,314,510	5,569,618	9,516,839	0.07	0.21	0.36	0.61	18,750,261	0.97	0.9	976,176	0.05
WC4_RG6	0.47	0.46	1,070,599	3,326,435	5,583,998	9,534,146	0.07	0.21	0.36	0.61	18,770,633	0.98	0.9	981,863	0.05
WC14_RG2	0.46	0.46	479,098	2,668,317	5,704,635	11,255,831	0.02	0.13	0.29	0.57	23,990,731	0.98	0.9	1,246,996	0.05
WC14_RG3	0.46	0.46	476,996	2,657,257	5,688,415	11,235,358	0.02	0.13	0.29	0.57	23,984,987	0.98	0.9	1,254,325	0.05
WC14_RG4	0.46	0.46	482,437	2,665,211	5,708,596	11,254,147	0.02	0.13	0.29	0.57	23,994,303	0.98	0.9	1,235,617	0.05
WC14_RG5	0.46	0.46	491,592	2,693,063	5,772,861	11,376,341	0.02	0.13	0.29	0.57	24,273,992	0.98	0.9	1,254,417	0.05
WC14_RG6	0.46	0.46	486,975	2,690,271	5,757,479	11,357,571	0.02	0.13	0.29	0.57	24,226,505	0.98	0.9	1,259,437	0.05
WC3_RG1	0.46	0.46	970,190	3,107,773	5,140,397	8,688,265	0.07	0.22	0.36	0.61	17,102,986	0.98	0.9	876,351	0.05
WC6_RG1	0.46	0.46	1,367,428	3,776,194	6,286,542	10,638,917	0.08	0.22	0.36	0.61	21,007,490	0.98	0.9	1,078,416	0.05
WC9_RG1	0.46	0.46	1,013,628	3,603,781	5,761,206	9,363,805	0.07	0.24	0.38	0.62	18,634,768	0.97	0.9	992,363	0.05
WC17_RG1	0.46	0.46	855,414	3,050,759	5,889,344	11,103,025	0.04	0.16	0.31	0.58	23,195,690	0.98	0.91	1,146,973	0.05
WC4_RG1	0.46	0.46	1,032,632	3,200,043	5,347,176	9,117,141	0.07	0.21	0.36	0.61	17,909,389	0.98	0.9	951,762	0.05
WC14_RG1	0.46	0.46	456,216	2,574,235	5,499,616	10,861,245	0.02	0.13	0.29	0.57	23,146,784	0.98	0.9	1,216,269	0.05
WC1_RG1	0.46	0.46	772,857	2,413,729	4,961,417	9,842,863	0.04	0.14	0.29	0.57	20,798,803	0.98	0.91	1,182,122	0.06
WC10_RG1	0.46	0.46	1,438,762	4,426,408	7,342,968	12,463,747	0.07	0.22	0.36	0.61	24,577,313	0.98	0.9	1,310,731	0.05
WC7_RG1	0.46	0.46	831,768	2,531,087	5,085,265	9,961,324	0.05	0.15	0.29	0.57	20,995,385	0.98	0.9	1,179,226	0.06
WC5_RG1	0.46	0.46	1,140,189	3,268,985	5,443,670	9,360,457	0.07	0.21	0.35	0.6	18,632,912	0.98	0.9	974,611	0.05
WC18_RG1	0.46	0.46	796,233	2,728,147	5,283,001	9,919,201	0.05	0.16	0.31	0.58	20,715,521	0.98	0.91	1,018,505	0.05
WC15_RG1	0.46	0.46	506,763	2,302,045	4,939,893	9,712,381	0.03	0.13	0.29	0.57	20,960,369	0.98	0.9	1,072,928	0.05
WC2_RG1	0.46	0.46	558,892	2,812,003	5,732,804	11,042,396	0.03	0.15	0.3	0.57	23,203,392	0.98	0.9	1,272,204	0.05
WC16_RG1	0.46	0.46	842,189	2,865,812	5,402,750	9,870,012	0.05	0.17	0.32	0.59	20,225,084	0.98	0.91	1,014,259	0.05
WC13_RG1	0.46	0.46	706,114	2,206,727	4,617,593	9,229,461	0.04	0.13	0.28	0.56	19,607,299	0.98	0.9	1,139,372	0.06
WC11_RG1	0.46	0.46	1,327,419	3,780,119	6,151,031	10,396,447	0.08	0.22	0.36	0.61	20,646,371	0.98	0.9	1,071,742	0.05
WC12_RG1	0.46	0.46	1,373,393	3,827,083	6,222,868	10,514,570	0.08	0.22	0.36	0.61	20,610,538	0.98	0.91	1,047,276	0.05
WC8_RG1	0.46	0.46	402,234	2,180,653	4,436,063	8,513,347	0.03	0.15	0.3	0.57	17,906,482	0.98	0.9	965,468	0.05
WC3_RG2	0.46	0.46	1,002,315	3,223,012	5,357,478	9,067,227	0.07	0.22	0.36	0.61	17,888,390	0.98	0.9	910,739	0.05
WC3_RG3	0.46	0.46	1,008,272	3,236,918	5,374,420	9,091,500	0.07	0.22	0.36	0.61	17,955,988	0.98	0.9	919,718	0.05
WC3_RG4	0.47	0.46	1,004,874	3,235,326	5,382,783	9,107,792	0.07	0.22	0.36	0.61	17,978,518	0.98	0.9	905,983	0.05
WC3_RG5	0.47	0.46	1,012,204	3,249,430	5,415,023	9,174,598	0.07	0.22	0.36	0.61	18,135,937	0.98	0.9	916,274	0.05
WC3_RG6	0.47	0.46	1,013,423	3,266,197	5,435,552	9,201,613	0.07	0.22	0.36	0.61	18,173,932	0.98	0.9	920,732	0.05
WC6_RG2	0.47	0.46	1,396,877	3,874,361	6,480,694	10,992,316	0.08	0.21	0.36	0.61	21,734,633	0.98	0.9	1,106,631	0.05
