# Supplementary material for: Bone dysplasia in Hutchinson‐Gilford progeria syndrome is associated with dysregulated differentiation and function of bone cell populations
Source: Aging Cell. 2023 Jun 26;22(9):e13903. doi: 10.1111/acel.13903 (PMC10497813; doi:10.1111/acel.13903)
Supplement: Supplementary file 3 — Appendix S1. [file ACEL-22-e13903-s001.docx]

**Bone dysplasia in Hutchinson-Gilford Progeria Syndrome is associated with dysregulated differentiation and function of bone cell populations.**

Wayne A. Cabral^1^, Chris Stephan^2^, Masahiko Terajima^3^, Abhirami Thaivalappil^1^, Owen Blanchard^2^, Urraca L. Tavarez^1^, Narisu Narisu^1^, Tingfen Yan^1^, Stephen M. Wincovitch^4^, Yuki Taga^5^, Mitsuo Yamauchi^3^, Kenneth M. Kozloff^2^, Michael R. Erdos^1^, and Francis S. Collins^1#^

^1^ Molecular Genetics Section, Center for Precision Health Research, National Human Genome Research Institute, NIH, Bethesda, MD, USA

^2^ Department of Orthopedic Surgery, University of Michigan, Ann Arbor, MI, USA

^3^ Oral and Craniofacial Health Sciences, School of Dentistry, University of North Carolina, Chapel Hill, NC, USA

^4^ Cytogenetics and Microscopy Core, National Human Genome Research Institute, NIH, Bethesda, MD, USA

^5^ Nippi Research Institute of Biomatrix, Ibaraki 302-0017, Japan

**Supporting Information**

Experimental Procedures

Table S1. *Lmna*^+/+^, *Lmna*^G609G/+^, *Lmna*^G609G/G609G^ femoral bone structural parameters.

Table S2. *Lmna*^+/+^, *Lmna*^G609G/+^, *Lmna*^G609G/G609G^ femoral bone mechanical properties.

Table S3. Post-translational modification of type I collagen from *Lmna*^+/+^, *Lmna*^G609G/+^ and *Lmna*^G609G/G609G^ femoral bone.

Table S4. Cytokine profile of *Lmna*^+/+^ and *Lmna*^G609G/G609G^ femoral osteoblast-conditioned media.

Figure S1. Mice expressing *Lmna*^G609G^ have altered bone development at birth.

Figure S2. Dynamic histomorphometric analysis reveals a trend in reduced bone formation rates on endosteal femoral surfaces in *Lmna*^G609G/G609G^ mice.

Figure S3. *Lmna*^G609G/G609G^ mice have reduced osteogenic gene expression, increased adipogenic gene expression and elevated inflammatory cytokine levels.

Figure S4. Beta catenin signaling is inhibited in cultured *Lmna*^G609G/G609G^ osteoblasts.

Figure S5. Progerin is expressed in bone marrow-derived osteoclasts.

**Experimental Procedures**

**Mouse Strains and Animal Care.** The *Lmna*^G609G^ (c.1827C>T) knock-in mouse expresses the murine endogenous *Lmna* gene harboring the corresponding mutation in classic HGPS patients, and was a generous gift from Carlos-Lopez Otín, Departamento de Bioquímica y Biología Molecular, University of Oviedo (Osorio *et al.* 2011). Genotyping was performed using DNA extracted from mouse tails using the REDExtract-n-Amp tissue PCR kit (Sigma, St. Louis, MO), followed by PCR as previously reported (Osorio *et al.* 2011). Animal care and experiments were performed in accordance with a protocol approved by the NHGRI Animal Care and Use Committee.

**Cell Culture.** Osteoblasts were generated from newborn mouse calvaria or 8 week-old mouse femora by collagenase release and cultured in αMEM containing 10% fetal bovine serum, 2 mM glutamine, and 1% pen-strep at 37^o^C in 8% CO_2_. Cells undergoing osteogenic differentiation were supplemented with 50 μg/ml ascorbate and 2.5 mM β-glycerophosphate for 30 days. Cultures were collected for protein and gene expression analyses, or were fixed in 4% paraformaldehyde in PBS followed by staining with 1% alizarin red S in 2% ethanol to reveal mineral. Cells undergoing adipogenic stimulation were cultured in αMEM containing 20% fetal bovine serum, 0.1 μM dexamethasone, 1 μM insulin, 200 μM indomethacin and 250 μM isobutyl methylxanthine (Sigma, St. Louis, MO). Following fixation, cultures were stained with 0.2% Oil red-O (Sigma, St. Louis, MO) in 60% isopropanol.

Osteoclast cultures were generated from bone marrow-derived monocytes/macrophages (BMMs). Marrow cavities from dissected long bones of 8 week-old mice were flushed with ice cold αMEM using a 23-gauge needle. Cells were dispersed by passing resuspended marrow through the syringe several times and sieving through a 70 μm cell strainer. Cell suspensions were incubated in red blood cell lysis solution (Qiagen, Germantown, MD) for 3 minutes at room temperature, then centrifuged for 10 minutes at 500 x g. Pelleted leukocytes were resuspended in αMEM containing 10% fetal bovine serum, 2 mM glutamine, and 1% pen-strep, and cultured for 12 hours in 100 mm culture dishes at 37^o^C in 8% CO_2_. The following day, nonadherent cells were centrifuged at 500 x g for 10 minutes, resuspended in culture medium containing 25 ng/ml recombinant MCSF (R&D Systems, Minneapolis, MN), counted and re-plated at a density of 7 x 10^5^ cells/cm^2^. Media containing MCSF was replenished every two days until cultures reached confluency, at which point the cultures were fed growth media containing 25 ng/ml MCSF and 100 ng/ml recombinant RANKL (R&D Systems, Minneapolis, MN). In other experiments, mononuclear precursors were cultured in femoral osteoblast-conditioned media supplemented with MCSF and RANKL. Mononuclear precursors were allowed to fuse for 48-72 hours, then either harvested for gene expression and protein analyses, stained for TRAcP activity using a leukocyte acid phosphatase kit (Sigma, St Louis, MO), or analyzed by wide-field microscopy.

**Skeletal Staining.** Following euthanasia skin, viscera and eyes were carefully removed from newborn mice. Samples were fixed at room temperature in 95% ethanol for seven days and then transferred to acetone for an additional seven days to remove adipose and neural tissues. Skeletal staining proceeded for three days in staining solution consisting of 1 volume of 0.3% Alcian blue in 70% ethanol, 1 volume of 0.1% Alizarin red S in 95% ethanol, 1 volume of acetic acid and 17 volumes of 70% ethanol. Skeletal preparations were rinsed briefly in water, then destained in 1% KOH in 20% glycerol under gentle shaking at room temperature with solution changes occurring every three days for two weeks prior to imaging.

**Gene Expression Analysis.** Total RNA was extracted from cultures or femoral cortical bone tissue after removal of epiphyses and marrow from 12 wild-type (*Lmna*^+/+^, 6 males, 6 females) and 12 homozygous (*Lmna*^G609G/G609G^, 6 males, 6 females) mice at 8 weeks of age. RNA isolation used TRIzol reagent (ThermoFisher Scientific, Waltham, MA), followed by digestion for 20 minutes at 37^o^C with Dnase I (ThermoFisher), then analysis for integrity and concentration on an Agilent nucleic acid bioanalyzer (Agilent Technologies). Quantitation of murine *Lmna*, *Progerin* and *Lmnc* transcripts was performed as previously described (Cabral *et al.* 2021). For real-time RT-PCR generation of cDNA utilized a High Capacity cDNA Archive Kit (ThermoFisher Scientific, Waltham, MA) to reverse transcribe 2 μg total RNA, followed by quantitative PCR with Taqman Assays on Demand (ThermoFisher Scientific, Waltham, MA, Runx2, Mm00501584_m1; *Sp7*, Mm00504574_m1; *Col1a1*, Mm00801666_g1; *Bglpa2*, Mm03413826_mH; *Alpl*, Mm00475834_m1; *Rankl*, Mm00441906_m1; *Opg*, Mm00435454_m1; *Mepe*, Mm02525159_s1; *Dmp1*, Mm01208363_m1; *Sost*, Mm00470479_m1; *Pparg2*, Mm00440945_m1; Gapdh, Mm99999915_g1; *Hprt1*, Mm03024075_m1; *Gusb*, Mm01197698_m1; *Actb*, Mm02619580_g1; B2m, Mm00437762_m1; *Ipo8*, Mm01255158_m1). Relative expression of specific transcripts was measured in triplicate from biologic replicates and normalized to Gapdh, Hprt, Gusb, Actb, B2m, and Ipo8 transcripts.

Sequencing was performed on an Illumina NovaSeq 6000, strand-specific PolyA with 2x151 bp cycles. Paired-end RNA-seq reads were aligned to GRCm38 genome assembly using STAR (v2.7.3) with default parameters (Dobin *et al.* 2013). Expression levels of Gencode M24 genes were quantified using QoRTs (v1.3.6) (Hartley & Mullikin 2015). Phred-scaled read quality, mapping rate, and gene body coverage are provided in **Supplemental Data File 2**. Expression difference of genes between *Lmna*^G609G/G609G^ and wild-type was identified using Wald test implemented in DESeq2 with sample gender as a covariate (Love *et al.* 2014). Benjamini & Hochberg method was used to adjust for multiple testing, and genes having a fold change ≥ 1.5 or ≤ -1.5 with adjusted p value < 0.05 were considered differentially expressed (Benjamini & Hochberg 1995). The whole gene list from the results of differential gene expression comparing progeria to control samples was applied to GSEA using fgsea R package (Korotkevich *et al.* 2021). Genes were ranked by fold-change decreasingly and KEGG pathway datasets were used (**Supplemental Data File 1**). The enriched gene sets with FDR < 0.05 were plotted.

**Primary Antibodies.** Primary antibodies used in this study included rabbit anti-Lamin A/C [ab224816] (Abcam, Cambridge, UK), mouse anti-Lamin A/C [4C11], rabbit anti-beta-Actin [D6A8], rabbit anti-nonphospho b-catenin [S33/S37/T41], mouse anti-S6 [54-D2] and rabbit anti-Pparg1/2 [D69] (Cell Signaling Technology, Danvers, MA). Rabbit anti-progerin [Y288] antibody was a generous gift from K. Djabali, Technical University of Munich (McClintock *et al.* 2006).

**Wide-field Microscopy.** For osteoblast imaging, 10,000 cells were seeded into each well of eight-chamber treated culture slides (Ibidi USA Inc, Fitchburg, WI) and allowed to attach overnight. Osteoclast cultures were generated directly in the chamber slides from marrow precursors, as described in the main Methods section. Cells were fixed with 4% paraformaldehyde (Electron Microscopy Science, Hatfield, PA) in 1x PBS and permeabilized with 0.5% Triton X-100 in 1x PBS. Slides were blocked overnight with 4% BSA in 1x TBS at 4°C. Mouse anti-Lamin A/C [4c11] (Cell Signaling Technology, Danvers, MA) and rabbit anti-progerin [Y288] primary antibodies were diluted 1:200 in 4% BSA in 1x TBS and applied to slides overnight at 4°C. Progerin-specific antibody was generated and characterized as previously described (Cao *et al.* 2011). Target proteins were visualized using AlexaFluor 594 donkey anti-mouse IgG (H+L) and AlexaFluor 488 donkey anti-rabbit secondary antibodies, which were diluted 1:1,000 in 4% BSA in 1x TBS and applied for overnight at 4°C. Filamentous Actin was visualized using either AlexaFluor 488 Phalloidin or AlexaFluor 594 Phalloidin (ThermoFisher Scientific, Waltham, MA). Nuclei were stained with VECTASHIELD Antifade Mounting Medium containing DAPI (Vector Laboratories, Inc., Burlingame, CA). Wide-field images were collected using a Personal DeltaVision system (Leica Microsystems Inc, Buffalo Grove, IL) mounted on an inverted Olympus IX71 microscope with an U Apo 20x/0.75 or an N Plan Apo 60x/1,42 objective lens. All images were acquired using a pco.edge sCMOS camera with 1x1 binning and a 1024 pixels x 1024 pixels imaging field. FITC, TRITC and DAPI excitation were collected in emission filters 523/36, 679/34 and 435/48, respectively. All images were deconvolved in Leica’s SoftWoRx software package version 7.0.

**Histology.** Standard histology was performed on femora dissected from 2 month-old mice and fixed in 4% paraformaldehyde in PBS at 4°C for two days. Bones were demineralized for three weeks in 14% EDTA at 4°C under gentle shaking. Following dehydration, samples were paraffin embedded and 5 μm bone tissue sections were cut and mounted on glass slides using standard protocols. Hematoxylin and eosin (H&E) staining and TUNEL procedures were performed by standard procedures (Histoserv Inc, Germantown, MD). Additional femoral sections were stained for TRAcP enzymatic activity using a commercially available kit (Sigma, St Louis, MO).

**Histomorphometric Analyses.** Length measurements were performed on left tibiae and femora from 2 and 8 month-old mice using digital calipers. TRAcP-stained tissue sections were imaged on a Zeiss AxioScan Z.1 slide scanner (Carl Zeiss, Jena, Germany) and processed using a 20x objective and Zen Blue image analysis software. Images were analyzed for osteoclast numbers and surface area in a trabecular region of interest proximal to the distal growth plate and covering an area of 2mm^2^. For dynamic histomorphometry, 2 month-old mice received intraperitoneal injections of calcein (30mg/kg) and alizarin (30mg/kg) resuspended in saline at 10 and 3 days prior to sacrifice. Femora were dissected and fixed in 70% ethanol for 7 days, dehydrated with increasing concentrations of ethanol, cleared in xylene, embedded undecalcified in methyl methacrylate and analyzed as previously described (Sinder *et al.* 2015). In brief, fluorescent images were acquired on a Zeiss Axiovert 200M inverted microscope equipped with Apotome imaging system. Using a 10x objective multiple fluorescent images were taken of the calcein and alizarin labels around the entire cortex, including both periosteal and endosteal surfaces, and analyzed using commercially available software (Bioquant Osteo Version 17.20.10, Nashville, TN).

**Micro-Computed Tomography and Mechanical Testing.** Femora and tibiae from 8 and 32 week-old mice were dissected and cleaned of soft tissue, leaving the epiphyses intact. Bones were measured from the proximal head to the distal end of the medial and lateral condyles with a digital caliper. Left femora were scanned in water using cone beam micro-computed tomography (Skyscan 1176, Bruker, Billerica, MA). Samples were scanned at 9um isotropic voxel size with a 0.3° rotation angle, 2 frames averaged, a 0.5mm aluminum filter, with a source voltage of 50kVp, and source current of 500uA. Images were then reconstructed and calibrated to manufacturer supplied phantoms of hydroxyapatite. In the femora, regions of interest (ROI) were created for both cortical and trabecular bone regions. The cortical ROI spanned 15% of total bone length centered between the top of the distal femoral growth plate and the bottom of the third trochanter. Cortical bone was segmented using a fixed 133 grayscale threshold for 2 month animals and 137 grayscale threshold for 8 month animals. These thresholds were chosen because they were the average Otsu threshold for their groups. CTAnalysis (Bruker) was used to measure total area, bone area, marrow area, cortical thickness, endosteal perimeter, periosteal perimeter, anterior-posterior bending moment of inertia, and tissue mineral density (TMD). The trabecular ROI spanned 10% of the bone length starting at the proximal end of the distal femoral growth plate. Trabecular bone was segmented using a two-phase adaptive threshold available through CTAnalysis. Initial pre-threshold grayscale values of 109 for 2 month animals and 124 for 8 month animals were used based on the average Otsu threshold for their age groups then were more accurately segmented via the adaptive mean of minimum and maximum threshold values within a five pixel radius of the target pixels available through CTAnalysis. Parameters including bone volume fraction (BV/TV), trabecular thickness (Tb.Th), trabecular number (Tb.N), and trabecular bone mineral density (Tb.BMD) were quantified using standard stereology algorithms (CTAnalysis).

Femora were loaded to failure in four-point bending as previously described (Sinder *et al.* 2013). Testing was performed at 0.05 mm/s in the anterior-posterior direction with the posterior surface under tension using a servohydraulic testing device (MTS 858; MiniBionix; MTS Systems Corporation, Eden Prairie, MN). Force was recorded by a 50lb load cell (Sensotec, Columbus, OH) and vertical displacement by an external linear variable differential transducer (Lucas Schavitts, Hampton, VA) at 650Hz. A custom LabVIEW script was used to analyze the raw force-displacement data and calculate all four-point bending parameters.

**Biochemical Analyses.** Cells and tissue homogenates were lysed in RIPA buffer (150 mM NaCl, 1% NP-40, 0.5% Na-deoxycholate, 0.1% SDS, 50 mM Tris, pH 7.4) supplemented with protease inhibitor cocktail (Sigma-Aldrich, St. Louis, MO). Cytoplasmic and nuclear fractionation of cultures was performed using the NE-PER extraction kit (ThermoFisher Scientific, Waltham, MA). Protein concentration was determined using a BCA Protein Assay Kit (ThermoFisher Scientific, Waltham, MA). Protein samples (15 μg) were subjected to SDS-PAGE on 8% gels under denaturing conditions and electroblotted onto nitrocellulose membranes. Membranes were blocked overnight in 5% BSA in TBST, washed and incubated overnight at 4°C in TBST containing 2.5% non-fat milk and primary antibody (diluted 1:1000). After washing in TBST, membranes were incubated with corresponding IRDye infrared secondary antibody (diluted 1:10,000) (LI-COR Biosciences, Lincoln, NE). Proteins were visualized using an Odyssey Infrared Imaging System (LI-COR Biosciences, Lincoln, NE) and quantitated using Odyssey 3.0.3 software and normalized to β-actin levels. The primary antibodies used are listed in Supplemental Methods.

To quantitate circulating markers of bone formation and turnover, blood was collected from 8 and 32 week-old mice. Plasma was separated by centrifugation at 1500 x g for 15 minutes. Levels of collagen N-propeptide (PINP), tartrate resistant acid phosphatase (TRAcP5b) and type I collagen C-telopeptide (CTX-I) were measured using a Rat/Mouse PINP EIA, MouseTRAP^TM^ ELISA and RatLaps kits (Immunodiagnostic Systems, Tyne & Wear, UK), respectively. Cytokine levels in plasma and osteoblast-conditioned media were determined using the Quantibody Mouse Cytokine Array Q4000 (RayBiotech, Peachtree Corners, GA).

Marrow fat quantitation was performed using the L-type Triglyceride M assay (Wako Diagnostics, Mountain View, CA). Femoral bone marrow was flushed with PBS, followed by centrifugation. Pellets were dissolved in hexane/2-propanol (3:2), homogenized, then allowed to rest for 5 minutes to encourage phase separation. The upper layer was transferred to a new tube and left overnight with lid open to allow evaporation of the solvent, followed by reconstitution with 200 μl of 2-propanol for measurement.

**Tissue Collagen Composition, Post-translational Modifications and Cross-links.** Analysis of tissue-derived collagens was performed using femora from 2 month-old mice. After marrow was flushed with cold PBS, bone samples were pulverized in liquid N_2_, demineralized with EDTA at 4°C for 2 weeks, and lyophilized. Two mg of the dried samples were reduced with standardized NaB^3^H_4_, hydrolyzed with 6N HCl and subjected to amino acid and cross-link analyses as described (Yamauchi *et al.* 2018). Collagen composition was estimated as hydroxyproline/1,000 amino acids. Collagen lysine hydroxylation was calculated as hydroxylysine (Hyl)/hydroxyproline (Hyp) x 300 (i.e. ∼300 residues of Hyp/collagen).

Lysine modifications (hydroxylation and glycosylation) and prolyl-3-hydroxylation at specific molecular loci were analyzed by mass spectrometry as reported (Terajima *et al.* 2016). The reducible cross-links, i.e. dehydro-dihydroxylysinonorleucine (deH-DHLNL)/its ketoamine form, dehydro-hydroxylysinonorleucine (deH-HLNL) and dehydro-histidinohydroxymerodesmosine (deH-HHMD, were measured as their reduced forms, DHLNL, HLNL and HHMD, respectively. The non-reducible cross-links, pyridinoline (Pyr) and deoxypyridinoline (d-Pyr) were also simultaneously analyzed. All cross-links were quantified as moles/mole of collagen.

**Table S1. *Lmna*^+/+^, *Lmna*^G609G/+^, *Lmna*^G609G/G609G^ femoral bone structural parameters.**

|  |  | | | |  | |  | |  | |  | |  |  | | |
| --- | --- | --- | --- | --- | --- | --- | --- | --- | --- | --- | --- | --- | --- | --- | --- | --- |
|  | | |  |  | | **2 Months** | |  | |  | |  | | | **8 months** | |
|  | | |  |  | |  | |  | |  | |  | |  | | |
|  | | |  |  | |  | |  | |  | |  | | | |  |
|  | | |  | ***Lmna*^+/+^** | | ***Lmna*^G609G/+^** | | ***Lmna*^G609G/G609G^** | |  | | ***Lmna*^+/+^** | | | | ***Lmna*^G609G/+^** |
|  | | |  |  | |  | |  | |  | |  | | | |  |
|  | | |  |  | |  | |  | |  | |  | | | |  |
| Trabecular Number (Tb N) | | | Males | 2.413 ± 0.372 | | 2.107 ± 0.222* | | 1.762 ± 0.295*** | |  | | 1.223 ± 0.367 | | | | 1.178 ± 0.225 |
|  | | | Females | 1.402 ± 0.257 | | 1.336 ± 0.273 | | 1.211 ± 0.268 | |  | | 0.474 ± 0.209 | | | | 0.315 ± 0.075* |
|  | | | Combined | 1.883 ± 0.602 | | 1.742 ± 0.463 | | 1.486 ± 0.394* | |  | | 0.829 ± 0.479 | | | | 0.747 ± 0.472 |
|  | | |  |  | |  | |  | |  | |  | | | |  |
| Trabecular Thickness (Tb Th, mm) | | | Males | 0.054 ± 0.003 | | 0.054 ± 0.003 | | 0.048 ± 0.003*** | |  | | 0.059 ± 0.006 | | | | 0.054 ± 0.004* |
|  | | | Females | 0.051 ± 0.002 | | 0.050 ± 0.001 | | 0.046 ± 0.001*** | |  | | 0.055 ± 0.006 | | | | 0.050 ± 0.003* |
|  | | | Combined | 0.052 ± 0.003 | | 0.052 ± 0.003 | | 0.047 ± 0.002*** | |  | | 0.057 ± 0.006 | | | | 0.052 ± 0.004** |
|  | | |  |  | |  | |  | |  | |  | | | |  |
| Trabecular Bone Volume Fraction (Tb BV/TV, %) | | | Males | 12.968 ± 1.972 | | 11.387 ± 1.328* | | 8.483 ± 1.742*** | |  | | 7.290 ± 2.783 | | | | 6.369 ± 1.588 |
|  | | | Females | 7.099 ± 1.331 | | 6.642 ± 1.461 | | 5.623 ± 1.295* | |  | | 2.706 ± 1.470 | | | | 1.560 ± 0.369* |
|  | | | Combined | 9.894 ± 3.414 | | 9.139 ± 2.785 | | 7.053 ± 2.094** | |  | | 4.877 ± 3.171 | | | | 3.964 ± 2.710 |
|  | | |  |  | |  | |  | |  | |  | | | |  |
| Trabecular Mineral Density (Tb BMD, g/cm^3^) | | | Males | 0.181 ± 0.020 | | 0.165 ± 0.013 | | 0.132 ± 0.026*** | |  | | 0.141 ± 0.032 | | | | 0.134 ± 0.023 |
|  | | | Females | 0.110 ± 0.022 | | 0.102 ± 0.023 | | 0.091 ± 0.016* | |  | | 0.089 ± 0.017 | | | | 0.064 ± 0.005*** |
|  | | | Combined | 0.144 ± 0.042 | | 0.135 ± 0.037 | | 0.112 ± 0.029** | |  | | 0.114 ± 0.036 | | | | 0.099 ± 0.039 |
|  | | |  |  | |  | |  | |  | |  | | | |  |
| Structural Model Index (SMI) | | | Males | 2.063 ± 0.149 | | 2.146 ± 0.095 | | 2.302 ± 0.153** | |  | | 2.295 ± 0.314 | | | | 2.299 ± 0.175 |
|  | | | Females | 2.333 ± 0.081 | | 2.336 ± 0.108 | | 2.429 ± 0.146 | |  | | 2.738 ± 0.226 | | | | 2.933 ± 0.159* |
|  | | | Combined | 2.205 ± 0.180 | | 2.236 ± 0.138 | | 2.366 ± 0.159** | |  | | 2.528 ± 0.348 | | | | 2.616 ± 0.364 |
|  | | |  |  | |  | |  | |  | |  | | | |  |
|  | | |  |  | |  | |  | |  | |  | | | |  |
| Cortical Tissue Mineral Density (Ct TMD, g/cm^3^ | | | Males | 0.945 ± 0.019 | | 0.946 ± 0.010 | | 0.927 ± 0.015* | |  | | 0.970 ± 0.009 | | | | 0.961 ± 0.012 |
|  | | | Females | 0.925 ± 0.018 | | 0.925 ± 0.019 | | 0.908 ± 0.014* | |  | | 0.993 ± 0.007 | | | | 0.970 ± 0.006*** |
|  | | | Combined | 0.934 ± 0.021 | | 0.936 ± 0.018 | | 0.918 ± 0.017* | |  | | 0.982 ± 0.014 | | | | 0.966 ± 0.011*** |
|  | | |  |  | |  | |  | |  | |  | | | |  |
| Cross-sectional Tissue Area (Tt Ar, mm^2^) | | | Males | 0.956 ± 0.096 | | 0.944 ± 0.046 | | 0.826 ± 0.086** | |  | | 1.092 ± 0.097 | | | | 0.994 ± 0.052* |
|  | | | Females | 0.805 ± 0.047 | | 0.801 ± 0.047 | | 0.702 ± 0.036*** | |  | | 1.049 ± 0.054 | | | | 0.948 ± 0.069** |
|  | | | Combined | 0.877 ± 0.106 | | 0.876 ± 0.086 | | 0.764 ± 0.089** | |  | | 1.069 ± 0.078 | | | | 0.971 ± 0.064*** |
|  | | |  |  | |  | |  | |  | |  | | | |  |
| Cortical Cross-sectional Thickness (Ct Th, mm) | | | Males | 0.169 ± 0.012 | | 0.167 ± 0.007 | | 0.148 ± 0.008*** | |  | | 0.186 ± 0.009 | | | | 0.171 ± 0.008** |
|  | | | Females | 0.152 ± 0.009 | | 0.150 ± 0.009 | | 0.136 ± 0.007*** | |  | | 0.206 ± 0.006 | | | | 0.182 ± 0.008*** |
|  | | | Combined | 0.160 ± 0.014 | | 0.159 ± 0.012 | | 0.142 ± 0.009*** | |  | | 0.197 ± 0.013 | | | | 0.176 ± 0.009*** |
|  | | |  |  | |  | |  | |  | |  | | | |  |
| Mean Marrow Area (MaAr, mm^2^) | | | Males | 1.214 ± 0.090 | | 1.236 ± 0.069 | | 1.201 ± 0.175 | |  | | 1.265 ± 0.155 | | | | 1.263 ± 0.105 |
|  | | | Females | 1.086 ± 0.080 | | 1.110 ± 0.069 | | 1.025 ± 0.063 | |  | | 0.953 ± 0.054 | | | | 1.043 ± 0.085* |
|  | | | Combined | 1.147 ± 0.106 | | 1.177 ± 0.093 | | 1.113 ± 0.156 | |  | | 1.101 ± 0.194 | | | | 1.153 ± 0.146 |
|  | | |  |  | |  | |  | |  | |  | | | |  |
| Cortical Moment of Inertia (MOI, mm^4^) | | | Males | 0.160 ± 0.030 | | 0.161 ± 0.017 | | 0.130 ± 0.026* | |  | | 0.195 ± 0.043 | | | | 0.175 ± 0.023 |
|  | | | Females | 0.118 ± 0.013 | | 0.120 ± 0.012 | | 0.096 ± 0.011*** | |  | | 0.171 ± 0.018 | | | | 0.158 ± 0.023 |
|  | | | Combined | 0.138 ± 0.031 | | 0.142 ± 0.026 | | 0.113 ± 0.026* | |  | | 0.182 ± 0.034 | | | | 0.166 ± 0.024 |
|  | |  | |  | |  | |  | |  | |  | | | |  |

Males, 2-month *Lmna*^+/+^ n=10, *Lmna*^G609G/+^ n=10, *Lmna*^G609G/G609G^ n=9; 8-month *Lmna*^+/+^ n=9, *Lmna*^G609G/+^ n=10

Females, 2-month *Lmna*^+/+^ n=11, *Lmna*^G609G/+^ n=9, *Lmna*^G609G/G609G^ n=9; 8-month *Lmna*^+/+^ n=10, *Lmna*^G609G/+^ n=10

*p < 0.05, **p < 0.01, ***p < 0.001 versus *Lmna*^+/+^ littermates

**Table S2. *Lmna*^+/+^, *Lmna*^G609G/+^, *Lmna*^G609G/G609G^ femoral bone mechanical properties.**

|  |  | | |  | |  | |  | |  | |  |  | | |
| --- | --- | --- | --- | --- | --- | --- | --- | --- | --- | --- | --- | --- | --- | --- | --- |
|  | |  |  | | **2 Months** | |  | |  | |  | | | **8 months** | |
|  | |  |  | |  | |  | |  | |  | |  | | |
|  | |  |  | |  | |  | |  | |  | | | |  |
|  | |  | ***Lmna*^+/+^** | | ***Lmna*^G609G/+^** | | ***Lmna*^G609G/G609G^** | |  | | ***Lmna*^+/+^** | | | | ***Lmna*^G609G/+^** |
|  | |  |  | |  | |  | |  | |  | | | |  |
|  | |  |  | |  | |  | |  | |  | | | |  |
| Elastic Properties | |  |  | |  | |  | |  | |  | | | |  |
|  | |  |  | |  | |  | |  | |  | | | |  |
| Yield Load (N) | | Males | 17.58 ± 3.20 | | 18.45 ± 3.05 | | 14.10 ± 2.12* | |  | | 14.21 ± 1.84 | | | | 13.58 ± 2.86 |
|  | | Females | 14.55 ± 3.58 | | 15.06 ± 1.81 | | 12.00 ± 1.44* | |  | | 15.67 ± 3.82 | | | | 14.40 ± 2.09 |
|  | | Combined | 15.99 ± 3.66 | | 16.85 ± 3.02 | | 13.05 ± 2.06** | |  | | 14.98 ± 3.06 | | | | 13.99 ± 2.48 |
|  | |  |  | |  | |  | |  | |  | | | |  |
| Yield Displacement (mm) | | Males | 0.465 ± 0.071 | | 0.514 ± 0.129 | | 0.366 ± 0.134* | |  | | 0.317 ± 0.111 | | | | 0.409 ± 0.070* |
|  | | Females | 0.475 ± 0.115 | | 0.576 ± 0.068* | | 0.496 ± 0.119 | |  | | 0.335 ± 0.127 | | | | 0.346 ± 0.155 |
|  | | Combined | 0.470 ± 0.094 | | 0.544 ± 0.107 | | 0.431 ± 0.140 | |  | | 0.326 ± 0.116 | | | | 0.378 ± 0.121 |
|  | |  |  | |  | |  | |  | |  | | | |  |
| Stiffness (N/mm) | | Males | 122.36 ± 29.92 | | 115.64 ± 30.55 | | 121.33 ± 16.74 | |  | | 167.39 ± 29.84 | | | | 169.36 ± 35.71 |
|  | | Females | 106.80 ± 31.67 | | 110.93 ± 39.42 | | 90.23 ± 17.99 | |  | | 245.96 ± 32.98 | | | | 189.64 ± 30.13*** |
|  | | Combined | 114.21 ± 31.11 | | 113.41 ± 34.10 | | 105.78 ± 23.24 | |  | | 208.74 ± 50.64 | | | | 179.50 ± 33.80* |
|  | |  |  | |  | |  | |  | |  | | | |  |
| Work to Yield (N-mm) | | Males | 2.18 ± 0.79 | | 2.46 ± 1.03 | | 1.30 ± 0.40** | |  | | 1.09 ± 0.21 | | | | 1.18 ± 0.32 |
|  | | Females | 1.74 ± 0.71 | | 1.91 ± 0.66 | | 1.41 ± 0.46 | |  | | 1.06 ± 0.46 | | | | 1.05 ± 0.35 |
|  | | Combined | 1.95 ± 0.76 | | 2.20 ± 0.89 | | 1.35 ± 0.42** | |  | | 1.07 ± 0.35 | | | | 1.11 ± 0.33 |
|  | |  |  | |  | |  | |  | |  | | | |  |
| Plastic Properties | |  |  | |  | |  | |  | |  | | | |  |
|  | |  |  | |  | |  | |  | |  | | | |  |
| Fracture Load (N) | | Males | 12.92 ± 2.95 | | 12.91 ± 4.38 | | 13.12 ± 4.42 | |  | | 12.24 ± 2.76 | | | | 14.57 ± 5.88 |
|  | | Females | 12.25 ± 3.71 | | 12.94 ± 5.43 | | 9.74 ± 3.68 | |  | | 24.78 ± 4.11 | | | | 16.53 ± 5.27* |
|  | | Combined | 12.57 ± 3.30 | | 12.92 ± 4.76 | | 11.43 ± 4.31 | |  | | 18.84 ± 7.30 | | | | 15.55 ± 5.53 |
|  | |  |  | |  | |  | |  | |  | | | |  |
| Post-Yield Displacement (PYD, mm) | | Males | 0.668 ± 0.293 | | 0.708 ± 0.361 | | 0.458 ± 0.271 | |  | | 0.569 ± 0.346 | | | | 0.277 ± 0.367 |
|  | | Females | 0.689 ± 0.402 | | 0.643 ± 0.251 | | 0.534 ± 0.189 | |  | | 0.305 ± 0.091 | | | | 0.302 ± 0.250 |
|  | | Combined | 0.679 ± 0.346 | | 0.677 ± 0.307 | | 0.496 ± 0.230 | |  | | 0.430 ± 0.275 | | | | 0.289 ± 0.306 |
|  | |  |  | |  | |  | |  | |  | | | |  |
| Post-Yield Work (N-mm) | | Males | 11.21 ± 2.95 | | 11.23 ± 4.07 | | 6.85 ± 3.55** | |  | | 9.81 ± 5.68 | | | | 3.99 ± 4.95* |
|  | | Females | 9.23 ± 3.96 | | 9.88 ± 4.82 | | 6.68 ± 3.05 | |  | | 6.11 ± 2.80 | | | | 4.78 ± 3.30 |
|  | | Combined | 10.17 ± 3.58 | | 10.59 ± 4.37 | | 6.76 ± 3.22** | |  | | 7.86 ± 4.67 | | | | 4.39 ± 4.11* |
|  | |  |  | |  | |  | |  | |  | | | |  |
| Total Properties | |  |  | |  | |  | |  | |  | | | |  |
|  | |  |  | |  | |  | |  | |  | | | |  |
| Maximum Load (N) | | Males | 21.23 ± 3.26 | | 21.22 ± 2.00 | | 18.62 ± 1.77* | |  | | 21.13 ± 5.28 | | | | 18.47 ± 3.63 |
|  | | Females | 17.82 ± 3.50 | | 18.53 ± 3.26 | | 15.38 ± 1.83* | |  | | 26.48 ± 4.11 | | | | 19.86 ± 3.17*** |
|  | | Combined | 19.44 ± 3.74 | | 19.95 ± 2.93 | | 17.00 ± 2.41* | |  | | 23.95 ± 5.33 | | | | 19.17 ± 3.39** |
|  | |  |  | |  | |  | |  | |  | | | |  |
| Total Work (N-mm) | | Males | 13.39 ± 2.90 | | 13.69 ± 3.48 | | 8.14 ± 3.21** | |  | | 10.89 ± 5.69 | | | | 5.17 ± 4.91* |
|  | | Females | 10.31 ± 4.59 | | 11.80 ± 4.43 | | 8.08 ± 2.73 | |  | | 7.70 ± 2.15 | | | | 5.83 ± 3.48 |
|  | | Combined | 11.78 ± 4.10 | | 12.79 ± 3.97 | | 8.11 ± 2.89** | |  | | 9.22 ± 4.40 | | | | 5.50 ± 4.16* |
|  | |  |  | |  | |  | |  | |  | | | |  |

Males, 2-month *Lmna*^+/+^ n=10, *Lmna*^G609G/+^ n=10, *Lmna*^G609G/G609G^ n=9; 8-month *Lmna*^+/+^ n=9, *Lmna*^G609G/+^ n=10

Females, 2-month *Lmna*^+/+^ n=11, *Lmna*^G609G/+^ n=9, *Lmna*^G609G/G609G^ n=9; 8-month *Lmna*^+/+^ n=10, *Lmna*^G609G/+^ n=10

*p < 0.05, **p < 0.01, ***p < 0.001 versus *Lmna*^+/+^ littermates

**Table S3. Post-translational modification of type I collagen from *Lmna*^+/+^, *Lmna*^G609G/+^ and *Lmna*^G609G/G609G^ femoral bone.**

|  |  |  |  |  |
| --- | --- | --- | --- | --- |
|  |  |  | **Site Occupancy (%)** |  |
|  |  |  |  |  |
|  |  |  |  |  |
|  |  | ***Lmna*^+/+^** | ***Lmna*^G609G/+^** | ***Lmna*^G609G/G609G^** |
|  |  |  |  |  |
|  |  |  |  |  |
| α1(I) Lys-87 | Lys | 7.0 ± 4.1 | 13.6 ± 6.7 | 14.4 ± 6.9 |
|  | Hyl | 26.5 ± 16.9 | 27.0 ± 4.3 | 36.0 ± 11.1 |
|  | G-Hyl | 19.8 ± 5.0 | 17.8 ± 2.7 | 14.7 ± 3.6 |
|  | GG-Hyl | 46.7 ± 11.5 | 41.5 ± 8.1 | 34.9 ± 8.4 |
|  |  |  |  |  |
| α1(I) Lys-99 | Lys | 55.5 ± 3.1 | 56.0 ± 0.8 | 57.9 ± 1.8 |
|  | Hyl | 35.5 ± 2.4 | 35.1 ± 0.8 | 33.6 ± 1.2 |
|  | G-Hyl | 7.9 ± 0.7 | 7.8 ± 0.1 | 7.4 ± 0.7 |
|  | GG-Hyl | 1.1 ± 0.2 | 1.1 ± 0.1 | 1.2 ± 0.1 |
|  |  |  |  |  |
| α1(I) Lys-174 | Lys | 22.9 ± 3.2 | 23.6 ± 0.8 | 26.6 ± 2.8 |
|  | Hyl | 68.1 ± 2.4 | 67.3 ± 1.0 | 65.0 ± 2.1 |
|  | G-Hyl | 7.6 ± 0.9 | 7.7 ± 0.1 | 7.1 ± 0.8 |
|  | GG-Hyl | 1.4 ± 0.0 | 1.4 ± 0.1 | 1.3 ± 0.1 |
|  |  |  |  |  |
| α1(I) Lys-219 | Lys | 70.0 ± 1.5 | 70.8 ± 0.6 | 71.5 ± 0.8 |
|  | Hyl | 30.0 ± 1.5 | 29.2 ± 0.6 | 28.5 ± 0.8 |
|  |  |  |  |  |
| α1(I) Lys-564 | Lys | 40.3 ± 3.3 | 40.1 ± 1.8 | 42.3 ± 2.7 |
|  | Hyl | 51.7 ± 3.1 | 51.9 ± 1.6 | 50.3 ± 2.5 |
|  | G-Hyl | 6.4 ± 0.4 | 6.3 ± 0.4 | 5.8 ± 0.4 |
|  | GG-Hyl | 1.6 ± 0.1 | 1.6 ± 0.1 | 1.6 ± 0.1 |
|  |  |  |  |  |
| α1(I) Lys-918/930 | Lys + Lys | 5.1 ± 1.3 | 5.5 ± 0.9 | 5.2 ± 0.4 |
|  | Lys + Hyl | 13.7 ± 2.9 | 14.6 ± 1.5 | 13.5 ± 0.8 |
|  | Hyl + Hyl | 81.1 ± 4.2 | 79.9 ± 2.3 | 81.2 ± 1.2 |
|  |  |  |  |  |
| α2(I) Lys-87 | Lys | 9.5 ± 1.7 | 9.6 ± 1.4 | 8.4 ± 2.6 |
|  | Hyl | 90.5 ± 1.7 | 90.4 ± 1.4 | 91.6 ± 2.6 |
|  |  |  |  |  |
| α2(I) Lys-174 | Lys | 12.7 ± 1.2 | 12.4 ± 1.2 | 12.3 ± 2.8 |
|  | Hyl | 21.6 ± 10.1 | 22.5 ± 4.6 | 30.2 ± 8.9 |
|  | G-Hyl | 59.4 ± 9.3 | 58.6 ± 3.9 | 51.7 ± 6.7 |
|  | GG-Hyl | 6.3 ± 0.3 | 6.5 ± 0.3 | 5.8 ± 0.4 |
|  |  |  |  |  |
| α2(I) Lys-219 | Lys | 11.9 ± 2.9 | 12.4 ± 1.0 | 14.6 ± 2.3 |
|  | Hyl | 82.2 ± 3.0 | 81.5 ± 1.2 | 80.2 ± 2.2 |
|  | G-Hyl | 3.2 ± 0.2 | 3.3 ± 0.1 | 2.8 ± 0.3 |
|  | GG-Hyl | 2.6 ± 0.2 | 2.8 ± 0.1 | 2.4 ± 0.2 |
|  |  |  |  |  |
| α2(I) Lys-933 | Lys | 1.4 ± 0.7 | 1.1 ± 0.2 | 1.0 ± 0.7 |
|  | Hyl | 97.8 ± 0.2 | 97.9 ± 0.3 | 97.9 ± 0.6 |
|  | G-Hyl | 0.8 ± 0.6 | 1.0 ± 0.3 | 1.1 ± 0.4 |
|  | GG-Hyl | 0.0 ± 0.0 | 0.0 ± 0.0 | 0.0 ± 0.0 |
|  |  |  |  |  |
| α1(I) Lys-9^N^ | Lys | 9.2 ± 5.1 | 8.6 ± 2.9 | 10.6 ± 2.7 |
|  | Hyl | 90.8 ± 5.1 | 91.4 ± 2.9 | 89.4 ± 2.7 |
|  |  |  |  |  |
| α1(I) Lys-16^C^ | Lys | 14.7 ± 6.2 | 13.4 ± 2.1 | 14.4 ± 2.3 |
|  | Hyl | 85.3 ± 6.2 | 86.6 ± 2.1 | 85.6 ± 2.3 |
|  |  |  |  |  |
| α2(I) Lys-5^N^ | Lys | 29.9 ± 5.1 | 25.4 ± 28.0 | 20.9 ± 8.7 |
|  | Hyl | 70.1 ± 5.1 | 74.6 ± 28.0 | 79.1 ± 8.7 |
|  |  |  |  |  |
| α1(I) Pro-986 | Pro | 1.7 ± 0.6 | 1.8 ± 0.2 | 2.0 ± 0.4 |
|  | 3-Hyp | 98.3 ± 0.6 | 98.2 ± 0.2 | 98.0 ± 0.4 |
|  |  |  |  |  |
| α1(I) Pro-707 | Pro | 82.3 ± 7.7 | 81.4 ± 3.1 | 78.3 ± 5.9 |
|  | 3-Hyp | 17.7 ± 7.7 | 18.6 ± 3.1 | 21.7 ± 5.9 |
|  |  |  |  |  |
| α2(I) Pro-707 | Pro | 79.0 ± 8.6 | 77.7 ± 4.4 | 72.1 ± 8.4 |
|  | 3-Hyp | 21.0 ± 8.6 | 22.3 ± 4.4 | 27.9 ± 8.4 |
|  |  |  |  |  |

Lysine hydroxylation/glycosylation (%) represents the relative levels of Lys, Hyl, G-Hyl, and GG-Hyl (Lys + Hyl + G-Hyl + GG-Hyl = 100%). Pro 3-hydroxylation (%) represents the relative levels of proline and 3-Hydroxyproline (Pro + 3-Hyp = 100%). Lys, lysine; Hyl, hydroxylysine; G-, galactosyl-; GG-, glucosylgalactosyl; Pro, proline; 3-Hyp, 3-hydroxyproline; N, N-telopeptide; C, C-telopeptide

**Table S4. Cytokine profile of *Lmna*^+/+^ and *Lmna*^G609G/G609G^ femoral osteoblast-conditioned media.**

|  |  |  |  |  |  |  |  |  |
| --- | --- | --- | --- | --- | --- | --- | --- | --- |
| **Target** | **Uniprot** | **Gene ID** | ***Lmna*^+/+^** | ***Lmna*^G609G/G609G^** | **P value** |  |  |  |
|  |  |  | **(pg/ml adj*)** | **(pg/ml adj*)** |  |  |  |  |
|  |  |  |  |  |  |  |  |  |
|  |  |  |  |  |  |  |  |  |
| CXCL16 | Q8BSU2 | 66102 | 338.7 ± 19.7 | 740.3 ± 60.8 | 1.55e-05 |  |  |  |
| MMP3 | P28862 | 17392 | 9,573.7 ± 1,513.2 | 28,020.5 ± 2545.9 | 1.63e-05 |  |  |  |
| PARN | Q8VDG3 | 74108 | 44.9 ± 12.3 | 152.6 ± 12.4 | 1.72e-05 |  |  |  |
| CDH3 | P10287 | 12560 | 7,56.2 ± 166.9 | 2,459.7 ± 255.7 | 3.09e-05 |  |  |  |
| PTX3 | P48759 | 19288 | 3,092.0 ± 1126.9 | 9,842.5 ± 466.1 | 3.23e-05 |  |  |  |
| MMP2 | P33434 | 17390 | 7,320.2 ± 1,176.7 | 15,991.4 ± 1,072.4 | 3.54e-05 |  |  |  |
| TNFRSF13C | Q9D8D0 | 72049 | 125.7 ± 28.4 | 509.4 ± 72.7 | 6.36e-05 |  |  |  |
| CXCL1 | P12850 | 14825 | 903.1 ± 83.7 | 1,363.8 ± 44.9 | 6.88e-05 |  |  |  |
| DCN | P28654 | 13179 | 7,279.0 ± 1,199.7 | 22,717.6 ± 3,006.0 | 7.57e-05 |  |  |  |
| CST3 | P21460 | 13010 | 4,968.5 ± 825.7 | 14,889.0 ± 1,915.2 | 7.69e-05 |  |  |  |
| LGALS3 | P16110 | 16854 | 673.8 ± 45.7 | 2,060.9 ± 301.7 | 9.94e-05 |  |  |  |
| CXCL5 | P50228 | 20311 | 411.9 ± 30.4 | 695.7 ± 56.3 | 1.14e-04 |  |  |  |
| SPP1 | P10923 | 20750 | 8,603.8 ± 1,468.6 | 23,804.5 ± 3,239.9 | 1.41e-04 |  |  |  |
| TNFRSF11B | O08712 | 18383 | 7,833.6 ± 1,533.5 | 26,311.7 ± 4,127.4 | 1.56e-04 |  |  |  |
| IGF1 | P05017 | 16000 | 354.4 ± 26.8 | 930.8 ± 135.9 | 1.63e-04 |  |  |  |
| CLU | Q06890 | 12759 | 6,910.2 ± 1,904.5 | 17,950.8 ± 2,060.4 | 2.23e-04 |  |  |  |
| IL7 | P10168 | 16196 | 213.5 ± 29.0 | 495.3 ± 67.6 | 2.57e-04 |  |  |  |
| IGFBP6 | P47880 | 16012 | 8,445.3 ± 1,908.5 | 912.6 ± 555.0 | 2.74e-04 |  |  |  |
| CXCL12 | P40224 | 20315 | 310.6 ± 130.1 | 1,464.5 ± 299.1 | 3.99e-04 |  |  |  |
| IL7R | P16872 | 16197 | 1,147.4 ± 271.8 | 3,947.0 ± 750.6 | 4.19e-04 |  |  |  |
| CCL3 | P10855 | 20302 | 130.4 ± 18.4 | 288.1 ± 42.5 | 4.94e-05 |  |  |  |
| CCL11 | P48298 | 20292 | 20.4 ± 5.8 | 49.8 ± 6.5 | 5.14e-04 |  |  |  |
| IGFBP5 | Q07079 | 16011 | 6,127.5 ± 1,437.1 | 14,305.6 ± 2,029.3 | 5.92e-04 |  |  |  |
| TNFRSF1A | P25118 | 21937 | 186.9 ± 10.9 | 355.3 ± 52.1 | 7.28e-04 |  |  |  |
| GREM1 | O70326 | 26585 | 908.5 ± 21.4 | 2,597.7 ± 574.6 | 1.08e-03 |  |  |  |
| TNFSF10 | P50592 | 22035 | 990.6 ± 223.0 | 3,682.9 ± 895.5 | 1.12e-03 |  |  |  |
| CCL9 | P51670 | 20308 | 1,432.8 ± 250.4 | 3,985.6 ± 845.8 | 1.16e-03 |  |  |  |
| IGFBP2 | P47877 | 16008 | 141.1 ± 15.9 | 390.2 ± 94.1 | 1.98e-03 |  |  |  |
| IL33 | Q8BVZ5 | 77125 | 97.9 ± 48.8 | 437.7 ± 126.5 | 2.43e-03 |  |  |  |
| MMP9 | P41245 | 17395 | 1,287.9 ± 158.2 | 3,255.7 ± 782.1 | 2.63e-03 |  |  |  |
| IGFBP3 | P47878 | 16009 | 276.5 ± 87.7 | 47.4 ± 40.1 | 3.15e-03 |  |  |  |
| TNFSF12 | O54907 | 21944 | 596.1 ± 179.6 | 78.1 ± 156.2 | 4.81e-03 |  |  |  |
| LEP | P41160 | 16846 | 37.7 ± 6.3 | 91.0 ± 23.8 | 4.97e-03 |  |  |  |
| TNFRSF1B | P25119 | 21938 | 152.2 ± 37.9 | 243.7 ± 19.2 | 5.05e-03 |  |  |  |
| VEGFA | Q00731 | 22339 | 6,503.0 ± 513.2 | 9,990.3 ± 1,571.2 | 5.56e-03 |  |  |  |
| CCL20 | O89093 | 20297 | 117.2 ± 32.6 | 38.7 ± 19.0 | 5.99e-03 |  |  |  |
| PDGFA | P20033 | 18590 | 139.3 ± 28.7 | 435.7 ± 140.5 | 6.12e-03 |  |  |  |
| FGF2 | P15655 | 14173 | 33.0 ± 8.6 | 99.3 ± 31.3 | 6.46e-03 |  |  |  |
| ADIPOQ | Q60994 | 11450 | 573.3 ± 114.5 | 1,055.0 213.2 | 7.27e-03 |  |  |  |
| CDH1 | P09803 | 12550 | 11.8 ± 5.9 | 0.3 ± 0.6 | 8.12e-03 |  |  |  |
| IL17A | Q62386 | 16171 | 1.3 ± 1.1 | 14.7 ± 7.0 | 8.90e-03 |  |  |  |
| GPNMB | Q99P91 | 93695 | 2,314.6 ± 252.4 | 3,174.6 ± 392.5 | 1.03e-02 |  |  |  |
| IFNGR1 | P15261 | 15979 | 7.2 ± 4.1 | ND | 1.17e-02 |  |  |  |
| FAS | P25446 | 14102 | 16.8 ± 8.0 | 2.2 ± 2.1 | 1.19e-02 |  |  |  |
| IL12B | P43432 | 16160 | 456.1 ± 108.3 | 1,248.3 ± 440.8 | 1.30e-02 |  |  |  |
| CD48 | P18181 | 12506 | 346.6 ± 61.5 | 491.1 ± 56.2 | 1.34e-02 |  |  |  |
| POSTN | Q62009 | 50706 | 23,109.6 ± 1,428.7 | 52,374.3 ± 18,447.7 | 1.95e-02 |  |  |  |
| CCL12 | Q62401 | 20293 | 189.0 ± 21.1 | 143.3 ± 21.9 | 2.38e-02 |  |  |  |
| TGFB1 | P04202 | 21803 | 28,445.4 ± 5,229.8 | 75,591.9 ± 31,798.1 | 2.64e-02 |  |  |  |
| CD70 | O55237 | 21948 | 94.3 ± 69.3 | 230.0 ± 71.6 | 3.45e-02 |  |  |  |
| IFNL3 | Q8CGK6 | 338374 | 8.4 ± 9.4 | 31.7 ± 16.0 | 4.62e-02 |  |  |  |
| PF4 | Q9Z126 | 56744 | 18,033.2 ± 4,758.2 | 43,012.3 ± 19,403.8 | 4.65e-02 |  |  |  |
| CCL4 | P14097 | 20303 | 184.1 ± 109.9 | 46.8 ± 6.9 | 4.70e-02 |  |  |  |
|  |  |  |  |  |  |  |  |  |
|  |  |  |  |  |  |  |  |  |
| CSF3 | P09920 | 12985 | 57.6 ± 19.5 | 135.1 ± 63.8 | 5.92e-02 |  |  |  |
| EREG | Q61521 | 13874 | 38.7 ± 26.1 | 6.0 ± 11.0 | 6.07e-02 |  |  |  |
| F11R | O88792 | 16456 | 5.3 ± 4.3 | 0.3 ± 0.7 | 6.37e-02 |  |  |  |
| CSF2RB | P26955 | 12983 | 145.3 ± 51.0 | 280.2 ± 108.5 | 6.52e-02 |  |  |  |
| FLT4 | P35917 | 14257 | 50.1 ± 45.1 | ND | 6.84e-02 |  |  |  |
| VCAM1 | P29533 | 22329 | 1,573.1 ± 146.3 | 2,117.8 ± 470.6 | 6.91e-02 |  |  |  |
| IL1B | P10749 | 16176 | 32.1 ± 30.2 | ND | 7.80e-02 |  |  |  |
| IL6 | P08505 | 16193 | 6.2 ± 6.2 | 48.9 ± 40.6 | 8.34e-02 |  |  |  |
| TYRO3 | P55144 | 22174 | 1.6 ± 1.6 | ND | 8.89e-02 |  |  |  |
| GAS1 | Q01721 | 14451 | 551.0 ± 147.3 | 399.0 ± 28.1 | 8.90e-02 |  |  |  |
| CXCL2 | P10889 | 20310 | 52.9 ± 23.3 | 29.2 ± 2.8 | 9.01e-02 |  |  |  |
| PSPN | O70300 | 19197 | 1.9 ± 0.2 | 5.9 ± 4.2 | 1.02e-01 |  |  |  |
| IL25 | Q8VHH8 | 140806 | ND | 52.4 ± 55.7 | 1.09e-01 |  |  |  |
| IL10 | P18893 | 16153 | 90.8 ± 48.1 | 189.2 ± 93.0 | 1.09e-01 |  |  |  |
| CCL24 | Q9JKC0 | 56221 | ND | 26.5 ± 28.3 | 1.10e-01 |  |  |  |
| MMP10 | O55123 | 17384 | 5.3 ± 0.5 | 6.0 ± 0.6 | 1.21e-01 |  |  |  |
| PGF | P49764 | 18654 | 96.2 ±15.9 | 142.1 ± 48.3 | 1.21e-01 |  |  |  |
| ANGPT4 | Q9WVH6 | 11602 | 4,577.3 ± 603.5 | 5,956.4 ± 1429.2 | 1.26e-01 |  |  |  |
| IL17B | Q9QXT6 | 56069 | 2,624.1 ± 1,683.4 | 4,668.0 ± 1,631.1 | 1.32e-01 |  |  |  |
| AHSG | P29699 | 11625 | 723.1 ± 780.4 | 5,760.6 5,751.6 | 1.33e-01 |  |  |  |
| CSF1 | P07141 | 12977 | 37.4 ± 32.5 | 9.1 ± 5.1 | 1.37e-01 |  |  |  |
| FRZB | P97401 | 20378 | 1,095 ± 732.7 | 461.0 ± 218.7 | 1.48e-01 |  |  |  |
| IL2 | P04351 | 16183 | 18.3 ± 22.1 | ND | 1.49e-01 |  |  |  |
| ENG | Q63961 | 13805 | 31.7 ± 39.1 | ND | 1.56e-01 |  |  |  |
| EPGN | Q924X1 | 71920 | 3.7 ± 4.6 | ND | 1.57e-01 |  |  |  |
| IL17RB | Q9JIP3 | 50905 | 15,810.9 ± 3,544.4 | 23,058.8 ± 8,260.7 | 1.58e-01 |  |  |  |
| MFGE8 | P21956 | 17304 | 641.3 ± 446.9 | 180.9 ± 361.9 | 1.60e-01 |  |  |  |
| ANGPTL3 | Q9R182 | 30924 | 4,774.8 ± 3,641.3 | 8,310.9 ± 2,643.8 | 1.67e-01 |  |  |  |
| BTC | Q05928 | 12223 | 0.2 ± 0.4 | 3.9 ± 4.8 | 1.75e-01 |  |  |  |
| ADAMTS1 | P97857 | 11504 | 232.9 ± 77.9 | 308.6 ± 69.7 | 1.98e-01 |  |  |  |
| CCL6 | P27784 | 20305 | 630.4 ± 432.0 | 1,042.3 ± 371.2 | 1.98e-01 |  |  |  |
| CCL28 | Q9JIL2 | 56838 | 35.0 ± 52.4 | ND | 2.29e-01 |  |  |  |
| FASL | P41047 | 14103 | 67.6 ± 20.6 | 120.9 ± 77.4 | 2.32e-01 |  |  |  |
| TNF | P06804 | 21926 | ND | 22.7 ± 34.3 | 2.34e-01 |  |  |  |
| KDR | P35918 | 16542 | 7.8 ± 2.6 | 19.3 ± 17.5 | 2.42e-01 |  |  |  |
| INHBA | Q04998 | 16323 | 144.7 ± 24.0 | 170.0 ± 33.7 | 2.67e-01 |  |  |  |
| RETN | Q99P87 | 57264 | ND | 1.5 ± 2.5 | 2.78e-01 |  |  |  |
| HGF | Q08048 | 15234 | ND | 138.4 ± 235.8 | 2.85e-01 |  |  |  |
| CX3CL1 | O35188 | 20312 | 2.7 ± 4.6 | ND | 2.92e-01 |  |  |  |
| CD36 | Q08857 | 12491 | 481.7 ± 75.2 | 568.7 ± 134.4 | 3.02e-01 |  |  |  |
| CD40LG | P27548 | 21947 | 187.9 ± 32.7 | 118.5 ± 121.8 | 3.14e-01 |  |  |  |
| TNFRSF18 | O35714 | 21936 | ND | 2.6 ± 5.3 | 3.56e-01 |  |  |  |
| IL20 | Q9JKV9 | 58181 | ND | 1293.5 ± 2586.9 | 3.56e-01 |  |  |  |
| CCL22 | O88430 | 20299 | 1.9 ± 3.7 | ND | 3.56e-01 |  |  |  |
| SELP | Q01102 | 20344 | ND | 200.5 ± 400.9 | 3.56e-01 |  |  |  |
| THPO | P40226 | 21832 | ND | 9221.8 ± 18443.6 | 3.56e-01 |  |  |  |
| IFNG | P01580 | 15978 | 0.9 ± 1.8 | ND | 3.56e-01 |  |  |  |
| IL5 | P04401 | 16191 | 0.5 ± 0.9 | ND | 3.56e-01 |  |  |  |
| CCL1 | P10146 | 20290 | ND | 2.2 ± 4.4 | 3.56e-01 |  |  |  |
| MET | P16056 | 17295 | 7.5 ± 15.0 | ND | 3.56e-01 |  |  |  |
| LEPR | P48356 | 16847 | 3.9 ± 7.7 | ND | 3.56e-01 |  |  |  |
| TNFSF11 | O35235 | 21943 | 0.3 ± 0.5 | ND | 3.56e-01 |  |  |  |
| IL3 | P01586 | 16187 | 20.0 ± 16.1 | 33.5 ± 22.5 | 3.67e-01 |  |  |  |
| DKK1 | O54908 | 13380 | 1,087.1 ± 120.7 | 1,409.5 ± 717.9 | 4.10e-01 |  |  |  |
| VEGFD | P97946 | 14205 | 0.9 ± 1.1 | 2.0 ± 2.4 | 4.22e-01 |  |  |  |
| SLAMF1 | Q9QUM4 | 27218 | 293.5 ± 13.5 | 415.7 ± 288.8 | 4.30e-01 |  |  |  |
| VEGFB | P49766 | 22340 | 12.1 ± 17.8 | 32.6 ± 48.6 | 4.58e-01 |  |  |  |
| SHH | Q62226 | 20423 | 71.1 ± 25.8 | 50.2 ± 49.1 | 4.64e-01 |  |  |  |
| CXCL13 | O55038 | 55985 | 119.4 ± 21.3 | 154.3 ± 93.7 | 4.95e-01 |  |  |  |
| TNFRSF8 | Q60846 | 21941 | 2.4 ± 1.2 | 8.9 ± 4.4 | 5.06e-01 |  |  |  |
| IL6ST | Q00560 | 16195 | 131.8 ± 17.5 | 113.9 ± 93.0 | 5.26e-01 |  |  |  |
| ARTN | Q920L2 | 11876 | 20.5 ± 6.5 | 26.0 ± 15.7 | 5.38e-01 |  |  |  |
| TSLP | Q9JIE6 | 53603 | 569.4 ± 184.3 | 787.2 ± 707.8 | 5.73e-01 |  |  |  |
| IL13 | P20109 | 16163 | 305.7 ± 49.1 | 341.0 ± 131.5 | 6.32e-01 |  |  |  |
| CCL5 | P30882 | 20304 | 31.6 ± 3.1 | 34.3 ± 10.4 | 6.37e-01 |  |  |  |
| CHRD | Q920E2 | 12667 | 1713.1 ± 158.2 | 1906.0 ± 766.4 | 6.40e-01 |  |  |  |
| TNFSF4 | P43488 | 22164 | 40.3 ± 37.7 | 53.5 ± 50.3 | 6.88e-01 |  |  |  |
| CCL21B | P86792 | 100042493 | 395.0 ± 243.7 | 369.5 ± 305.1 | 6.99e-01 |  |  |  |
| IL15 | P48346 | 16168 | 967.9 ± 360.6 | 1193.3 ± 769.0 | 7.60e-01 |  |  |  |
| LGALS7 | O54974 | 16858 | 3108.1 ± 1559.1 | 2806.3 ± 1484.7 | 7.89e-01 |  |  |  |
| IL21 | Q9ES17 | 60505 | 33.5 ± 15.9 | 44.8 ± 38.6 | 8.36e-01 |  |  |  |
| MOK | Q9WVS4 | 26448 | 65.8 ± 48.7 | 56.3 ± 112.5 | 8.81e-01 |  |  |  |
| CD40 | P27512 | 21939 | 22.0 ± 6.3 | 21.0 ± 21.0 | 9.30e-01 |  |  |  |
| CCL17 | Q9WUZ6 | 20295 | 20.1 ± 18.9 | 36.0 ± 21.8 | 9.35e-01 |  |  |  |
| ICAM1 | P13597 | 15894 | 61.9 ± 36.2 | 61.6 ± 59.3 | 9.45e-01 |  |  |  |
| CCL2 | P10148 | 20296 | 23.9 ± 18.6 | 28.9 ± 24.9 | 9.56e-01 |  |  |  |
|  |  |  |  |  |  |  |  |  |

*, adj, adjusted per 10^6^ cells;

n = 4 independent osteoblast cultures per genotype, derived from 2 males and 2 females;

ND, not detected


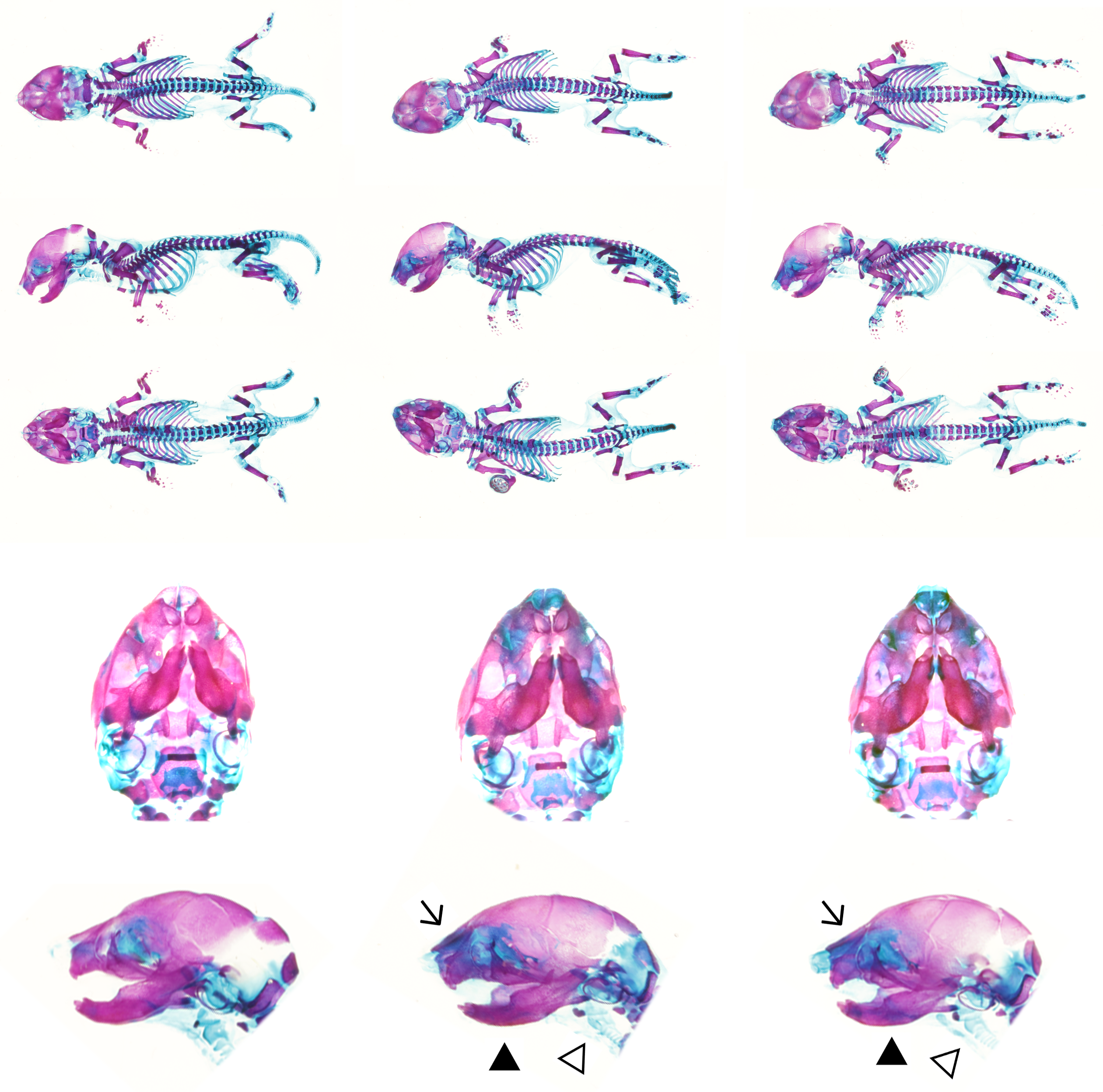


B

A

*Lmna^+/+^ Lmna^G609G/+^ Lmna^G609G/G609G^*

**Figure S1. Mice expressing *Lmna*^G609G^ have altered bone development at birth.** (A) Whole skeletal staining of one day-old wild-type (*Lmna*^+/+^), heterozygous (*Lmna*^G609G/+^) and homozygous (*Lmna*^G609G/G609G^) mice with alizarin red S and alcian blue. Both heterozygous and homozygous mice exhibit narrower, bell-shaped rib cages, and increased ossification of tail vertebrae. (B) Staining of bone and cartilage structures in the skulls of newborn mice reveals decreased mineralization of the hyoid bone, altered parietal and interparietal ossification of the cranium, increased cartilage staining of the maxillary and premaxillary bones (arrow) and mandible (black arrowhead), and decreased tracheal/esophogeal cartilage (white arrowhead) in heterozygous and homozygous mice.


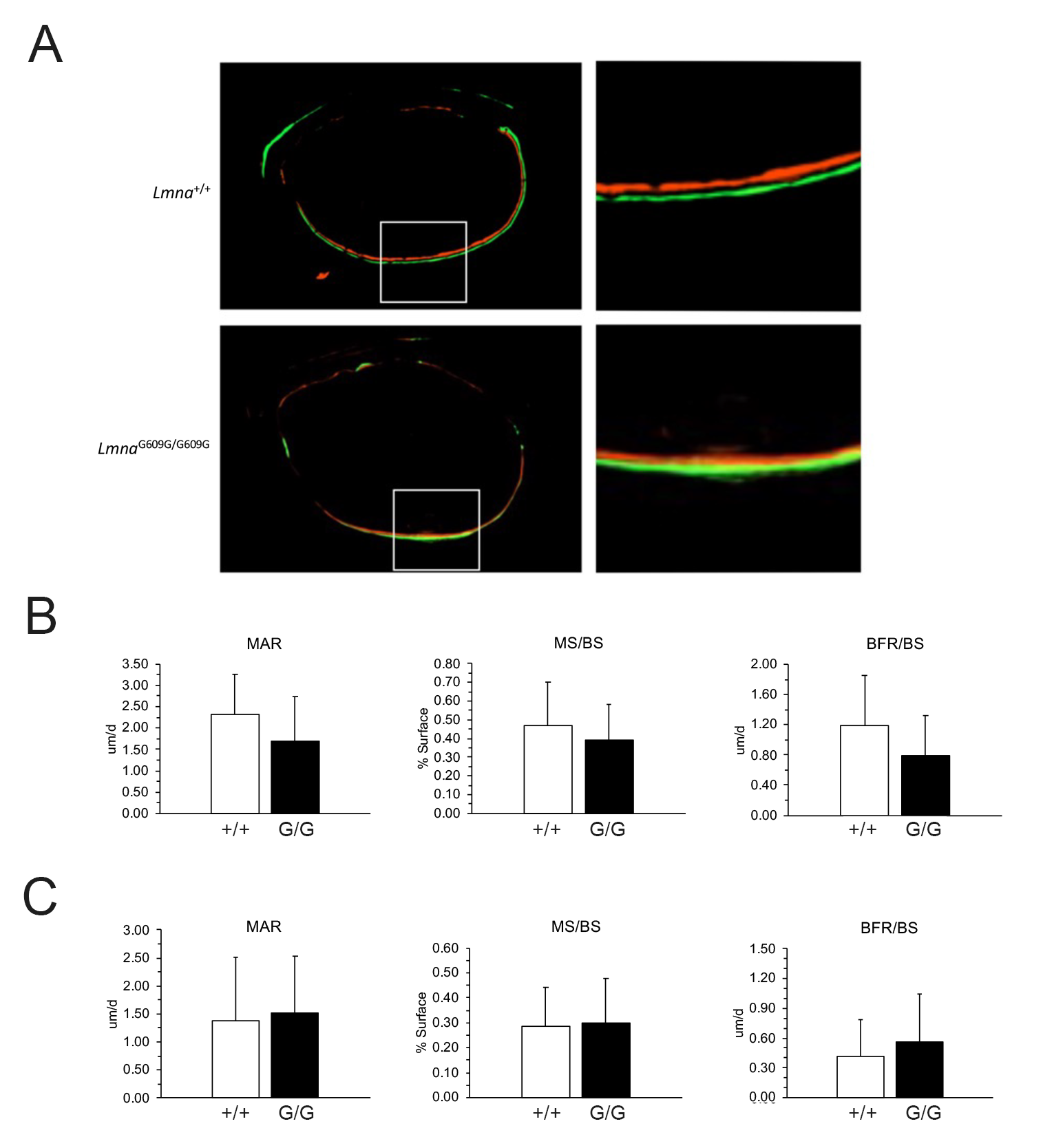


**Figure S2. Dynamic histomorphometric analysis reveals a trend in reduced bone formation rates on endosteal femoral surfaces in *Lmna*^G609G/G609G^ mice.** (A) Representative image of dual-labeled cortical bone surfaces shows calcein and alizarin deposition within mineralizing fronts at eight and nine weeks of age, respectively. (B) Although endosteal mineralizing surface per bone surface (MS/BS) was equivalent, mineral apposition rate (MAR) and bone formation rate per bone surface (BFR/BS) was decreased in *Lmna*^G609G/G609G^ versus *Lmna*^+/+^ mice by 27% (p<0.09) and 33% (p<0.08), respectively. (C) No differences were observed for MAR, MS/BS or BFR/BS on periosteal surfaces. N = 5 male and 8 female *Lmna*^+/+^, 6 male and 6 female *Lmna*^G609G/G609G^ mice.


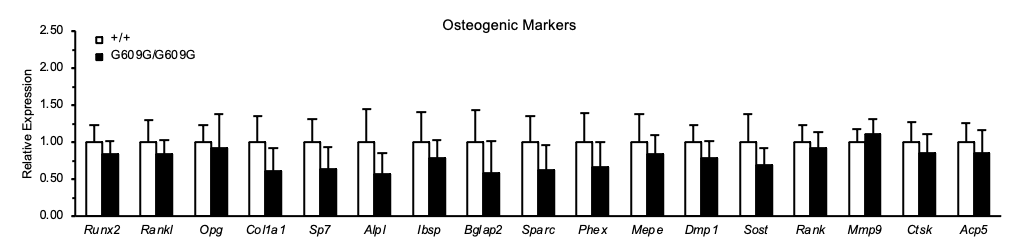

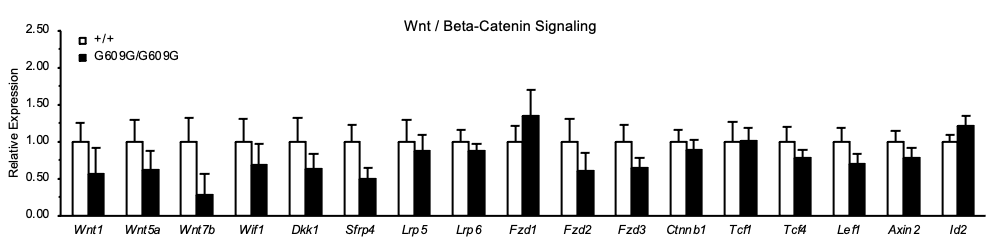

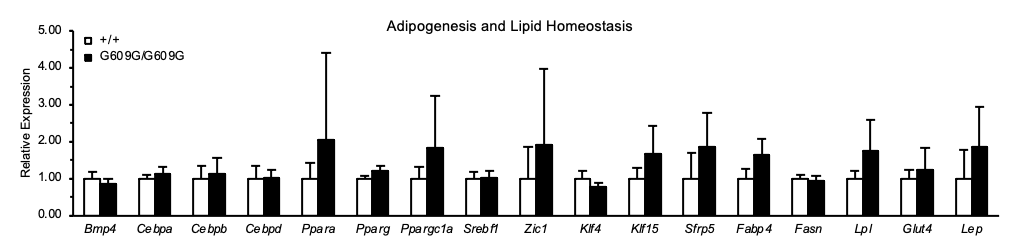


*

*

**

*

***

*

***

**

***

**

**

**

***

*

A

B

*

*

*

*

*

*

*

*

*

*

*

*

**

*

*

C

D


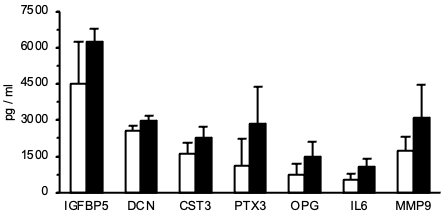

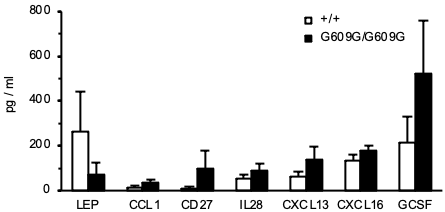


*

**

*

*

*

*

*

*

*

*

*

*

*

*

**Figure S3. *Lmna*^G609G/G609G^ mice have reduced osteogenic gene expression, increased adipogenic gene expression and elevated inflammatory cytokine levels.** (A-C) Quantitation of expression of osteogenic markers (A), WNT-beta catenin signaling pathways members (B) and genes involved in adipogenic differentiation and lipid homeostasis (C) in femoral cortical bone tissue by RNA sequencing. N = 12 per genotype (6 males, 6 females). (B) Plasma levels of inflammatory, mitogenic and chemotactic cytokines in 8-week mice. N=6 per genotype (3 males, 3 females); *, p < 0.05; **, p < 0.01; ***, p < 0.001


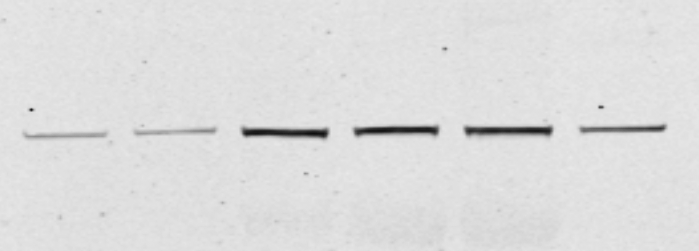

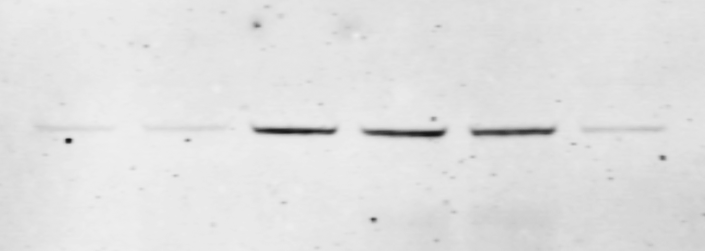

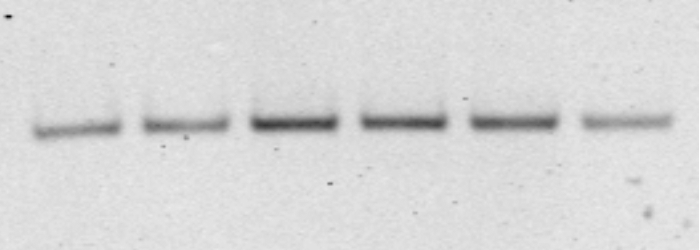

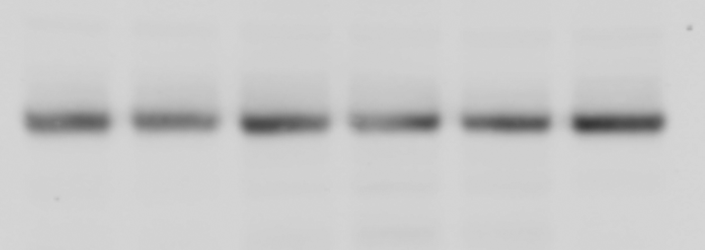

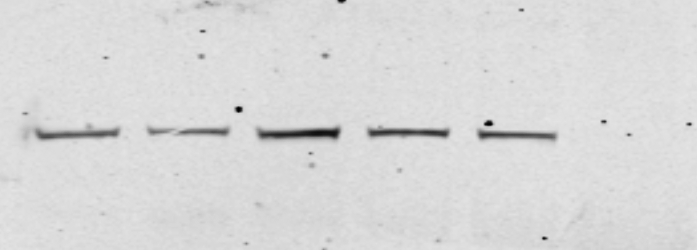

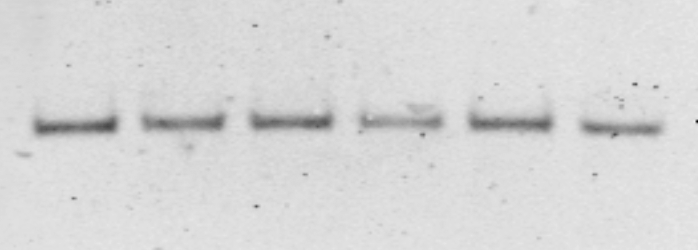

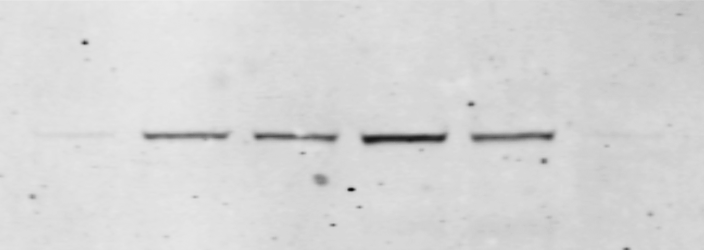

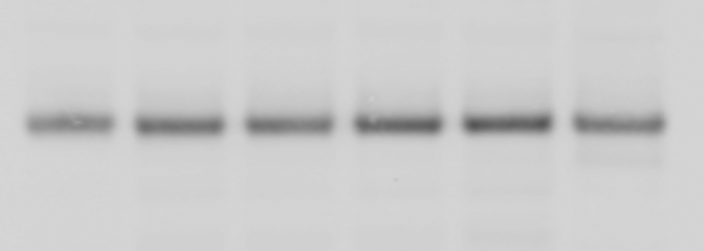


*Lmna^+/+^*

*Lmna^G609G/G609G^*

Active CTNNB1 -

S6 -

Active CTNNB1 -

S6 -


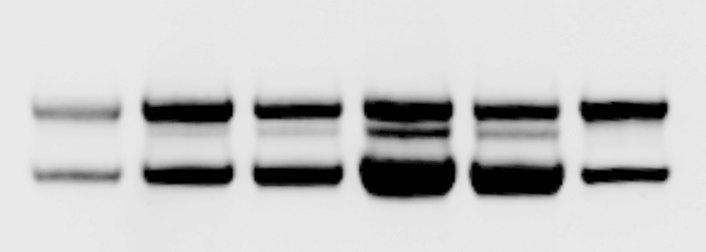

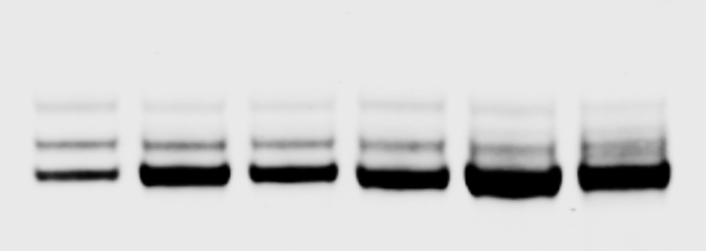

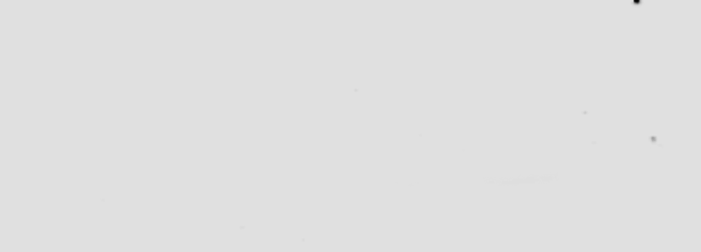

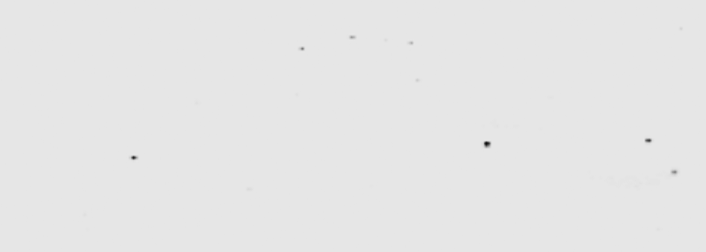


LMNA -

LMNC -

LMNA -

LMNC -

Progerin -

CYTOPLASM NUCLEUS

1

5

10

15

20

30

1

5

10

15

20

30

DAYS -

**Figure S4. Beta catenin signaling is inhibited in cultured *Lmna*^G609G/G609G^ osteoblasts.** Immunoblots of cytoplasmic and nuclear fractions isolated from wild-type (*Lmna*^+/+^) and homozygous (*Lmna*^G609G/G609G^) osteoblasts cultured in osteogenic differentiation media. Total intracellular active beta catenin (CTNNB1) was reduced at later timepoints of the differentiation timecourse but the relative fraction within the nucleus was equivalent in both genotypes. A-type lamins (LMNA, Progerin, LMNC) were used to validate the fractionation procedure. Ribosomal protein S6 was used as a loading control.

A

*Lmna^+/+^*

*Lmna^G/G^*


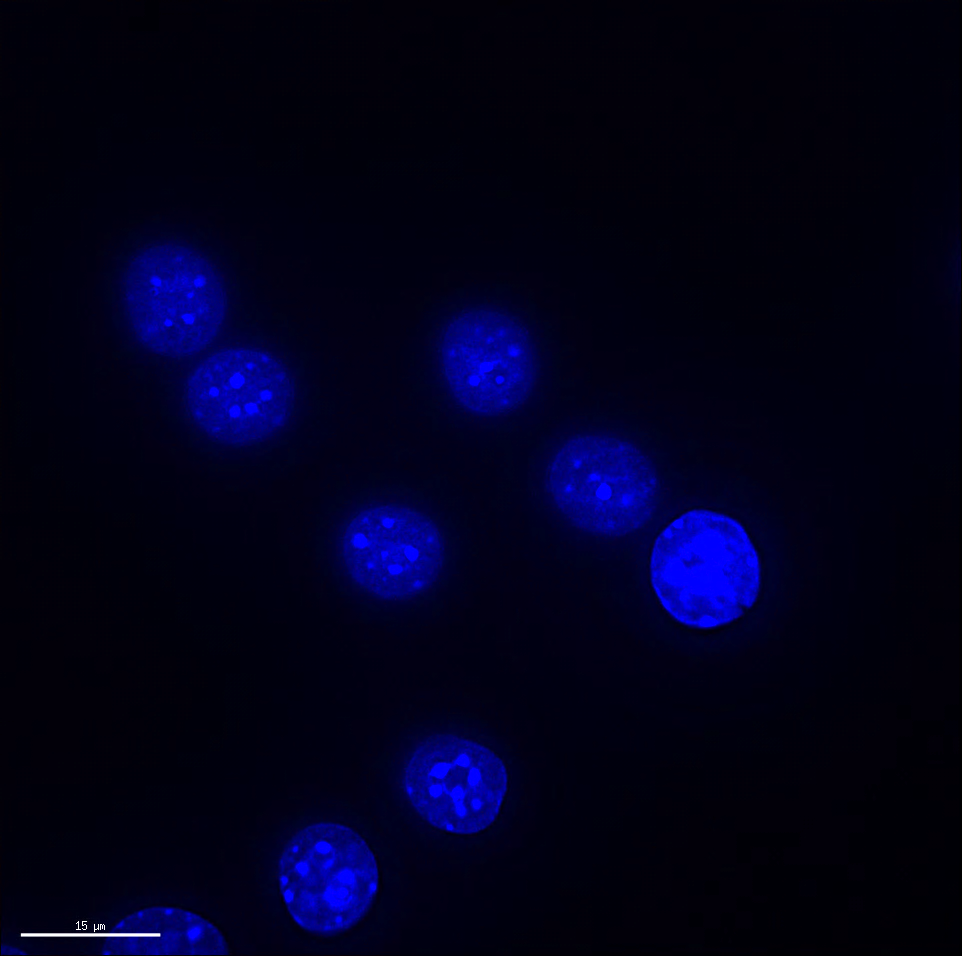

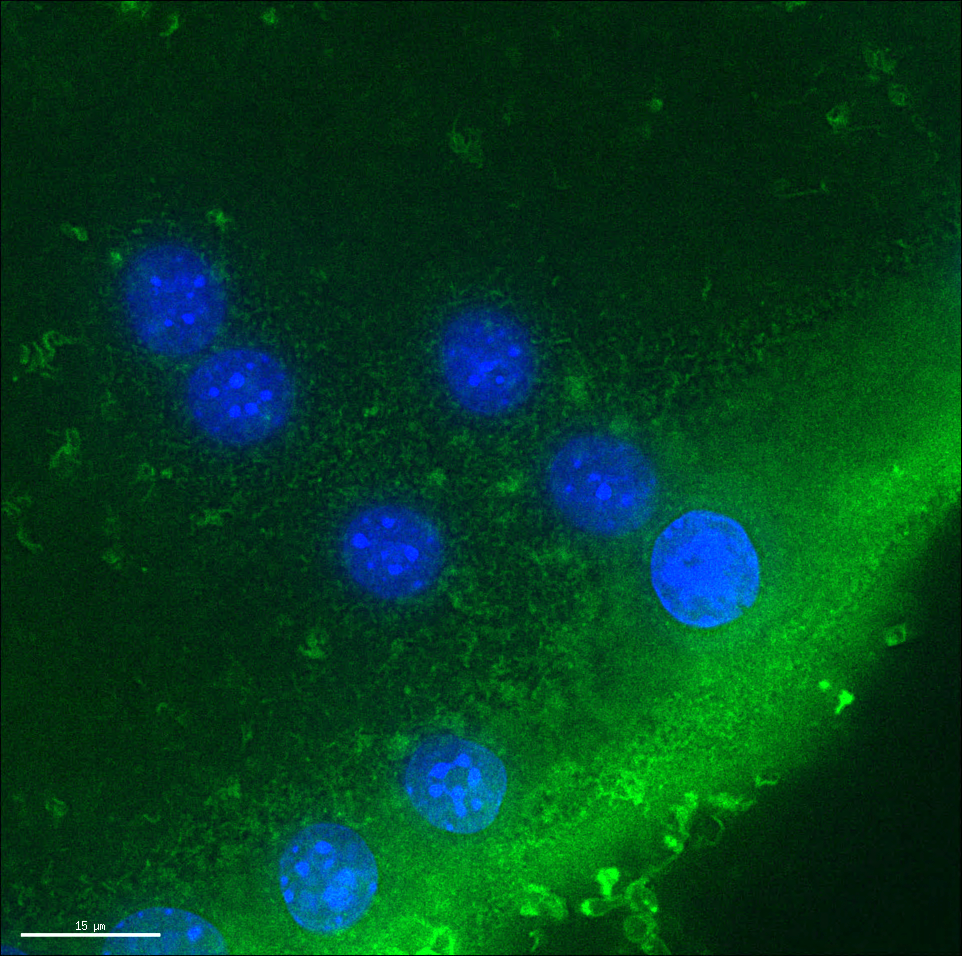

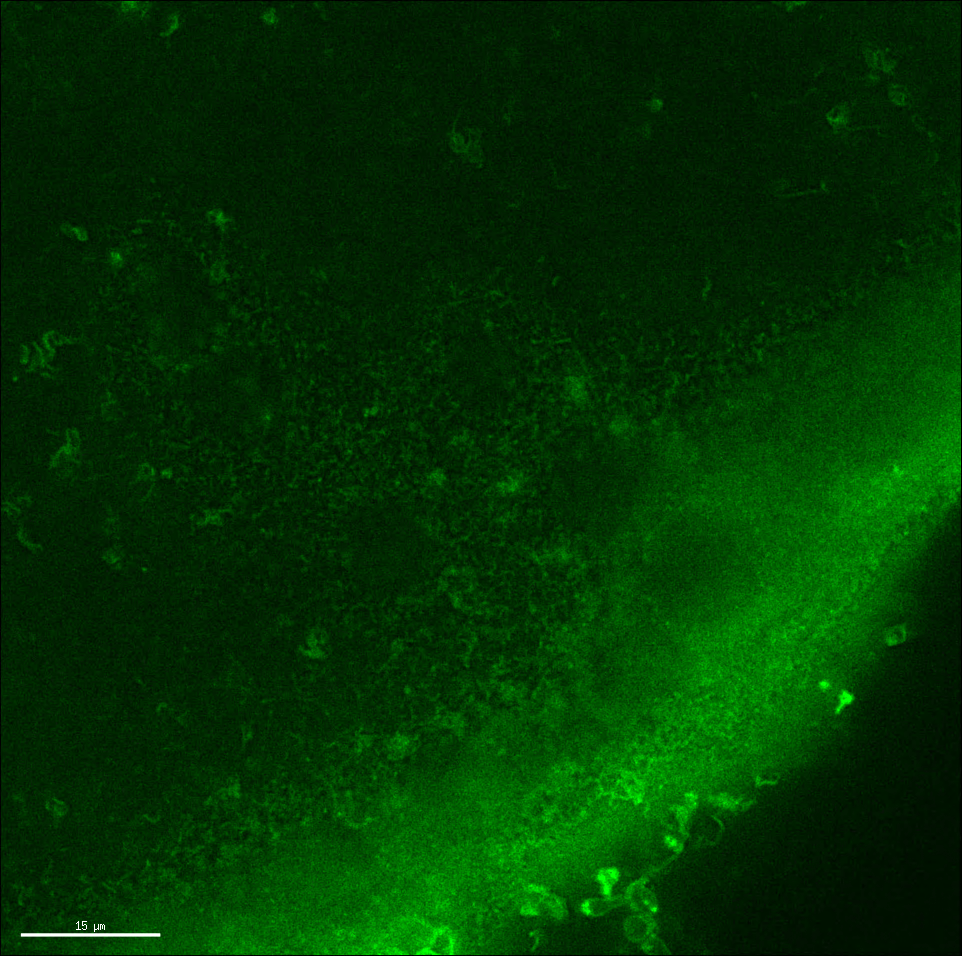


Actin

DAPI

Progerin

DAPI

Progerin

Actin

Progerin


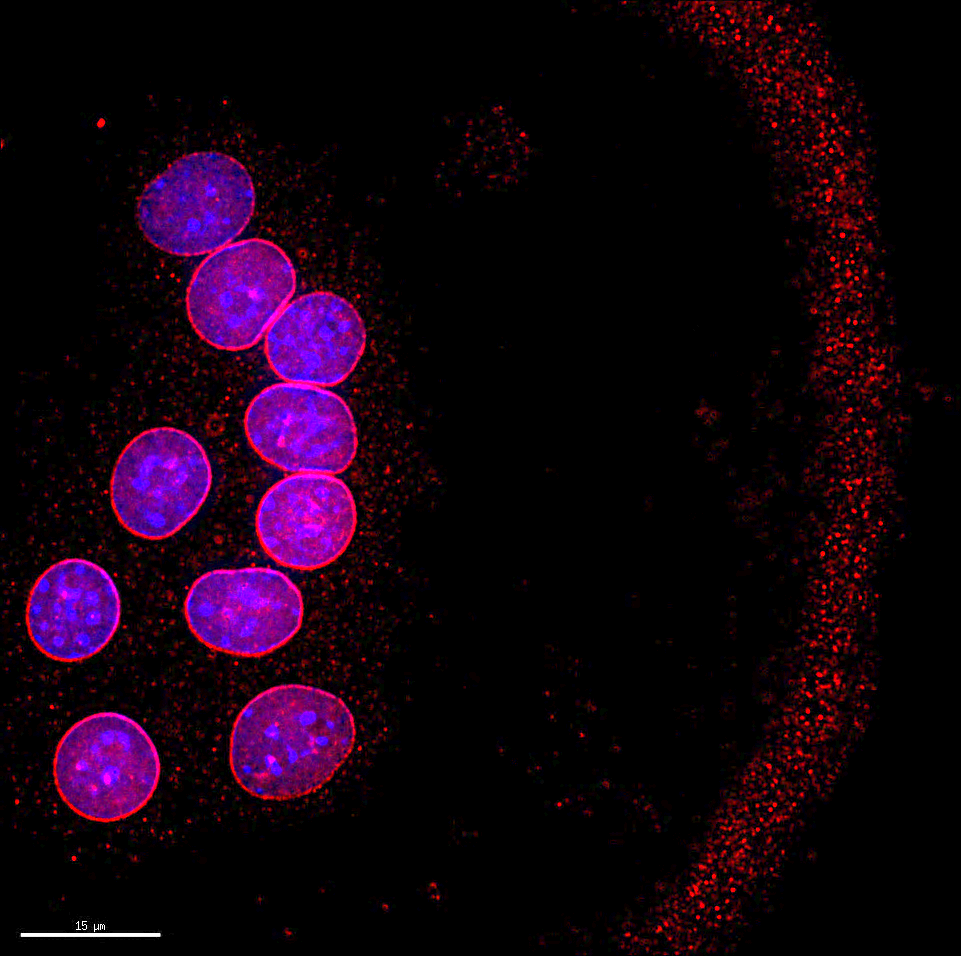

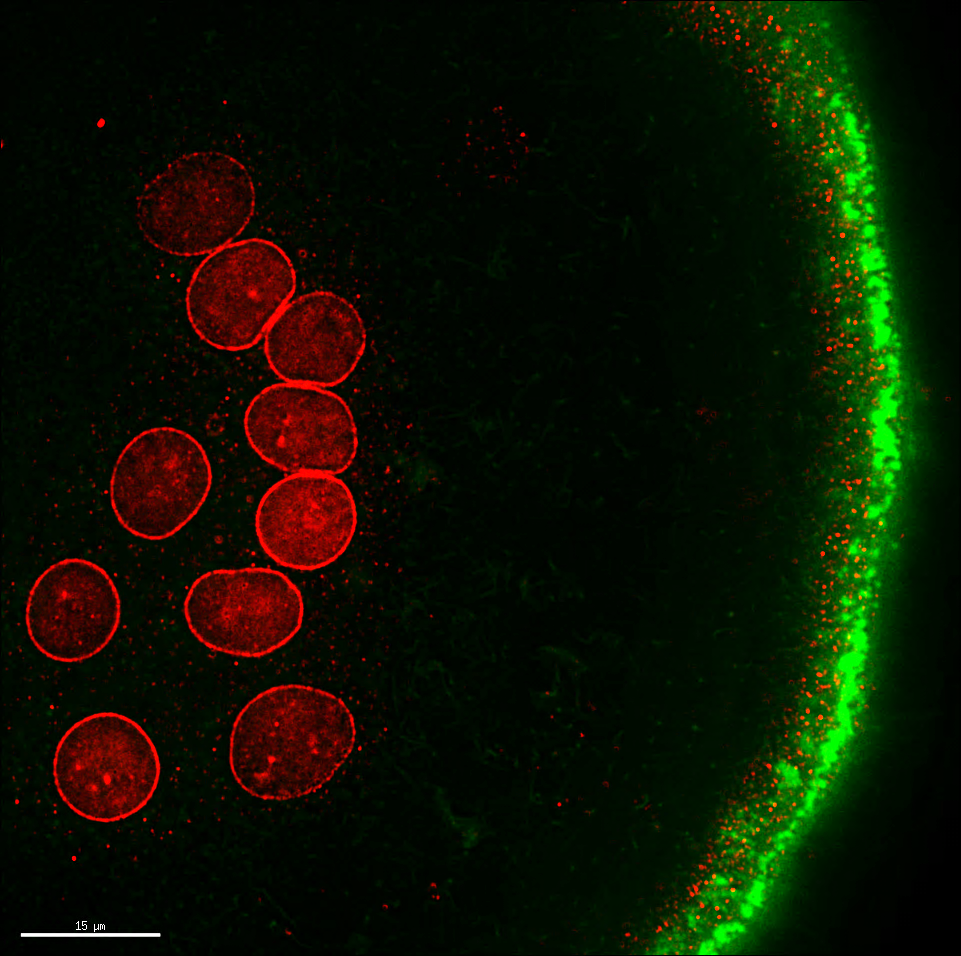

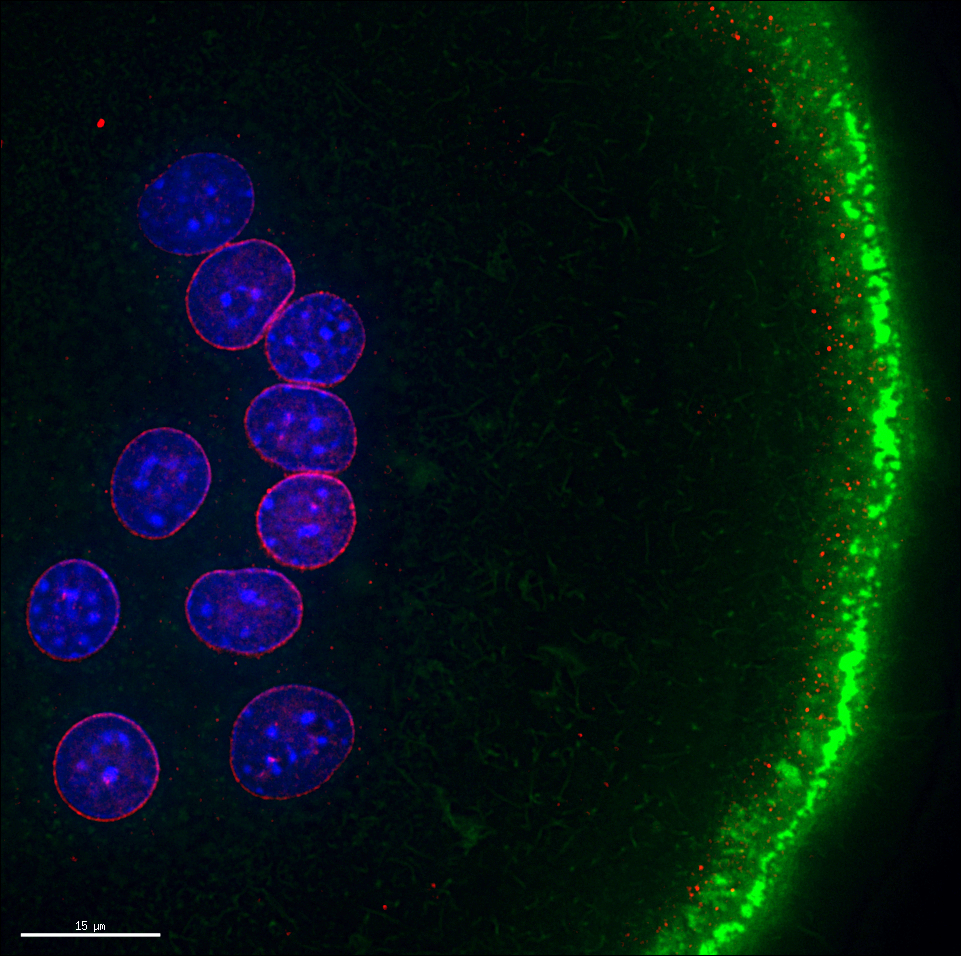


B

**
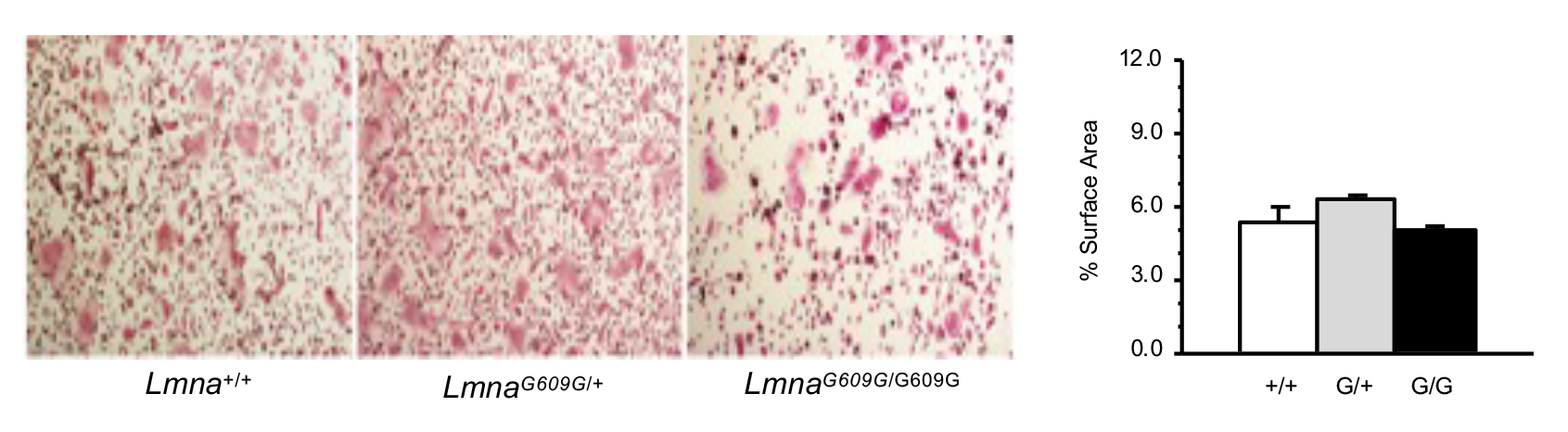
**

**Figure S5.** **Progerin is expressed in bone marrow derived osteoclasts.** (A) Progerin colocalizes to the nuclear lamina and actin ring underlying the plasma membrane in cultured osteoclasts. Wild-type (+/+) and *Lmna*^G609G/G609G^ (G/G) cells were stained to visualize nuclei (DAPI, blue), Actin (green) and Progerin (red). (B) TRAcP staining of cultured mature osteoclasts derived from wild-type (*Lmna*^+/+^), heterozygous (*Lmna*^G609G/+^) and homozygous (*Lmna*^G609G/G609G^) 8-week marrow progenitors. Surface area was calculated to quantitate formation *in vitro*. No differences were observed in three independent experiments.

**Supplemental References**

Benjamini Y & Hochberg Y (1995). Controlling the False Discovery Rate - a Practical and Powerful Approach to Multiple Testing. *J R Stat Soc B*. **57**, 289-300.

Cabral WA, Tavarez UL, Beeram I, Yeritsyan D, Boku YD, Eckhaus MA, . . . Collins FS (2021). Genetic reduction of mTOR extends lifespan in a mouse model of Hutchinson-Gilford Progeria syndrome. *Aging Cell*. **20**, e13457.

Cao K, Graziotto JJ, Blair CD, Mazzulli JR, Erdos MR, Krainc D & Collins FS (2011). Rapamycin reverses cellular phenotypes and enhances mutant protein clearance in Hutchinson-Gilford progeria syndrome cells. *Sci Transl Med*. **3**, 89ra58.

Dobin A, Davis CA, Schlesinger F, Drenkow J, Zaleski C, Jha S, . . . Gingeras TR (2013). STAR: ultrafast universal RNA-seq aligner. *Bioinformatics*. **29**, 15-21.

Hartley SW & Mullikin JC (2015). QoRTs: a comprehensive toolset for quality control and data processing of RNA-Seq experiments. *Bmc Bioinformatics*. **16**.

Korotkevich G, Sukhov V, Budin N, Shpak B, Artyomov MN & Sergushichev A (2021). Fast gene set enrichment analysis. *bioRxiv*, 060012.

Love MI, Huber W & Anders S (2014). Moderated estimation of fold change and dispersion for RNA-seq data with DESeq2. *Genome Biol*. **15**, 550.

McClintock D, Gordon LB & Djabali K (2006). Hutchinson-Gilford progeria mutant lamin A primarily targets human vascular cells as detected by an anti-Lamin A G608G antibody. *Proc Natl Acad Sci U S A*. **103**, 2154-2159.

Osorio FG, Navarro CL, Cadinanos J, Lopez-Mejia IC, Quiros PM, Bartoli C, . . . Lopez-Otin C (2011). Splicing-directed therapy in a new mouse model of human accelerated aging. *Sci Transl Med*. **3**, 106ra107.

Sinder BP, Eddy MM, Ominsky MS, Caird MS, Marini JC & Kozloff KM (2013). Sclerostin antibody improves skeletal parameters in a Brtl/+ mouse model of osteogenesis imperfecta. *J Bone Miner Res*. **28**, 73-80.

Sinder BP, Salemi JD, Ominsky MS, Caird MS, Marini JC & Kozloff KM (2015). Rapidly growing Brtl/+ mouse model of osteogenesis imperfecta improves bone mass and strength with sclerostin antibody treatment. *Bone*. **71**, 115-123.

Terajima M, Taga Y, Chen Y, Cabral WA, Hou-Fu G, Srisawasdi S, . . . Yamauchi M (2016). Cyclophilin-B Modulates Collagen Cross-linking by Differentially Affecting Lysine Hydroxylation in the Helical and Telopeptidyl Domains of Tendon Type I Collagen. *J Biol Chem*. **291**, 9501-9512.

Yamauchi M, Taga Y, Hattori S, Shiiba M & Terajima M (2018). Analysis of collagen and elastin cross-links. *Methods Cell Biol*. **143**, 115-132.
